# Nontargeted Analysis Strategy for the Identification of Phenolic Compounds in Complex Technical Lignin Samples

**DOI:** 10.1002/cssc.202000951

**Published:** 2020-07-02

**Authors:** Jens Prothmann, Kena Li, Christian Hulteberg, Peter Spégel, Margareta Sandahl, Charlotta Turner

**Affiliations:** ^1^ Lund University Department of Chemistry Centre for Analysis and Synthesis P.O. Box 124 22100 Lund Sweden; ^2^ Lund University Department of Chemical Engineering P.O. Box 124 21100 Lund Sweden

**Keywords:** analytical methods, biomass, chromatography, mass spectrometry, supercritical fluids

## Abstract

Lignin is the second most abundant biopolymer in nature and a promising renewable resource for aromatic chemicals. For the understanding of different lignin isolation and conversion processes, the identification of phenolic compounds is of importance. However, given the vast number of possible chemical transformations, the prediction of produced phenolic structures is challenging and a nontargeted analysis method is therefore needed. In this study, a nontargeted analysis method has been developed for the identification of phenolic compounds by using an ultrahigh‐performance supercritical fluid chromatography–high‐resolution multiple stage tandem mass spectrometry method, combined with a Kendrick mass defect‐based classification model. The method is applied to a Lignoboost Kraft lignin (LKL), a sodium lignosulfonate lignin (SLS), and a depolymerized Kraft lignin (DKL) sample. In total, 260 tentative phenolic compounds are identified in the LKL sample, 50 in the SLS sample, and 77 in the DKL sample.

## Graphical abstract

**Never mind the phenolics**: A nontargeted analysis method for the identification of phenolic compounds in different complex technical lignin samples has been developed by using ultrahigh‐performance supercritical fluid chromatography–high‐resolution multiple stage mass spectrometry in combination with a Kendrick mass defect‐based classification model for data evaluation.

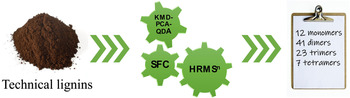

## Introduction

As the second most abundant biopolymer, lignin and can be found in all vascular plants and is one of the most promising renewable sources of aromatic chemicals today. There is a widespread belief that lignin will play an important role in the future of the biorefinery industry.^1^ Approximately 100 000 million tons of technical lignin is produced annually.^2^ However, less than 2 % of this is used for further conversion into value‐added products.^2^ One reason for this may be that current lignin isolation processes are mainly focused on carbohydrate valorization.^3^ Several different types of technical lignins are produced by using different isolation processes from the biomass, such as the Kraft or the organosolv process. The chemical complexity of technical lignins differs between the different possible combinations of lignin sources and isolation processes. Furthermore, technical lignins are often converted by chemical or biological processes into even more complex chemical mixtures to be used for specific applications or production of high‐value low‐molecular‐weight chemicals.^4^, ^5^

Interest in possible applications using lignin as a starting material has increased in recent years and several research groups in a variety of fields have introduced their ideas. Lignin valorization has the potential to replace completely fossil‐based aromatic polymers.^6^ By synthesizing two poly(ether amide)s from lignin‐derived precursors, Saenz and Scott showed that lignin‐based polymers are achievable.^7^ Other recent studies have shown that lignin could also be used to produce UV‐curable coatings,^8^ nanoparticles,^9^ elastomers,^10^ anticorrosion coating for metal surfaces,^11^ and carbon fibers.^12^ Chaleawlert‐umpon et al. and Mukhopadhyay et al. showed that lignin could also be used in the field of electrochemistry as an electrode material^13^ or as an electrolyte in flow batteries,^14^ respectively.

**Figure 1:**
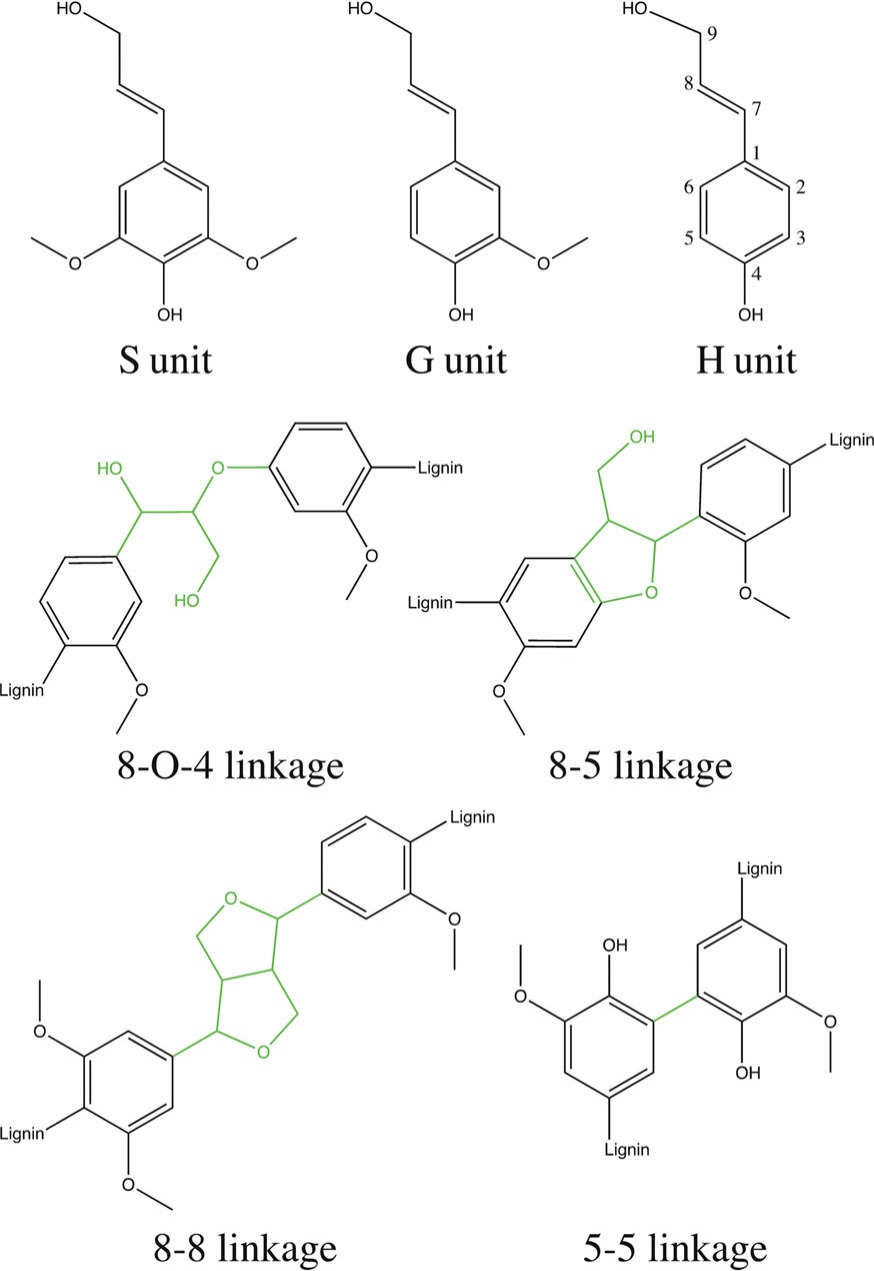
Monomeric subunits, syringyl (S), guaiacyl (G), and *p*‐coumaryl (H), and four common linkages (in green) of the chemical structure of lignin.

The chemical structure of the native lignin polymer is based on the three monomeric phenylpropanoid units, syringyl (S), guaiacyl (G) and *p*‐coumaryl (H; Figure 1). These monomers only differ in the degree of methoxylation of the aromatic ring. The content of S, G, and H units differs between plants. Whereas, for example, softwoods consist of >95 % G units and, with a few exceptions, contain no S units, hardwoods are composed of 25–50 % G units, 45–75 % S units and 0–8 % H units and grasses have a content of 5–35 % H units, 35–80 % G units and 20–55 % S units.^15^ The three subunits can be connected via different ether or carbon–carbon linkages. Some typical linkages are shown in Figure 1. During isolation processes and also by chemical or biological conversion of isolated lignin, the chemical structure of lignin is changed and the chemical complexity increases.^4^, ^16^, ^17^

For the optimization and understanding of conversion processes for technical lignins, reliable and, if possible, fast analysis methods are of importance. For lignin monomers (LMs), several analysis methods using mass spectrometry have been reported, including high‐performance liquid chromatography (HPLC)–high‐resolution mass spectrometry (HRMS),^18^ ultrahigh‐performance supercritical fluid chromatography (UHPSFC)–HRMS,^19^ and gas chromatography (GC)–MS.^20^ To analyze lignin oligomers (LOs), methods such as direct infusion (DI) MS and LC–MS have been developed during recent years. For example, Banoub et al. developed a method based on atmospheric pressure photo‐ionization (APPI)–HRMS for the identification of LOs in lignin isolated from wheat straw and proposed tentative structures for lignin dimers up to octamers.^21^ By using synthetic LOs, Morreel et al. developed an atmospheric pressure chemical ionization (APCI)–MS^*n*^ methods to investigate specific fragmentation pathways for LOs.^22^, ^23^ In combination with ultrahigh‐performance liquid chromatography (UHPLC), the developed APCI–MS^*n*^ method was used for the identification of LOs in wild‐type poplar xylem and in flax stems.^22^, ^24^

Owen et al. developed a method for the analysis of LMs and lignin dimers using HPLC–electrospray ionization (ESI)‐HRMS^*n*^.^18^ The developed method was used to identify and propose chemical structures for LMs and lignin dimers in lignin isolated from red oak^18^ and from switchgrass.^25^ In our laboratory, we have developed a method based on UHPLC–ESI‐HRMS^*n*^ for the identification of LOs in a Kraft lignin sample and tentative structures for lignin dimers, trimers, and tetramers were proposed.^26^

To our knowledge, UHPSFC has to date only been used in our laboratory for the analysis of LMs in lignin samples.^19^, ^27^, ^28^ We have shown that for LMs, UHPSFC provides good selectivity and chromatographic resolution for 40 lignin‐related phenolic model compounds in less than 6 min.^19^ However, to our knowledge, UHPSFC has not been used for the analysis of LOs. One reasons for this may be the lack of commercially available LO standards and that the synthesis of LOs is very time‐consuming. Without reference standards, a nontargeted analysis method for LOs need to have high identification confidence, which can be obtained by using HRMS for exact mass and chemical formula determination, alongside multiple‐stage tandem MS for structural elucidation. We recently introduced a nontargeted analysis method for LOs using UHPLC–ESI‐HRMS^*n*^ in combination with a pre‐selection strategy using data‐dependent neutral loss MS^3^ experiments combined with a principal component analysis‐quadratic discriminant analysis (PCA–QDA) classification model.^26^ With this method, tentative chemical structures for lignin dimers, trimers and tetramers were proposed. However, to our knowledge, a nontargeted analysis method using chromatographic separation in combination with HRMS^*n*^ covering LMs and LOs higher than dimers are currently lacking. Owen et al. showed that HPLC–ESI‐HRMS^*n*^ can be used for identification of unknown lignin dimers and proposed a tentative structure for one unknown dimer.^18^ Jarrell et al. extended this method from with a preselection strategy for LMs and dimers using *m*/*z* ranges, C/O ratios, and ring double‐bond (RDB) equivalents.^25^ With this method, several LMs and lignin dimers cloud be identified in a organosolv switchgrass lignin sample.^25^

Herein, we present a nontargeted analysis method covering LMs and LOs in a single run using UHPSFC–HRMS^*n*^. The previously introduced PCA–QDA classification model for LOs was extended for LMs and updated with previously identified lignin‐related phenolic compounds from prior reports. Furthermore, the PCA–QDA classification model was improved by introducing five new variables based on Kendrick mass defects (KMDs) calculated with units corresponding to functional groups that are abundant in technical lignins. Three different technical lignin samples were analyzed; a Lignoboost Kraft lignin (LKL) sample, a sodium lignosulfonate lignin (SLS) sample, and a depolymerized Kraft lignin (DKL) sample.

## Results and Discussion

### Classification models

**Figure 2:**
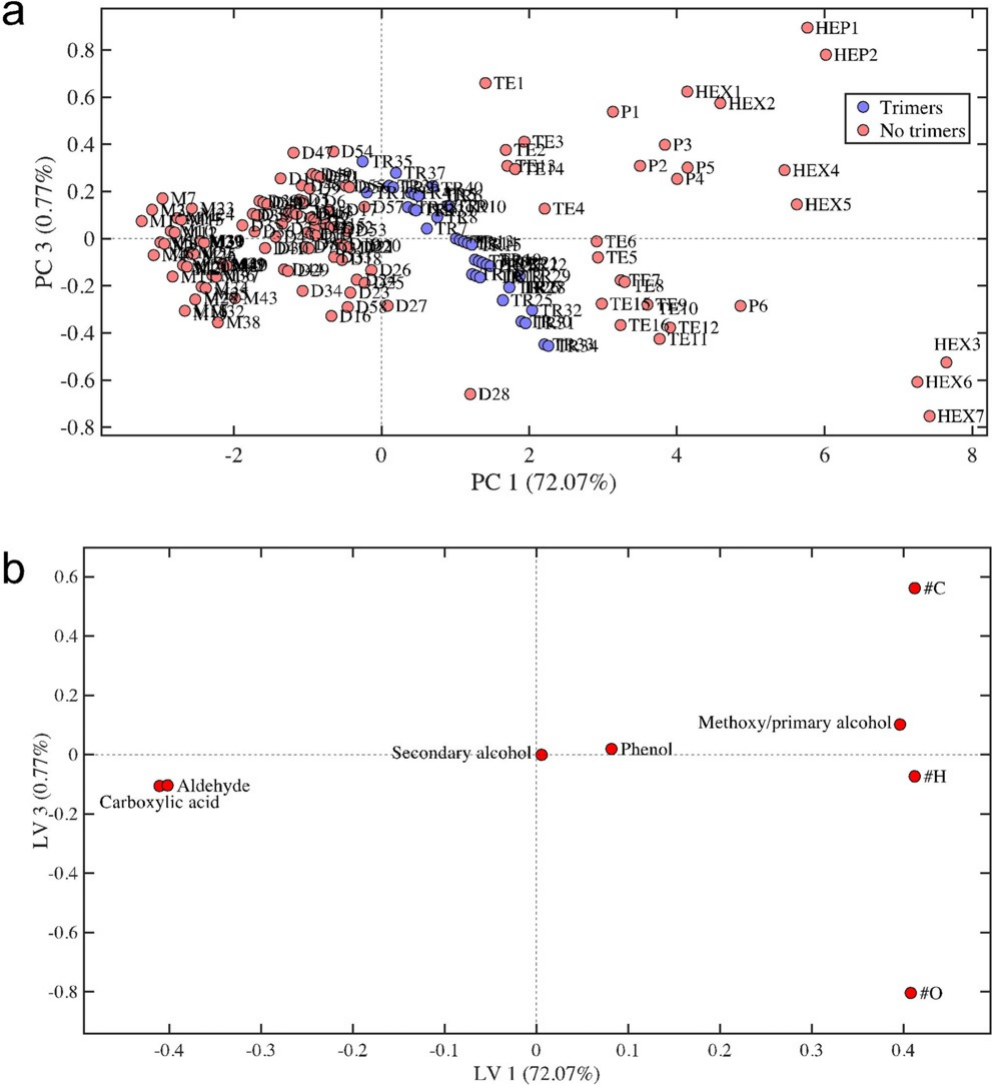
Score plot of the KMD–PCA–QDA classification model for lignin trimers (a) showing the principal components (PCs) **1** and **3** and the corresponding loading plot (b) showing the latent variables (LVs) **1** and **3**. M=monomers; D=dimers; TR=trimers; TE=tetramers; P=pentamers; HEX=hexamers; HEP=heptamers; #C=number of carbon atoms; #H=number of hydrogen atoms; #O=number of oxygen atoms. KMDs are indicated by their respective base unit functionality. Model validation results are given in Table S1.

Four KMD–PCA–QDA classification models were created based on the estimated list data; one for lignin monomers, one for lignin dimers, one for lignin trimers, and one for lignin tetramers. Since the same set of data was used for all classification models, the PCA score and loading plots are the same for all classification models. The PCA score and loading plots taken from the classification model for lignin trimers are exemplarily shown in Figure 2 a and 2 b, respectively. Additional score and loading plots are presented in the Supporting Information (Figures S1–S4). Model summaries, including number of components, obtained error rates, cross validation error rates, accuracies, and the number of false negatives for the four classification models are given in Table S1.

In the score plot in Figure 2 a, clusters for lignin monomers, dimers, trimers, and tetramers can be identified. Due to a lack of reported data, only the probable locations of clusters for pentamers, hexamers, and heptamers can be seen. The corresponding loading plot (Figure 2 b) illustrates that clustering along latent variable (LV) **1** is mainly dominated by the #H, #C, #O and the KMDs with base units for carboxylic acid, aldehyde, and methoxy/primary alcohol, whereas LV **3** is mainly dominated by #C and #O.

### Comparison of different technical lignin samples

**Figure 3:**
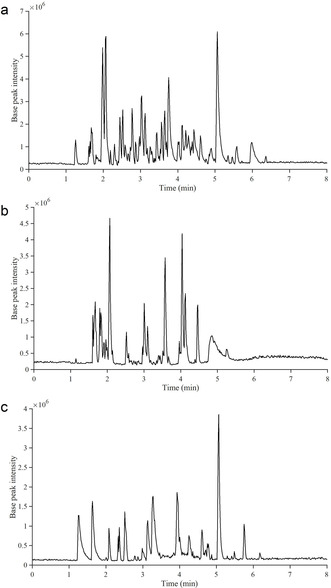
Base peak ion chromatograms of LKL (a), SLS (b), and DKL (c) samples obtained by using the developed UHPSFC–HRMS^*n*^ method.

Chromatographic separation of the LKL, SLS, and DKL samples was conducted in less than seven minutes (Figure 3). Furthermore, the obtained base peak ions (BPIs) indicate that the developed UHPSFC–ESI‐HRMS^*n*^ method is applicable to different technical lignin samples with different chemical properties.

The *m*/*z* values for the LKL, SLS, and DKL samples are summarized in Table 1. The highest number of phenolic compounds were identified in the LKL sample by using the peak list creation workflow and the preselection criteria.

**Table 1:** Overview of *m*/*z* values in the Lignoboost Kraft lignin (LKL), sodium lignosulfonate lignin (SLS), and depolymerized Kraft lignin (DKL) samples obtained by using the developed nontargeted analysis method for phenolic compounds.

Sample	LKL	SLS	DKL
Number of obtained *m*/*z* values^[a]^	372	132	113
Number of classified *m*/*z* values	211	67	58
Number of validated *m*/*z* values	120	29	44
Lignin monomers	15	14	10
Lignin dimers	78	13	34
Lignin trimers	23	2	0
Lignin tetramers	4	0	0
Number of *m*/*z* values matching reported data	51	11	22
Number of classified and validated *m*/*z* values with more than one retention time	73	12	20
Identified phenolic compounds	260	50	77
Lignin monomers	23	25	14
Lignin dimers	194	23	63
Lignin trimers	38	2	0
Lignin tetramers	5	0	0
Phenolic compounds with identification confidence level 1	5	6	7
Phenolic compounds with identification confidence level 2	21	7	9
Phenolic compounds with identification confidence level 3	234	37	61

[a] Using the peak list creation workflow and pre‐selection criteria.

**Figure 4:**
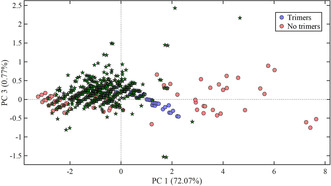
Same score plot of the KMD–PCA–QDA classification model for lignin trimers as shown in Figure 2 a including the projected positions of the 371 different *m*/*z* values (green stars) identified in the LKL sample using the peak list creation workflow and the preselection criteria. Model validation results are given in Table S1.

In the LKL sample, 372 different *m*/*z* values were found, whereas in the SLS and DKL samples, 132 and 113 *m*/*z* values, respectively, were found. Figure 4 shows the projected positions of the 372 obtained *m*/*z* values found in the LKL sample in the KMD–PCA–QDA model. Projections of the *m*/*z* values found in the SLS and DKL samples are presented in Figures S5 and S6, respectively. Most of the *m*/*z* values found in the LKL sample are projected on the lignin monomers, dimers, and trimers clusters, with the majority projected on the lignin dimers cluster. This also correlates with the distribution of classified and validated phenolic compounds in the sample (Table 1). For the classified *m*/*z* values, the detected ions, determined chemical formula, classifications, obtained mass differences, obtained ^13^C ratios, RDB equivalents, retention times, identified in‐source fragmentations, obtained MS^*n*^ data, estimated list matches, suggested compounds, and the obtained identification confidence levels of all identified phenolic compounds are given in Tables S2, S3, and S4 for the LKL, SLS, and DKL samples, respectively.

Obtained MS^2^ and MS^3^ fragmentations are shown in Tables S5–S78. In total, 211 out of the 372 *m*/*z* values obtained for LKL were classified as either lignin monomers, dimers, trimers, or tetramers. Out of these 211 *m*/*z* values, 120 passed the validation criteria, of which 15 were classified as lignin monomers, 78 as dimers, 23 as trimers, and 4 as tetramers. For the SLS and DKL samples, 67 and 58 *m*/*z* values, respectively, were classified as either monomers, dimers, or trimers. In both the SLS and the DKL samples, no *m*/*z* values were classified as lignin tetramers. The validation criteria were passed by 29 *m*/*z* values (14 monomers, 13 dimers, and 2 trimers) from the SLS sample and 44 *m*/*z* values (10 monomers, 34 dimers) from the DKL sample. In all samples, no phenolic compounds higher than a tetramer were identified. Reasons for this may be that they are not present in the sample or that the used ESI settings are not suitable for such compounds. Several of the *m*/*z* values classified and validated in the three different samples have previously been reported. In the LKL sample, we found 51 *m*/*z* values that were already reported, with 11 such values in the SLS sample and 22 in the DKL sample. Several of the classified and validated *m*/*z* values appear at more than one retention time. This was found for 73 *m*/*z* values in the LKL sample, 12 in the SLS sample, and 20 in the DKL sample. This indicated that many structural isomers of the classified and validated *m*/*z* values might be present, which makes the identification and structural investigation of phenolic compounds even more challenging.

Taking all retention times of all classified and validated *m*/*z* values into account, 260 tentative phenolic compounds were identified in the LKL sample, including 23 monomers, 194 dimers, 38 trimers, and 5 tetramers. Out of these, 5 reached identification confidence level 1, 21 level 2, and 234 level 3 (see Experimental Section for explanation of confidence levels). In the SLS, 50 tentative phenolic compounds were identified, including 25 monomers, 23 dimers, and 2 trimers. Out of these, 6 were identified with identification confidence level 1, 7 with level 2, and 37 with level 3. For the DKL sample, 77 tentative phenolic compounds were identified, with 14 being monomers and 63 being dimers. From the 77 tentative phenolic compounds, 7 reached identification confidence level 1, 9 reached level 2, and 61 reached level 3.

In the LKL, most identified phenolic compounds are dimers, followed by trimers, then monomers, and then tetramers. The high number of identified dimers could be due to condensation reactions of monomers during the isolation process. Tetramers were identified only in the LKL sample. Beside two trimers, mainly monomers and dimers were identified in the SLS sample. In contrast to the LKL sample, phenolic dimers were not predominant in the SLS sample. In the DKL sample, only monomers and dimers were identified, with dimers being the predominant group. Presumably, this observation could also be explained by condensation reactions of monomers during the depolymerization process.

### Characterization of unknown phenolic compounds

In all three lignin samples, several lignin monomers could be identified with identification confidence level 1 by comparing with reference standards. In the LKL sample, 4‐hydroxyacetophenone, vanillin, acetovanillone, vanillic acid, and coniferyl aldehyde were identified, whereas in the SLS sample 4‐hydroxybenzoic acid, vanillin, 4‐coumaric acid, vanillic acid, coniferyl aldehyde, and 4‐methoxycinnamic acid and in the DKL sample 4‐hydroxybenzaldehyde, 4‐hydroxyacetophenone, 4‐hydroxybenzoic acid, vanillin, acetovanillone, syringaldehyde, and acetosyringone were identified.

**Scheme 1:**
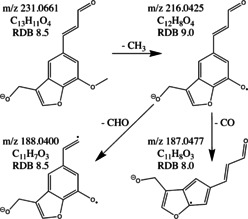
Proposed chemical structure and MS^3^ fragmentation pathway for the tentative lignin monomer with *m*/*z* 231.0661 detected in the LKL sample.

For some classified and validated lignin monomers identified with confidence level 2 or 3, possible compounds could be suggested based on the determined chemical formula, RDB equivalent, retention time and, if available, in‐source fragmentation and MS^2^ or MS^3^ fragmentation patterns (Tables S2–S4). For example, for the tentative lignin monomer with *m*/*z* 231.0661 in the LKL sample, a MS^3^ fragmentation was obtained and a chemical structure based on the determined chemical formula, RDB equivalent, and MS^3^ fragmentation pathway could be proposed (Scheme 1). Several lignin dimers and trimers were identified with identification level 2.

**Scheme 2:**
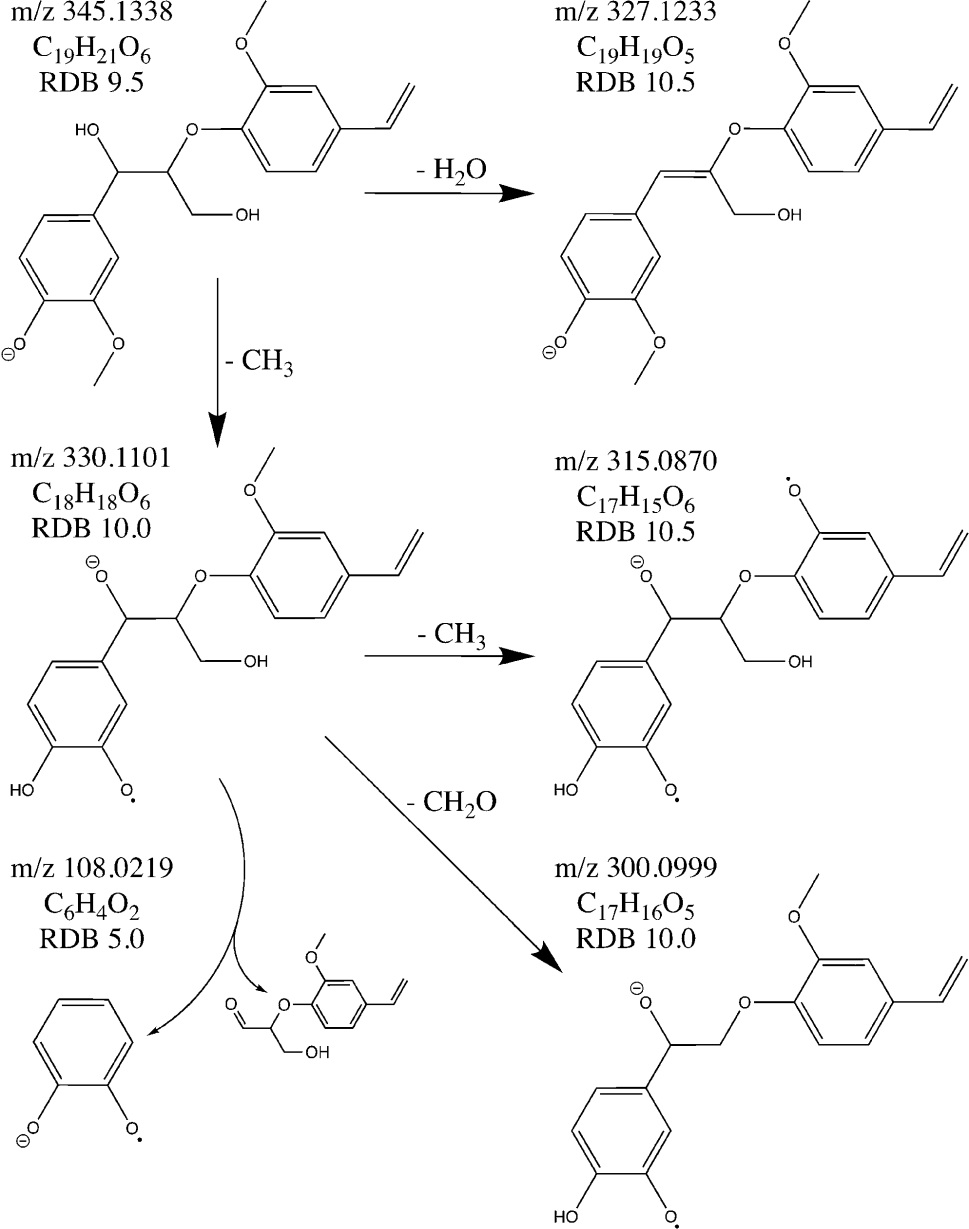
Proposed chemical structure and MS^3^ fragmentation pathway with a neutral loss including a benzene ring unit for the tentative lignin dimer with *m*/*z* 345.1338 detected in the LKL sample.

An important indicator for the identification of lignin oligomers is the loss of a benzene ring unit in MS^*n*^ experiments. For some of the lignin dimers and trimers, neutral loss of a benzene ring unit can be detected already at the MS^2^ stage, like for example for *m*/*z* 315.1233 in the LKL sample, where a fragment with *m*/*z* 178.0634 was detected, indicating a neutral loss of C_8_H_9_O_2_ (Table S25). Nonetheless, the probability of detecting neutral losses of benzene ring units increases with the number of MS^*n*^ stages. However, increased acquisition of higher MS^*n*^ stages also goes in hand with a more time‐consuming data evaluation. Therefore, data‐dependent neutral loss MS^3^ experiments to screen for typical neutral losses for lignin oligomers seems to be a reasonable compromise to obtain MS^3^ data of tentative lignin oligomers and the possibility to propose tentative chemical structures. Neutral loss of a benzene ring in a MS^3^ experiment is illustrated in Scheme 2 for the detected *m*/*z* 345.1338 in the LKL sample. At the MS^2^ stage, only less informative losses of methyl radicals and water molecules were detected. For compounds of particular interest, targeted MS^*n*^ experiments could be performed for more detailed structural elucidation.^26^

## Conclusions

The presented nontargeted UHPSFC–HRMS^*n*^ analysis method with a focus on phenolic compounds is applicable for the analysis of different types of lignin samples varying in chemical properties and pre‐treatments. A lignin sample can be analyzed to a great extent with only six injections, one full‐scan and five data‐dependent neutral loss MS^3^ experiments, each with a run time of 10 minutes. For the identification of phenolic compounds based on HRMS data, the developed KMD–PCA–QDA classification models for lignin monomers, dimers, trimers, and tetramers are useful preselection tools and furthermore increase the identification confidence. Several lignin monomers could be confirmed by comparison with reference standards, whereas for other identified tentative lignin monomers, chemical structures could be proposed. A high identification confidence can be achieved for lignin oligomers by MS^3^ experiments revealing a neutral loss including a benzene ring unit. The complexity of lignin samples could be illustrated by the identification of tentative isomers for several phenolic compounds.

## Experimental Section

### Chemicals

Ammonium formate (LC–MS grade) was obtained from Sigma–Aldrich (St. Louis, MO, USA). Acetone (HPLC grade) and acetonitrile (HPLC–MS grade) were purchased from VWR (Radnor, PA, USA), methanol (LC–MS grade) from J. T. Baker (Phillipsburg, NJ, USA), and ethyl acetate (LC–MS grade) from Merck (Darmstadt, Germany). Purified water was produced by using a Milli‐Q Water Purification System with a UV unit.

### Nontargeted analysis strategy

**Figure 5:**
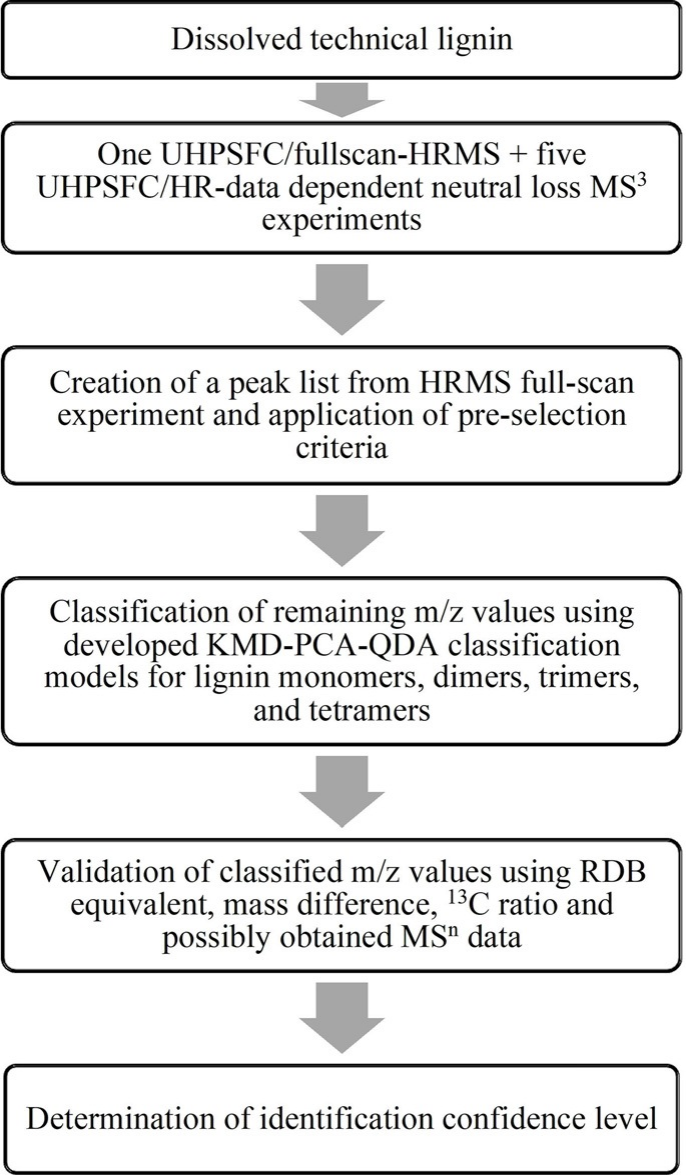
Schematic overview of the developed nontargeted analysis strategy for phenolic compounds in technical lignin samples.

An overview of the developed nontargeted analysis strategy for the identification of phenolic compounds in technical lignin samples is given in Figure 5. The details of each step of the analysis strategy are explained in the following paragraphs. In short, technical lignin is first dissolved in a suitable solvent, followed by one analysis using UHPSFC–full‐scan HRMS and five UHPSFC–HR data‐dependent neutral loss MS^3^ experiments. A peak list is created from the full‐scan MS data by using MZmine 2 followed by application of preselection criteria to reduce the number of *m*/*z* values. The remaining *m*/*z* values are classified by using the developed KMD–PCA–QDA classification models for lignin monomers, dimers, trimers, and tetramers. All classified *m*/*z* values are validated by using RDB equivalents, mass difference, ^13^C ratios, and, if available, also by MS^*n*^ data. Finally, the identification confidence level of each identified compound is determined.

### Samples

A solid Lignoboost Kraft lignin (LKL) sample was kindly provided by Jerk Rönnols (RISE, Sweden) and a solid sodium lignosulfonate lignin (SLS) sample was obtained from Domsjö Fabriker AB (Örnsköldsvik, Sweden). Both samples were dissolved in acetone/water (70:30 v/v) with a concentration of 33.3 mg mL^−1^. A depolymerized Kraft lignin (DKL) sample was produced from a mixture of softwood and hardwood lignin by base‐catalyzed depolymerization (BCD) and ultrafiltration of the black liquor retentate, as previously described.^29^ The BCD experiments were performed in a continuous‐flow reactor setup at 170 °C, 130 bar, and a residence time of 2 min. An Alfa Laval membrane GR95PP with a molecular weight cut‐off value of 2 kDa was used for filtering the depolymerized lignin sample. After ultrafiltration, the permeate was neutralized to pH 7 with 6 n hydrochloric acid. The aqueous fraction was separated from the remaining solids by centrifugation. Phenolic compounds were extracted from 1 mL of the DKL sample by liquid–liquid extraction with ethyl acetate (2 mL). All samples were centrifuged for 10 min at 20 °C and 14 000 rpm before injection. Solvent blank samples were prepared in the same way as the samples.

### Equipment

All experiments were performed on a Waters Ultra Performance Convergence Chromatography System (Waters, Milford, MA, USA) equipped with a diode array detector (ACQUIY UPC^2^ PDA detector, Waters). The UHPSFC system was connected via a flow splitter (ACQUITY UPC^2^ splitter, Waters) to an LTQ Orbitrap Velos Pro mass spectrometer (Thermo Scientific, Waltham, MA, USA). The MS system was equipped with a heated electrospray ionization source (HESI; Thermo Scientific). A Torus DIOL column (3 mm×100 mm, 1.7 μm) equipped with a Torus DIOL VanGuard pre‐column (2.1 mm×5 mm, 1.7 μm) was purchased from Waters. A 5424R Eppendorf centrifuge (Eppendorf, Hamburg, Germany) was used for sample centrifugation.

### Ultrahigh‐performance supercritical fluid chromatography–high‐resolution multiple stage tandem mass spectrometry

The settings for the UHPSFC–HRMS method were based on two slightly modified methods previously developed by our group.^19^, ^26^ A DIOL column was used for sample separation, with a column temperature of 50 °C, a flow rate of 2 mL min^−1^, a back pressure of 130 bar, and an injection volume of 1.5 μL. After each injection, the injection syringe and injection needle were washed with acetonitrile/water (1:1 v/v, 4 mL) followed by methanol (4 mL). Gradient elution was performed by using CO_2_ (**A**) and methanol (**B**) as solvents. The gradient started with 0 vol. % **B**. During the first 2.5 min, **B** was ramped up to 8.5 vol. % and then up to 25 vol. % **B** by 5.5 min. The concentration of **B** was held at 25 vol. % for 2 min and then decreased to the starting conditions over the next 0.5 min. The column was then equilibrated for 2 min before the next injection. 10 mm ammonium formate in methanol was used as make‐up solvent, with a flow rate of 0.6 mL min^−1^. The diode array detector was set at a sampling rate of 20 Hz and a resolution of 1.2 nm and set to collect data continuously from 250 to 500 nm and UV data at three different separate wavelengths, 210 nm, 254 nm and 320 nm, respectively. The retention time reproducibility of the UHPSFC method was determined from the retention times of vanillin, which was identified in all three lignin samples. The obtained reproducibility is shown in Table S79. The HESI source was operated in negative ionization mode with a source temperature of 275 °C, a capillary temperature of 275 °C, a capillary voltage of 3.0 kV, a sheath gas flow of 80 arbitrary units (AU) and an auxiliary gas flow of 20 AU. The screened mass range was from *m*/*z* 80 to *m*/*z* 1500. A MS full‐scan was performed for each sample with a MS resolution of 60 000. For each sample, six data‐dependent neutral loss MS^3^ experiments were performed, screening the three most intense peaks in each MS scan for a neutral loss in the MS^2^ stage of either a methyl radical (CH_3_), water (H_2_O), carbon dioxide (CO_2_), formaldehyde (CH_2_O), formic acid (CH_2_O_2_), or formaldehyde plus water (CH_2_O+H_2_O). For the depolymerized Kraft lignin sample, the data‐dependent neutral loss MS^3^ experiment screening for formaldehyde was not performed. If a neutral loss was detected, a MS^3^ experiment was performed automatically. The MS resolution was set at all MS^*n*^ stages to 30 000.

### Software

The SFC system was operated with MassLynx 4.1 software (Waters) and the MS system with Xcalibur 2.2 (Thermo Fisher Scientific). The open‐source software MZmine2 and Xcalibur 2.2 were used for data evaluation. MATLAB (Mathworks, Natick, MA, USA) and a Classification toolbox for MATLAB (Milano Chemometrics and QSAR Research Group, University of Milan, Milan, Italy) were used for creating the PCA–QDA classification model. ChemDraw software (PerkinElmer Informatics, Waltham, MA, USA) was used to obtain compound names for proposed chemical structures.

### Peak list creation and preselection criteria

A peak list was created for each sample in MZmine 2. The MZmine 2 workflow is shown in Table S80. All *m*/*z* values without a determined chemical formula, a number of carbon atoms below six, or without an oxygen atom were excluded.

### Classification models

An estimated list including in total 172 lignin‐related phenolic compounds was prepared based on previous reports (Table S81).^21^, ^22^, ^23^, ^24^, ^25^, ^26^, ^30^, ^31^ The estimated list included 43 monomers, 58 dimers, 40 trimers, 16 tetramers, 6 pentamers, and 7 hexamers, alongside their exact mass, chemical formula, RDB, type of lignin‐related phenolic compound, a compound label, a literature reference for all Los, and the name of the included LMs. The list does not include lignin–carbohydrate complexes or lignin‐related phenolic compounds including sulfur. KMD–PCA–QDA classification models were created for lignin monomers, dimers, and trimers. Autoscaling (including mean centering and scaling to unit variance) was used for data preprocessing. Each KMD–PCA–QDA classification model was created by using eight variables; the number of C atoms, the number of H atoms, the number of O atoms, and the KMDs calculated by using the base units corresponding to five functional groups typical for lignin‐related phenolic compounds [phenol (C_6_H_5_O), methoxy/primary alcohol (CH_3_O), carboxylic acid (CHO_2_), aldehyde (CHO), and secondary alcohol (CH_2_O)]. The used variable values are given in Table S82. Venetian blinds cross validation using ten cross‐validation groups was used for model validation. For each model, two classes were defined. For example, for the lignin trimer classification model, class one indicates “trimer”, whereas class two indicates “no trimer”.

### Validation of classified *m*/*z* values

For all classified *m*/*z* values, the RDB equivalent, the mass difference between detected and theoretical exact masses, and the ratio between detected and theoretical ^13^C peak intensities were determined. All *m*/*z* values with a RDB equivalent lower than needed for the classified class (e.g., for LMs, the RDB equivalent should be 4 or higher), a mass difference of higher than ±2 mDa between detected and theoretical chemical formula, a nonmatching isotope pattern, or a ratio between the detected theoretical ^13^C isotope peak intensities higher than ±15 % were removed from the peak list. For classified LMs, the obtained retention times were also compared with retention times obtained in a previous study by our group group.^19^ The *m*/*z* values matching all criteria were compared with the estimated list (Table S81). Any *m*/*z* values detected in the solvent blank samples were excluded. Furthermore, possible in‐source fragmentation and obtained MS^2^ and MS^3^ data were investigated and, if available, compared with previously reported MS fragmentation patterns. All validated *m*/*z* values were also investigated if they were possible fragments or adducts of other *m*/*z* values eluting at the same retention time.

The identification confidence for each identified phenolic compound was determined according to the method reported by Schymanski et al.^32^ In their introduced system, identification confidence level 1 is the best identification confidence level that can be achieved. Level 1 (*confirmed structure*) can only be reached by comparison of MS data, MS^*n*^ data, and retention time with a reference standard. At Level 2 (*probable structure*) identification is based on MS data and MS^*n*^ data in combination with either a library spectrum match or diagnostic evidence, like, for example, the observation of a typical fragmentation pattern in MS^*n*^ experiments. Level 3 (*tentative candidate*) is achieved if a part of the structure or a compound class is known based on MS data, MS^*n*^ data, and other experimental data. At level 4 (*unequivocal molecular formula*), the identification is based on a determined chemical formula based on an obtained exact mass and matching isotope patterns, whereas at the lowest identification confidence level, level 5 (*exact mass*), the identification is only based on an obtained exact mass.

In this study, an identification confidence level 1 was given if the detected compound could be confirmed by a reference standard. For tentative lignin monomers, an identification confidence level 2 was given if the mass difference, the ^13^C ratio, and the RDB equivalent were within the acceptable ranges and if MS^2^ or MS^3^ data were obtained. The same criteria were used for an identification confidence level 2 for lignin dimers or higher. However, in the MS^2^ or MS^3^ data, a fragment with a loss of a benzene ring must have been detected; otherwise identification confidence level 3 was given. If no MS^2^ or MS^3^ data were obtained, but the mass difference, the ^13^C ratio, and the RDB equivalent were according to the selected ranges, an identification confidence level 2 was given. PubChem was used to search for possible structures of identified compounds with an identification confidence level of 2 or lower.^33^

## Conflict of interest

*The authors declare no conflict of interest*.

## Supporting information

As a service to our authors and readers, this journal provides supporting information supplied by the authors. Such materials are peer reviewed and may be re‐organized for online delivery, but are not copy‐edited or typeset. Technical support issues arising from supporting information (other than missing files) should be addressed to the authors.

Supplementary
