# Supplementary material for: Nontargeted Analysis Strategy for the Identification of Phenolic Compounds in Complex Technical Lignin Samples
Source: ChemSusChem. 2020 Jul 2;13(17):4605–12. doi: 10.1002/cssc.202000951 (PMC7540015; doi:10.1002/cssc.202000951)
Supplement: Supplementary file 1 — Supplementary [file CSSC-13-4605-s001.pdf]

ChemSusChem

Supporting Information

Nontargeted Analysis Strategy for the Identification of Phenolic Compounds in Complex Technical Lignin Samples

Jens Prothmann,[a] Kena Li,[b] Christian Hulteberg,[b] Peter Spégel,[a] Margareta Sandahl,[a] and Charlotta Turner*[a]

Contents:

Table S1. Number of used components, error rates, cross validation error rates, accuracies and number of false negatives of the four created KMD-PCA-QDA classification models.

Table S2. Identified phenolic compounds in the Lignoboost Kraft lignin sample including the detected $[M-H]^-$, determined chemical formulas, classified class, obtained mass difference, obtained ^{13}C ratio (°: not possible to determine due to overlapping peaks in MS spectrum), ring double bound (RDB) equivalent, retention times (RTs), identified fragments by ESI in-source fragmentation, obtained MS^n data (*: MS^n of identified fragment), identified hits of $[M-H]^-$ on the suspect list (SL) (with 1: suspect list; 2: Prothmann et al.[26], 3: Kiyota et al.[31], 4: Jarrell et al.[25], 5: Banoub et al.[21], 6: Huis et al.[24]), suggested compounds and obtained identification level according to Schymanski et al.[32].

Table S3. Identified phenolic compounds in the Lignosulphonate lignin sample including the detected $[M-H]^-$, determined chemical formulas, compound label, classified class, obtained mass difference, obtained ^{13}C ratio (°: not possible to determine due to overlapping peaks in MS spectrum), ring double bound (RDB) equivalent, retention times (RTs), identified fragments by ESI in-source fragmentation, obtained MS^n spectra (*: MS^n spectra obtained from identified fragment), identified hits of $[M-H]^-$ on the suspect list (SL) (with 1: suspect list; 2: Prothmann et al.[26], 3: Kiyota et al.[31], 4: Jarrell et al.[25]), suggested compounds and obtained identification level according to Schymanski et al.[32].

Table S4. Identified phenolic compounds in the depolymerised Kraft lignin sample including the detected $[M-H]^-$, determined chemical formulas, compound label, classified class, obtained mass difference, obtained ^{13}C ratio (°: not possible to determine due to overlapping peaks in MS spectrum), ring double bound (RDB) equivalent, retention times (RTs), identified fragments by ESI in-source fragmentation, obtained MS^n spectra (*: MS^n of identified fragment), identified hits of $[M-H]^-$ on the suspect list (SL) (with 1: suspect list; 2: Prothmann et al.[26], 4: Jarrell et al.[25], 5: Banoub et al.[21], 7: Albishi et al.[30]), suggested compounds and obtained identification level according to Schymanski et al.[32.]

Table S5. MS^3 fragmentation of m/z 151.0403 detected in the Lignoboost Kraft lignin sample at retention time 1.28 min.

Table S6. MS^3 fragmentation of m/z 177.0558 detected in the Lignoboost Kraft lignin sample at retention time 1.75 min.

Table S7. MS^3 fragmentation of m/z 181.0506 detected in the Lignoboost Kraft lignin sample at retention time 1.98 min.

Table S8. MS^3 fragmentation of m/z 231.0661 detected in the Lignoboost Kraft lignin sample at retention time 4.94 min.

Table S9. MS^2 fragmentation of m/z 245.0817 detected in the Lignoboost Kraft lignin sample at retention time 4.62 min.

Table S10. MS^3 fragmentation of m/z 259.0972 detected in the Lignoboost Kraft lignin sample at retention time 4.45 min.

Table S11. MS^3 fragmentation of m/z 269.0818 detected in the Lignoboost Kraft lignin sample at retention time 3.19 min.

Table S12. MS^3 fragmentation of m/z 273.0764 detected in the Lignoboost Kraft lignin sample at retention time 3.26 min.

Table S13. MS^3 fragmentation of m/z 273.0764 detected in the Lignoboost Kraft lignin sample at retention time 3.53 min.

Table S14. MS^3 fragmentation of m/z 273.1127 detected in the Lignoboost Kraft lignin sample at retention time 4.36 min.

Table S15. MS^3 fragmentation of m/z 273.1127 detected in the Lignoboost Kraft lignin sample at retention time 4.19 min.

Table S16. MS^3 fragmentation of m/z 287.0920 detected in the Lignoboost Kraft lignin sample at retention time 3.44 min. Identified as a fragment of m/z 317.1026.

Table S17. MS^3 fragmentation of m/z 299.0921 detected in the Lignoboost Kraft lignin sample at retention time 3.75 min.

Table S18. MS^3 fragmentation of m/z 301.0716 detected in the Lignoboost Kraft lignin sample at retention time 1.29 min.

Table S19. MS^3 fragmentation of m/z 301.0713 detected in the Lignoboost Kraft lignin sample at retention time 4.29 min.

Table S20. MS^3 fragmentation of m/z 301.1079 detected in the Lignoboost Kraft lignin sample at retention time 3.07 min. Identified as a fragment of m/z 331.1182.

Table S21. MS^3 fragmentation of m/z 303.0871 detected in the Lignoboost Kraft lignin sample at retention time 4.11 min.

Table S22. MS^3 fragmentation of m/z 313.1075 detected in the Lignoboost Kraft lignin sample at retention time 3.65 min.

Table S23. MS^3 fragmentation of m/z 313.1075 detected in the Lignoboost Kraft lignin sample at retention time 5.46 min.

Table S24. MS^3 fragmentation of m/z 315.0869 detected in the Lignoboost Kraft lignin sample at retention time 4.01 min.

Table S25. MS^3 fragmentation of m/z 315.1233 detected in the Lignoboost Kraft lignin sample at retention time 3.11 min.

Table S26. MS^3 fragmentation of m/z 315.1233 detected in the Lignoboost Kraft lignin sample at retention time 5.99 min.

Table S27. MS^2 fragmentation of m/z 325.1075 detected in the Lignoboost Kraft lignin sample at retention time 4.33 min.

Table S28. MS^3 fragmentation of m/z 327.1232 detected in the Lignoboost Kraft lignin sample at retention time 3.53 min.

Table S29. MS^3 fragmentation of m/z 329.1025 detected in the Lignoboost Kraft lignin sample at retention time 4.39 min.

Table S30. MS^3 fragmentation of m/z 329.1389 detected in the Lignoboost Kraft lignin sample at retention time 4.76 min.

Table S31. MS^3 fragmentation of m/z 337.1076 detected in the Lignoboost Kraft lignin sample at retention time 5.00 min. Identified as a fragment of m/z 367.1182.

Table S32. MS^3 fragmentation of m/z 343.1181 detected in the Lignoboost Kraft lignin sample at retention time 4.21 min.

Table S33. MS^3 fragmentation of m/z 343.1181 detected in the Lignoboost Kraft lignin sample at retention time 4.91 min.

Table S34. MS^3 fragmentation of m/z 345.1338 detected in the Lignoboost Kraft lignin sample at retention time 4.60 min.

Table S35. MS^2 fragmentation of m/z 349.1075 detected in the Lignoboost Kraft lignin sample at retention time 3.94 min.

Table S36. MS^3 fragmentation of m/z 351.1232 detected in the Lignoboost Kraft lignin sample at retention time 4.26 min. Identified as a fragment of m/z 381.1339.

Table S37. MS^3 fragmentation of m/z 355.1180 detected in the Lignoboost Kraft lignin sample at retention time 4.03 min. Identified as a fragment of m/z 401.1234.

Table S38. MS^3 fragmentation of m/z 357.1337 detected in the Lignoboost Kraft lignin sample at retention time 3.46 min.

Table S39. MS^3 fragmentation of m/z 357.1338 detected in the Lignoboost Kraft lignin sample at retention time 3.69 min.

Table S40. MS^3 fragmentation of m/z 361.1287 detected in the Lignoboost Kraft lignin sample at retention time 5.57 min.

Table S41. MS^3 fragmentation of m/z 361.1650 detected in the Lignoboost Kraft lignin sample at retention time 5.05 min.

Table S42. MS^3 fragmentation of m/z 419.1494 detected in the Lignoboost Kraft lignin sample at retention time 5.34 min.

Table S43. MS^3 fragmentation of m/z 491.1704 detected in the Lignoboost Kraft lignin sample at retention time 5.71 min.

Table S44. MS^3 fragmentation of m/z 509.2176 detected in the Lignoboost Kraft lignin sample at retention time 6.36 min.

Table S45. MS^2 fragmentation of m/z 163.0399 detected in the Lignosulphonate lignin sample at retention time 2.35 min.

Table S46. MS^3 fragmentation of m/z 195.0659 detected in the Lignosulphonate lignin sample at retention time 1.82 min.

Table S47. MS^3 fragmentation of m/z 287.0917 detected in the Lignosulphonate lignin sample at retention time 3.44 min.

Table S48. MS^3 fragmentation of m/z 343.1538 detected in the Lignosulphonate lignin sample at retention time 3.44 min. Identified as fragment of m/z 389.1593.

Table S49. MS^3 fragmentation of m/z 371.1122 detected in the Lignosulphonate lignin sample at retention time 3.96 min.

Table S50. MS^3 fragmentation of m/z 373.1277 detected in the Lignosulphonate lignin sample at retention time 4.46 min. Identified as fragment of m/z 403.1388.

Table S51. MS^2 fragmentation of m/z 403.1384 detected in the Lignosulphonate lignin sample at retention time 4.46 min.

Table S52. MS^3 fragmentation of m/z 357.1330 detected in the Lignosulphonate lignin sample at retention time 4.46 min. Identified as fragment of m/z 403.1388.

Table S53. MS^2 fragmentation of m/z 135.0450 detected in the depolymerised Kraft lignin sample at retention time 2.51 min.

Table S54. MS^2 fragmentation of m/z 137.0243 detected in the depolymerised Kraft lignin sample at retention time 4.05 min.

Table S55. MS^3 fragmentation of m/z 151.0399 detected in the depolymerised Kraft lignin sample at retention time 1.25 min.

Table S56. MS^2 fragmentation of m/z 165.0554 detected in the depolymerised Kraft lignin sample at retention time 2.23 min.

Table S57. MS^3 fragmentation of m/z 181.0503 detected in the depolymerised Kraft lignin sample at retention time 1.63 min.

Table S58. MS^3 fragmentation of m/z 181.0503 detected in the depolymerised Kraft lignin sample at retention time 3.68 min.

Table S59. MS^3 fragmentation of m/z 271.0604 detected in the depolymerised Kraft lignin sample at retention time 4.53 min.

Table S60. MS^3 fragmentation of m/z 273.0759 detected in the depolymerised Kraft lignin sample at retention time 3.28 min.

Table S61. MS^3 fragmentation of m/z 287.0915 detected in the depolymerised Kraft lignin sample at retention time 2.94 min.

Table S62. MS^3 fragmentation of m/z 301.0706 detected in the depolymerised Kraft lignin sample at retention time 3.93 min.

Table S63. MS^3 fragmentation of m/z 301.0707 detected in the depolymerised Kraft lignin sample at retention time 4.25 min.

Table S64. MS^3 fragmentation of m/z 301.1071 detected in the depolymerised Kraft lignin sample at retention time 2.99 min.

Table S65. MS^3 fragmentation of m/z 313.1436 detected in the depolymerised Kraft lignin sample at retention time 5.18 min.

Table S66. MS^3 fragmentation of m/z 313.1436 detected in the depolymerised Kraft lignin sample at retention time 5.75 min.

Table S67. MS^3 fragmentation of m/z 315.0861 detected in the depolymerised Kraft lignin sample at retention time 4.00 min.

Table S68. MS^3 fragmentation of m/z 315.1227 detected in the depolymerised Kraft lignin sample at retention time 3.13 min.

Table S69. MS^2fragmentation of m/z 317.1022 detected in the depolymerised Kraft lignin sample at retention time 4.50 min.

Table S70. MS^2 fragmentation of m/z 329.1017 detected in the depolymerised Kraft lignin sample at retention time 3.80 min.

Table S71. MS^2 fragmentation of m/z 329.1016 detected in the depolymerised Kraft lignin sample at retention time 3.87 min.

Table S72. MS^2 fragmentation of m/z 329.1019 detected in the depolymerised Kraft lignin sample at retention time 4.16 min.

Table S73. MS^3 fragmentation of m/z 329.1382 detected in the depolymerised Kraft lignin sample at retention time 6.18 min.

Table S74. MS^3 fragmentation of m/z 331.1174 detected in the depolymerised Kraft lignin sample at retention time 4.69 min.

Table S75. MS^3 fragmentation of m/z 345.1333 detected in the depolymerised Kraft lignin sample at retention time 4.61 min. Identified as fragment of 419.1700.

Table S76. MS^3 fragmentation of m/z 345.1333 detected in the depolymerised Kraft lignin sample at retention time 6.86 min.

Table S77. MS^3 fragmentation of m/z 347.1123 detected in the depolymerised Kraft lignin sample at retention time 5.29 min.

Table S78. MS^3 fragmentation of m/z 359.1491 detected in the depolymerised Kraft lignin sample at retention time 4.47 min.

Table S79. Reproducibility of the retention time of vanillin.
Table S80. Peak list creation workflow using MZmine 2.

Table S81. Suspect list of lignin-related phenolic compounds identified in literature including exact mass of neutral compound, exact mass of deprotonated compounds, exact mass of protonated compound, chemical formula of neutral compound, ring double bond equivalent (RDB), type pf lignin compound, compound label, reference and compound name.

Table S82. Variables used for the four KMD-PCA-QDA classification models including the number of C-atoms (#C), number of H-atoms (#H), the number of O-atoms (#O) and the calculated KMDs for phenol (C_6H_5O), methoxy/primary alcohol (CH_3O), carboxylic acid (CHO_2), aldehyde (CHO) and secondary alcohol (CH_2O).

Figure S1. Scores plot of the KMD-PCA-QDA classification model for lignin trimers showing principal component (PC) 1 and 2.

Figure S2. Scores plot of the KMD-PCA-QDA classification model for lignin trimers showing principal component (PC) 2 and 3.

Figure S3. Loading plot of the KMD-PCA-QDA classification model for lignin trimers showing latent variable (LV) 1 and 2.

Figure S4. Loading plot of the KMD-PCA-QDA classification model for lignin trimers showing latent variable (LV) 2 and 3.

Figure S5. Scores plot of the KMD-PCA-QDA classification model for lignin trimers showing principal component (PC) 1 and 3 including the 133 m/z values from the Lignosulphonate lignin sample (green stars).

Figure S6. Scores plot of the KMD-PCA-QDA classification model for lignin trimers showing principal component (PC) 1 and 3 including the 112 m/z values from the depolymerised Kraft lignin sample (green stars).

Table S1. Number of used components, error rates, cross validation error rates, accuracies and number of false negatives of the four created KMD-PCA-QDA classification models.

Classification Model	Number of components	Error rate	Cross validation error rate	Accuracy	Number of false negatives
Monomers	2	0.01	0.01	0.99	1 out of 43
Dimers	3	0.03	0.05	0.98	4 out of 58
Trimers	3	0.03	0.04	0.99	2 out of 40
Tetramers	4	0.06	0.09	0.99	2 out of 16

Table S2. Identified phenolic compounds in the Lignoboost Kraft lignin sample including the detected $[M-H]^-$, determined chemical formulas, classified class, obtained mass difference, obtained ^{13}C ratio (°: not possible to determine due to overlapping peaks in MS spectrum), ring double bound (RDB) equivalent, retention times (RTs), identified fragments by ESI in-source fragmentation, obtained MS^n data (*: MS^n of identified fragment), identified hits of $[M-H]^-$ on the suspect list (SL) (with 1: suspect list; 2: Prothmann et al.[26], 3: Kiyota et al.[31], 4: Jarrell et al.[25], 5: Banoub et al.[21], 6: Huis et al.[24]), suggested compounds and obtained identification level according to Schymanski et al.[32].

$[M-H]^-$	Chemical formula (neutral)	Classified Class	Mass diff. (mDa)	^{13}C ratio	RDB	RTs (min)	In-source fragmentation	MS^n	SL hit	Suggested compounds	Identification confidence level
135.0454	$C_8H_8O_2$	Monomer	0.3	1.04	5.0	2.54			1	4-hydroxyacetophenone	1
137.0246	$C_7H_6O_3$	Monomer	0.2	1.08	5.0	3.91			1	3-hydroxybenzoic acid	3
151.0402	$C_8H_8O_3$	Monomer	0.2	1.00	5.0	1.28	136	MS^3	1	Vanillin	1
165.0558	$C_9H_{10}O_3$	Monomer	0.1	1.00	5.0	1.28	150		1	Acetovanillone	1
				1.05		4.76				3,4’-dihydroxy propiophenone	3
167.0350	$C_8H_8O_4$	Monomer	0.0	0.97	5.0	3.31	152, 149, 123		1	Vanillic acid	1
167.0714	$C_9H_{12}O_3$	Monomer	0.1	1.13	4.0	4.50				4-(1-hydroxyethyl)-2-methoxyphenol	3
				0.92		5.05				4-(3-hydroxypropyl) benzene-1,2-diol	3
177.0558	$C_{10}H_{10}O_3$	Monomer	0.1	0.97	6.0	1.75	162	MS^3	1	Coniferyl aldehyde	1
				1.13		4.05				(E)-4-(3-hydroxybuta-1,3-dien-1-yl)benzene-1,2-diol	3
181.0506	$C_9H_{10}O_4$	Monomer	0.0	1.15	5.0	1.98	166	MS^3	1	Homovanillic acid	2
183.0452	$C_{12}H_8O_2$	Monomer	0.1	0.87	9.0	3.19					3
187.0765	$C_{12}H_{12}O_2$	Monomer	0.1	1.00	7.0	3.23				(E)-(5-(prop-1-en-1-yl)benzofuran-3-yl)methanol	3
195.0662	$C_{10}H_{12}O_4$	Monomer	-0.1	1.00	5.0	1.84			1	1-hydroxy-1-(4-hydroxy-3-methoxyphenyl) propan-2-one	3
				1.00		2.84				Homosyringaldehyde	3
				0.89		3.01				3,5-dimethoxy-phenylacetic acid	3

203.0713	$C_{12}H_{12}O_3$	Monomer	0.0	0.95	7.0	2.56				(7-methoxy-5-vinylbenzofuran-3-yl)methanol	3
				1.00		3.72				(Z)-1-(3-(hydroxymethyl)benzofuran-5-yl)prop-1-en-1-ol	3
211.0611	$C_{10}H_{12}O_5$	Monomer	0.1	0.93	5.0	1.85					3
				0.98		1.90					3
				0.88		3.68				2,3-dihydroxy-1-(4-hydroxy-3-methoxyphenyl) propan-1-one	3
227.0381	$C_{10}H_{12}O_4S$	Monomer	-0.2	1.08		2.76				(Z)-1-(3-methoxyphenyl) prop-1-ene-1-sulfonic acid	3
231.0661	$C_{13}H_{12}O_4$	Monomer	-0.2	1.08	8.0	4.94	216	MS^3		(E)-3-(3-(hydroxymethyl)-7-methoxybenzofuran-5-yl)acrylaldehyde	2
233.0275	$C_{12}H_{10}O_3S$	Dimer	-0.3	0.95		1.95					3
				1.00		2.11					3
241.0504	$C_{14}H_{10}O_4$	Dimer	-0.2	0.95	10.0	5.15					3
241.0867	$C_{15}H_{14}O_3$	Dimer	-0.3	1.00	9.0	4.20					3
245.0816	$C_{14}H_{14}O_4$	Dimer	-0.3	1.03	8.0	0.57			2		3
				1.13		4.62		MS^2			2
253.0869	$C_{16}H_{14}O_3$	Dimer	-0.1	0.89	10.0	3.51					3
257.0816	$C_{15}H_{14}O_4$	Dimer	-0.3	0.99	9.0	2.84			2		3
				0.92		5.15					3
259.0610	$C_{14}H_{12}O_5$	Dimer	-0.2	°	9.0	5.12					3
259.0973	$C_{15}H_{16}O_4$	Dimer	-0.3	0.94	8.0	3.01	229		2		3
				1.03		4.24					3
				1.03		4.45	229	MS^3			3
269.0817	$C_{16}H_{14}O_4$	Dimer	-0.2	1.12	10.0	2.08	254		2		3
				°		3.19	254	MS^3			3

				0.93		4.46	254				3
271.0611	$C_{15}H_{12}O_5$	Dimer	-0.1	°	10.0	3.19					3
				°		4.58					3
271.0973	$C_{16}H_{16}O_4$	Dimer	-0.3	0.89	9.0	3.91			2		3
				1.00		4.07					3
273.0764	$C_{15}H_{14}O_5$	Dimer	-0.4	1.00	9.0	3.26	258	MS^3	2		3
				1.00		3.53		MS^3			3
273.1129	$C_{16}H_{18}O_4$	Dimer	-0.3	0.99	8.0	2.94			2		3
				1.00		4.19		MS^3			3
				1.00		4.36	243, 229	MS^3			2
285.1129	$C_{17}H_{18}O_4$	Dimer	-0.3	0.86	9.0	3.53					3
287.0920	$C_{16}H_{16}O_5$	Dimer	-0.5	1.00	9.0	3.13	272, 259		2		3
				1.00		5.08					3
				0.87		5.15					3
287.1284	$C_{17}H_{20}O_4$	Dimer	-0.5	0.88	8.0	4.14					3
				0.91		4.29					3
				1.10		5.57					3
289.0715	$C_{15}H_{14}O_6$	Dimer	-0.2	0.96	9.0	5.44					3
295.0974	$C_{18}H_{16}O_4$	Dimer	-0.2	0.90	11.0	4.66					3
297.1128	$C_{18}H_{18}O_4$	Dimer	-0.4	0.88	10.0	3.17			3		3
				0.91		4.23					3
299.0921	$C_{17}H_{16}O_5$	Dimer	-0.4	0.94	10.0	2.76			2		3
				1.05		3.75	284, 269	MS^3			3
301.0714	$C_{16}H_{14}O_6$	Dimer	-0.3	1.00	10.0	1.26	286	MS^3	2		3
				0.89		4.29		MS^3			3
301.1077	$C_{17}H_{18}O_5$	Dimer	-0.4	1.00	9.0	2.97	273, 255				3
				1.03		3.28					3

301.1440	$C_{18}H_{22}O_4$	Dimer	-0.5	°	8.0	4.29					3
303.0869	$C_{16}H_{16}O_6$	Dimer	-0.5	1.00	9.0	3.84			4		3
				1.05		4.11		MS^3			2
313.1077	$C_{18}H_{18}O_5$	Dimer	-0.4	1.00	10.0	3.65	298, 283	MS^3	2		3
				1.00		4.68					3
				1.02		5.46		MS^3			2
315.0869	$C_{17}H_{16}O_6$	Dimer	-0.2	1.00	10.0	4.01	252	MS^3			3
				0.93		5.20					3
315.1234	$C_{18}H_{20}O_5$	Dimer	-0.4	1.03	9.0	3.11		MS^3	2		2
				0.91		5.99		MS^3			2
317.1026	$C_{17}H_{18}O_6$	Dimer	-0.4	°	9.0	3.46	287, 273	MS^{3*}			3
				0.97		3.70	287, 273				3
				0.87		4.65	299, 287, 273				3
317.1390	$C_{18}H_{22}O_5$	Dimer	-0.3	0.85	8.0	2.86					3
				1.08		5.48					3
319.1183	$C_{17}H_{20}O_6$	Dimer	-0.4	1.00	8.0	2.86					3
				0.87		5.01					3
				0.92		5.15					3
321.1128	$C_{20}H_{18}O_4$	Dimer	-0.4	0.90	12.0	4.30			2		3
				1.00		4.87					3
323.0921	$C_{19}H_{16}O_5$	Dimer	-0.4	1.05	12.0	3.85					3
				0.95		4.72					3
				0.92		6.41					3
325.1077	$C_{19}H_{18}O_5$	Dimer	-0.4	0.92	11.0	4.33			5		3
327.0870	$C_{18}H_{16}O_6$	Dimer	-0.4	1.17	11.0	3.28					3
				°		4.16					3
				0.87		4.68					3

327.1234	$C_{19}H_{20}O_5$	Dimer	-0.4	0.98	10.0	3.47			3		3
				0.96		3.53		MS^3			3
				0.93		3.95					3
329.1026	$C_{18}H_{18}O_6$	Dimer	-0.4	0.95	10.0	1.28	315, 301, 286		5		3
				1.00		3.68					3
				0.90		3.81	299, 284, 269				3
				1.02		3.89	299, 284, 269				3
				1.07		4.39	285	MS^3			2
329.1390	$C_{19}H_{22}O_5$	Dimer	-0.4	1.00	9.0	2.98					3
				1.00		3.26					3
				1.03		4.76		MS^3			3
331.0819	$C_{17}H_{16}O_7$	Dimer	-0.4	0.88	10.0	4.72	287, 285				3
331.1006	$C_{18}H_{20}O_4S$	Dimer	-0.3	°		4.62					3
				0.94		4.68					3
331.1182	$C_{18}H_{20}O_6$	Dimer	-0.5	1.05	9.0	3.07	301	MS^{3*}	5		2
				1.00		3.65	314, 315, 298, 283				3
				0.89		4.69	313, 301, 287, 285				3
337.1076	$C_{20}H_{18}O_5$	Dimer	-0.5	1.10	12.0	4.73					3
				0.92		5.70					3
				0.85		5.84					3
339.0871	$C_{19}H_{16}O_6$	Dimer	-0.3	0.90	12.0	5.01			5		3
339.1234	$C_{20}H_{20}O_5$	Dimer	-0.4	1.05	11.0	3.76			5		3
				1.04		4.04					3
341.1026	$C_{19}H_{18}O_6$	Dimer	-0.4	0.92	11.0	3.19	297		5		3
				1.00		3.47					3
				1.11		4.17					3
				1.03		4.46					3

				0.93		4.78					3
				0.91		4.97	313				3
				0.92		6.16					3
343.1181	$C_{19}H_{20}O_6$	Dimer	-0.6	0.86	10.0	3.70	315, 313		5		3
				1.06		3.92					3
				1.02		4.01					3
				1.00		4.11					3
				1.10		4.21		MS^3			2
				0.91		4.85	315				3
				1.00		4.91		MS^3			2
343.1548	$C_{20}H_{24}O_5$	Dimer	-0.3	0.95	9.0	3.00					3
				1.03		3.53					3
345.1338	$C_{19}H_{22}O_6$	Dimer	-0.5	1.04	9.0	4.60		MS^3			2
345.1702	$C_{20}H_{26}O_5$	Dimer	-0.5	0.92	8.0	4.05					3
347.0953	$C_{18}H_{20}O_5S$	Dimer	-0.5	1.00		3.29					3
				1.02		3.41					3
				1.01		3.53					3
347.1133	$C_{18}H_{20}O_7$	Dimer	-0.3	1.00	9.0	5.10	329, 299				3
				0.89		5.30	329, 299				3
				0.92		5.44					3
347.1495	$C_{19}H_{24}O_6$	Dimer	-0.5	1.02	8.0	5.07	329, 312, 287				3
				1.02		6.05	299				3
349.0746	$C_{17}H_{18}O_6S$	Dimer	-0.5	0.97		3.88					3
349.1076	$C_{21}H_{18}O_5$	Dimer	-0.5	0.97	13.0	3.94		MS^2	2		3
				1.00		4.29					3
353.1389	$C_{21}H_{22}O_5$	Dimer	-0.5	0.88	11.0	3.70			2		3
				1.00		4.23					3

				1.10		4.55					3
355.1181	$C_{20}H_{20}O_6$	Dimer	-0.6	0.92	11.0	4.27					3
				1.02		4.56					3
				0.85		5.83					3
357.1337	$C_{20}H_{22}O_6$	Dimer	-0.6	1.02	10.0	3.56		MS^3	2		2
				1.05		3.69		MS^3			2
				0.95		3.86					3
				1.07		3.98					3
				1.00		4.08					3
359.1130	$C_{19}H_{20}O_7$	Dimer	-0.6	0.91	10.0	4.16					3
				°		4.88					3
359.1494	$C_{20}H_{24}O_6$	Dimer	-0.6	0.94	9.0	3.79	329, 299				3
				1.00		4.66					3
				0.94		5.44	329				3
361.0926	$C_{18}H_{18}O_8$	Dimer	-0.3	1.00	10.0	1.99					3
				°		5.17					3
361.1076	$C_{22}H_{18}O_5$	Dimer	-0.5	°	14.0	5.64					3
361.1288	$C_{19}H_{22}O_7$	Dimer	-0.5	1.06	9.0	5.57		MS^3			2
361.1650	$C_{20}H_{26}O_6$	Dimer	-0.6	1.00	8.0	4.30			2		3
				1.06		5.05		MS^3			2
363.1230	$C_{22}H_{20}O_5$	Dimer	-0.8	0.96	13.0	3.82					3
				1.01		4.16					3
				1.00		4.76					3
				1.09		4.92					3
				1.02		5.28					3
365.1024	$C_{21}H_{18}O_6$	Dimer	-0.6	0.88	13.0	3.92					3
				1.05		4.26	332				3

365.1387	$C_{22}H_{22}O_5$	Dimer	-0.7	0.87	12.0	4.01					3
				1.00		4.34					3
				0.94		4.95					3
367.1181	$C_{21}H_{20}O_6$	Dimer	-0.6	1.02	12.0	5.00	337	MS^{3*}	5		3
				1.12		5.80	337				3
369.1338	$C_{21}H_{22}O_6$	Dimer	-0.5	1.02	11.0	4.46	339				3
				0.90		5.24					3
373.1286	$C_{20}H_{22}O_7$	Dimer	-0.7	1.04	10.0	4.46			2		3
				0.96		5.18	343				3
				0.96		5.93	355, 345				3
375.1596	$C_{24}H_{24}O_4$	Trimer	-0.6	1.00	13.0	4.00					3
377.0881	$C_{18}H_{18}O_9$	Dimer	0.3	1.00	10.0	3.35					3
379.1181	$C_{22}H_{20}O_6$	Dimer	-0.6	0.93	13.0	4.71					3
				1.00		5.41					3
				1.00		6.48					3
381.1336	$C_{22}H_{22}O_6$	Dimer	-0.7	1.00	12.0	4.27	351	MS^{3*}			3
				1.00		5.28					3
				0.98		6.29					3
383.1494	$C_{22}H_{24}O_6$	Dimer	-0.6	0.95	11.0	4.24					3
389.1599	$C_{21}H_{26}O_7$	Dimer	-0.7	0.88	9.0	4.37	361				3
				0.86		4.71	359				3
391.1543	$C_{24}H_{24}O_5$	Trimer	-0.8	0.86	13.0	4.17	361				3
393.1336	$C_{23}H_{22}O_6$	Dimer	-0.6	1.00	13.0	4.84			2		3
				1.00		5.80					3
395.1493	$C_{23}H_{24}O_6$	Dimer	-0.7	0.97	12.0	4.75			2		3
				0.96		5.04					3
				0.98		5.47					3

				0.94		5.56					3
				0.92		5.73					3
399.1442	$C_{22}H_{24}O_7$	Dimer	-0.7	0.86	11.0	4.58	381, 369, 351				3
				0.96		4.79	381, 369				3
				0.93		6.06	381				3
401.1235	$C_{21}H_{22}O_8$	Dimer	-0.7	1.02	11.0	4.03	357, 355	MS^{3*}	5		2
				0.94		4.13					3
403.1542	$C_{25}H_{24}O_5$	Trimer	-0.3	1.00	14.0	4.32					3
413.1599	$C_{23}H_{26}O_7$	Dimer	-0.7	1.00	11.0	6.83					3
417.1548	$C_{22}H_{26}O_8$	Dimer	-0.8	0.86	10.0	5.58			3		3
419.1492	$C_{25}H_{24}O_6$	Trimer	-0.8	1.00	14.0	5.34		MS^3	2		2
421.1649	$C_{25}H_{26}O_6$	Trimer	-0.7	0.86	13.0	4.10	403, 391				3
				0.87		4.89					3
423.1806	$C_{25}H_{28}O_6$	Trimer	-0.6	0.87	12.0	3.81	378				3
				0.85		4.05					3
				0.85		5.17					3
				0.86		5.33					3
447.1443	$C_{26}H_{24}O_7$	Trimer	-0.5	0.85	15.0	5.25					3
				0.90		5.50	419, 417				3
451.1754	$C_{26}H_{28}O_7$	Trimer	-0.8	0.93	13.0	4.59	421, 405		2		3
				0.86		5.76	421				3
				0.87		5.84	421				3
461.1598	$C_{27}H_{26}O_7$	Trimer	-0.8	0.88	15.0	5.40					3
				1.06		5.93					3
				0.89		6.00					3
463.1756	$C_{27}H_{28}O_7$	Trimer	-0.6	0.92	14.0	4.75	433		2		3
477.1912	$C_{28}H_{30}O_7$	Trimer	-0.7	0.88	14.0	6.23					3

479.1705	$C_{27}H_{28}O_8$	Trimer	-0.5	1.00	14.0	4.81	449, 433		2		3
				0.95		4.87	461, 449, 435, 433				3
				0.96		4.97	461, 449, 433, 431				3
483.2018	$C_{27}H_{32}O_8$	Trimer	-0.6	0.98	12.0	5.82	453, 435		2		3
491.1705	$C_{28}H_{28}O_8$	Trimer	-0.6	0.92	15.0	5.63	473		2		3
				0.95		5.71		MS^3			2
505.1860	$C_{29}H_{30}O_8$	Trimer	-0.8	1.00	15.0	5.12	475, 459				3
				0.89		5.41					3
				0.95		5.46					3
507.1656	$C_{28}H_{28}O_9$	Trimer	-0.4	0.90	15.0	5.24	479, 461		2		3
509.1812	$C_{28}H_{30}O_9$	Trimer	-0.5	0.92	14.0	7.05	491, 479, 463				3
509.2174	$C_{29}H_{34}O_8$	Trimer	-0.7	0.93	13.0	5.61	479		2		3
				1.00		6.36		MS^3			2
511.1967	$C_{28}H_{32}O_9$	Trimer	-0.3	0.96	13.0	5.47	493, 481, 463				3
				0.97		6.10	493, 481, 463				3
521.1810	$C_{29}H_{30}O_9$	Trimer	-0.9	0.85	15.0	5.08	503, 493, 491, 477, 475		5		3
523.1967	$C_{29}H_{32}O_9$	Trimer	-0.6	0.91	14.0	5.70	505				3
557.2385	$C_{30}H_{38}O_{10}$	Trimer	-0.7	0.90	12.0	6.51	509		6		3
619.1968	$C_{37}H_{32}O_9$	Tetramer	-0.5	0.96	22.0	6.70					3
621.2121	$C_{37}H_{34}O_9$	Tetramer	-0.9	1.00	21.0	6.15					3
627.2228	$C_{36}H_{36}O_{10}$	Tetramer	-0.7	1.00	19.0	6.31	597, 599		2		3
				0.89		6.39	597, 599				3
631.2541	$C_{36}H_{40}O_{10}$	Tetramer	-0.7	0.98	17.0	7.79	599		2		3

Table S3. Identified phenolic compounds in the Lignosulphonate lignin sample including the detected [M-H]$^-$, determined chemical formulas, compound label, classified class, obtained mass difference, obtained ^{13}C ratio (°: not possible to determine due to overlapping peaks in MS spectrum), ring double bound (RDB) equivalent, retention times (RTs), identified fragments by ESI in-source fragmentation, obtained MS^n spectra (*: MS^n spectra obtained from identified fragment), identified hits of [M-H]$^-$ on the suspect list (SL) (with 1: suspect list; 2: Prothmann et al.[26], 3: Kiyota et al.[31], 4: Jarrell et al.[25]), suggested compounds and obtained identification level according to Schymanski et al.[32].

[M-H]-	Chemical formula (neutral)	Classified class	Mass diff. (mDa)	^{13}C ratio	RDB	RTs (min)	In-source fragmentation	MSn	SL hit	Suggested compounds	Identification confidence level
109.0296	$C_6H_6O_2$	Monomer	0.1	1.04	4.0	2.86			1	Catechol	2
137.0244	$C_7H_6O_3$	Monomer	0.0	1.00	5.0	4.01	93		1	4-hydroxybenzoic acid	1
151.0399	$C_8H_8O_3$	Monomer	0.1	0.93	5.0	1.24	136		1	Vanillin	1
				1.00		2.31				3-methoxybenzoic acid	3
				0.89		3.45				4-hydroxyphenylacetic acid	3
163.0398	$C_9H_8O_3$	Monomer	-0.2	1.00	6.0	2.35		MS^2		3-coumaric acid	2
				1.00		4.10			1	4-coumaric acid	1
165.0555	$C_9H_{10}O_3$	Monomer	-0.2	1.00	5.0	1.14			1	3-(4-hydroxyphenyl) propionic acid	3
167.0347	$C_8H_8O_4$	Monomer	0.3	1.00	5.0	3.28	152		1	Vanillic acid	1
167.0711	$C_9H_{12}O_3$	Monomer	-0.2	0.97	4.0	2.54	123, 121				3
				0.90		2.58	139			Homovanillyl alcohol	3
				1.06		4.49	137			4-(1-hydroxyethyl)-2-methoxyphenol	3
177.0554	$C_{10}H_{10}O_3$	Monomer	-0.3	0.93	6.0	1.75	162		1	Coniferyl aldehyde	1
				1.08		2.27			1	4-methoxycinnamic acid	1
				0.93		2.36	162			3-methoxycinnamic acid	3
				1.05		3.51				(E)-3,4-dihydroxy-benzylideneacetone	3
179.0710	$C_{10}H_{12}O_3$	Monomer	-0.3	0.93	5.0	2.80				3-(4-hydroxy-3-methoxyphenyl)propanal	3
				1.00		3.24	149			4-ethenyl-2,6-dimethoxyphenol	3

181.0503	$C_9H_{10}O_4$	Monomer	-0.3	0.92	5.0	3.24	137			3,5-dimethoxybenzoic acid	3
				0.97		4.42	163, 137			2,4'-dihydroxy-3'-methoxyacetophenone	3
191.0346	$C_{10}H_8O_4$	Monomer	-0.4	0.87	7.0	3.47				7-methoxy-1-benzofuran-3-carboxylic acid	3
191.0709	$C_{11}H_{12}O_3$	Monomer	-0.4	0.98	6.0	3.10				Vanillylidene acetone	3
195.0658	$C_{10}H_{12}O_4$	Monomer	-0.5	1.00	5.0	1.82	180	MS^2	1	1-hydroxy-1-(4-hydroxy-3-methoxyphenyl) propan-2-one	2
199.0607	$C_9H_{12}O_5$	Monomer	-0.5	0.94	4.0	2.52	171				3
				0.91		2.69	171				3
287.0917	$C_{16}H_{16}O_5$	Dimer	-0.8	0.99	9.0	3.44		MS^3	2		2
301.1073	$C_{17}H_{18}O_5$	Dimer	-0.8	0.93	9.0	2.97					3
313.1436	$C_{19}H_{22}O_4$	Dimer	-0.9	0.93	9.0	2.88	269				3
				°		3.85					3
329.1383	$C_{19}H_{22}O_5$	Dimer	-1.1	0.93	9.0	4.57					3
353.1023	$C_{20}H_{18}O_6$	Dimer	-0.7	1.00	12.0	4.37					3
355.1175	$C_{20}H_{20}O_6$	Dimer	-1.2	1.01	11.0	4.04	340				3
				1.04		4.13	337				3
357.1331	$C_{20}H_{22}O_6$	Dimer	-1.2	1.03	10.0	1.14			2,3		3
				1.00		3.27					3
				1.05		3.70					3
359.1488	$C_{20}H_{24}O_6$	Dimer	-1.2	0.95	9.0	5.44					3
371.1126	$C_{20}H_{20}O_7$	Dimer	-1.0	0.97	11.0	3.95		MS^3	4		2
				0.89		4.46					3
				0.87		4.62					3
				0.94		4.98					3
373.1282	$C_{20}H_{22}O_7$	Dimer	-1.4	1.12	10.0	5.88	354		2		3
381.2034	$C_{24}H_{30}O_4$	Trimer	-3.7	0.85	10.0	2.01					3

389.1052	$C_{20}H_{22}O_6S$	Dimer	-1.2	1.00		4.95					3
				0.90		5.05					3
				1.10		5.13					3
				1.00		5.27					3
389.1593	$C_{21}H_{26}O_7$	Dimer	-1.3	0.96	9.0	3.00	343	MS^{3*}			2
389.2145	$C_{23}H_{34}O_3S$	Trimer	-1.1	0.94		2.11					3
403.1388	$C_{21}H_{24}O_8$	Dimer	-1.0	0.86	10.0	3.57	357	MS^{3*}			2

Table S4. Identified phenolic compounds in the depolymerised Kraft lignin sample including the detected $[M-H]^-$, determined chemical formulas, compound label, classified class, obtained mass difference, obtained ^{13}C ratio (°: not possible to determine due to overlapping peaks in MS spectrum), ring double bound (RDB) equivalent, retention times (RTs), identified fragments by ESI in-source fragmentation, obtained MS^n spectra (*: MS^n of identified fragment), identified hits of $[M-H]^-$ on the suspect list (SL) (with 1: suspect list; 2: Prothmann et al.[26], 4: Jarrell et al.[25], 5: Banoub et al.[21], 7: Albishi et al.[30]), suggested compounds and obtained identification level according to Schymanski et al.[32].

[M-H]-	Chemical formula (neutral)	Classified class	Mass diff. (mDa)	^{13}C ratio	RDB	RTs (min)	In-source fragmentation	MSn	SL hit	Suggested compound	Identification confidence level
121.0295	$C_7H_6O_2$	Monomer	0.0	1.10	5.0	2.51			1	4-hydroxybenzaldehyde	1
135.0451	$C_8H_8O_2$	Monomer	0.0	1.04	5.0	2.54		MS^2	1	4-hydroxyacetophenone	1
137.0243	$C_7H_6O_3$	Monomer	-0.1	0.96	5.0	4.05		MS^2	1	4-hydroxybenzoic acid	1
151.0399	$C_8H_8O_3$	Monomer	-0.1	1.00	5.0	1.25	136, 108	MS^3	1	Vanillin	1
165.0554	$C_9H_{10}O_3$	Monomer	-0.3	0.97	5.0	1.25			1	Acetovanillone	1
				1.00		2.23		MS^2		Homovanillin	2
167.0347	$C_8H_8O_4$	Monomer	-0.3	1.03	5.0	1.62	137		1	3,4-dihydroxy-5-methoxybenzaldehyde	3
				1.04		4.08	137			2-(3,4-dihydroxy acetaldehyde	3
175.0761	$C_{11}H_{12}O_2$	Monomer	-0.3	0.97	6.0	2.85				(5-ethylbenzo-furan-3-yl)methanol	3
181.0502	$C_9H_{10}O_4$	Monomer	-0.4	1.06	5.0	1.63	166, 151, 123	MS^3	1	Syringaldehyde	1
				0.95		3.68		MS^3		3,4-dihydroxy-5-methoxy-acetophenone	2
195.0658	$C_{10}H_{12}O_4$	Monomer	-0.5	1.00	5.0	1.63			1	Acetosyringone	1
				1.00		3.22	165			1-(3,4-dihydroxy-5-methoxyphenyl) propan-2-one	3
203.0709	$C_{12}H_{12}O_3$	Monomer	-0.4	1.00	7.0	2.57	188			(7-methoxy-5-vinylbenzofuran -3-yl)methanol	3
233.0270	$C_{12}H_{10}O_3S$	Dimer	-0.8	1.03		2.14	218				3
243.0653	$C_{14}H_{12}O_4$	Dimer	-1.0	1.08	9.0	3.40	228		2		3
				0.93		3.52					3

245.0810	$C_{14}H_{14}O_4$	Dimer	-0.9	0.88	8.0	5.26	230		2		3
257.0447	$C_{14}H_{10}O_5$	Dimer	-0.8	1.00	10.0	3.27	229, 211				3
257.0812	$C_{15}H_{14}O_4$	Dimer	-0.7	0.95	9.0	2.85	242		2		3
259.0604	$C_{14}H_{12}O_5$	Dimer	-0.8	0.88	9.0	3.62					3
271.0604	$C_{15}H_{12}O_5$	Dimer	-0.8	1.00	10.0	3.13					3
				1.04		4.53	256	MS^3			3
273.0759	$C_{15}H_{14}O_5$	Dimer	-0.9	1.00	9.0	3.28	258	MS^3	2		3
273.1124	$C_{16}H_{18}O_4$	Dimer	-0.8	°	8.0	3.99			2		3
				0.92		4.19	255, 243				3
287.0915	$C_{16}H_{16}O_5$	Dimer	-1.0	0.92	9.0	2.94		MS^3	2		2
				0.94		3.13	272, 257				3
299.0916	$C_{17}H_{16}O_5$	Dimer	-0.9	0.86	10.0	3.50			2		3
301.0707	$C_{16}H_{14}O_6$	Dimer	-1.0	0.96	10.0	1.26			2		3
				1.03		3.93		MS^3			2
				1.00		4.25		MS^3			3
301.1071	$C_{17}H_{18}O_5$	Dimer	-1.0	0.99	9.0	2.99	286, 255	MS^3			2
				0.92		5.48					3
301.1436	$C_{18}H_{22}O_4$	Dimer	-0.9	1.00	8.0	4.87					3
303.0865	$C_{16}H_{16}O_6$	Dimer	-0.9	0.90	9.0	3.69	273, 257		4		3
313.1434	$C_{19}H_{22}O_4$	Dimer	-1.1	0.98	9.0	5.18		MS^3			3
				1.00		5.75		MS^3			3
315.0862	$C_{17}H_{16}O_6$	Dimer	-1.2	1.07	10.0	1.26					3
				0.89		2.78					3
				1.08		4.00		MS^3			3
				0.85		5.12					3
315.1227	$C_{18}H_{20}O_5$	Dimer	-1.1	1.05	9.0	3.13		MS^3	2		2
317.1021	$C_{17}H_{18}O_6$	Dimer	-0.9	0.85	9.0	3.60	299, 287				3

323.0915	$C_{19}H_{16}O_5$	Dimer	-1.0	0.91	12.0	3.86					3
329.1018	$C_{18}H_{18}O_6$	Dimer	-1.2	1.00	10.0	1.26					3
				0.98		3.80		MS^2			2
				1.00		3.87	283	MS^2			3
				0.99		4.16		MS^2			3
				0.98		4.99	301				3
329.1383	$C_{19}H_{22}O_5$	Dimer	-1.1	0.99	9.0	2.99					3
				1.06		6.18		MS^3			3
331.1176	$C_{18}H_{20}O_6$	Dimer	-1.1	0.95	9.0	3.37					3
				1.00		4.69		MS^3			2
343.1176	$C_{19}H_{20}O_6$	Dimer	-1.1	1.05	10.0	3.72	313, 299		5		3
345.1332	$C_{19}H_{22}O_6$	Dimer	-1.0	0.89	9.0	3.52					3
				1.05		6.86		MS^3			3
347.1124	$C_{18}H_{20}O_7$	Dimer	-1.2	0.95	9.0	4.09	315, 301				3
				1.07		5.29		MS^3			3
351.1228	$C_{21}H_{20}O_5$	Dimer	-1.0	1.13	12.0	4.27			2		3
355.1175	$C_{20}H_{20}O_6$	Dimer	-1.2	0.91	11.0	4.12					3
				0.90		4.63					3
359.1488	$C_{20}H_{24}O_6$	Dimer	-1.2	1.00	9.0	4.47		MS^3			2
				0.96		4.66					3
				0.97		5.44					3
365.1018	$C_{21}H_{18}O_6$	Dimer	-1.2	0.92	13.0	4.84					3
				1.00		5.48					3
373.1281	$C_{20}H_{22}O_7$	Dimer	-1.2	1.09	10.0	3.66	343, 327		2		3
				0.94		4.46	345, 343				3
				0.93		5.29	5.29, 327				3
				0.88		5.45	343				3

				1.00		5.50					3
379.1386	$C_{19}H_{24}O_8$	Dimer	-1.2	0.94	8.0	5.22			7		3
399.1437	$C_{22}H_{24}O_7$	Dimer	-1.2	0.92	11.0	4.05					3
419.1700	$C_{22}H_{28}O_8$	Dimer	-1.1	0.88	9.0	4.77	389, 375				3
				0.89		4.83	389				3
				0.96		5.34					3

Table S5. MS^3 fragmentation of m/z 151.0403 detected in the Lignoboost Kraft lignin sample at retention time 1.28 min.

MS stage	m/z	Fragment-ion	Chemical formula	Mass difference in mDa	RDB
MS1	151.0403	$[M-H]^-$	$C_8H_7O_3$	0.8	5.5
MS2	136.0169	$[M-H-CH_3]^-$	$C_7H_4O_3$	0.8	6.0
MS3 (136.0169)	108.0219	$[M-H-CH_3-CO]^-$	$C_6H_4O_2$	0.7	5.0
	92.0270	$[M-H-CH_3-CO_2]^-$	C_6H_4O	0.8	5.0

Table S6. MS^3 fragmentation of m/z 177.0558 detected in the Lignoboost Kraft lignin sample at retention time 1.75 min.

MS stage	m/z	Fragment-ion	Chemical formula	Mass difference in mDa	RDB
MS1	177.0558	$[M-H]^-$	$C_{10}H_9O_3$	0.6	6.5
MS2	162.0322	$[M-H-CH_3]^-$	$C_9H_6O_3$	0.5	7.0
MS3 (162.0322)	134.0374	$[M-H-CH_3-CO]^-$	$C_8H_6O_2$	0.6	6.0

Table S7. MS^3 fragmentation of m/z 181.0506 detected in the Lignoboost Kraft lignin sample at retention time 1.98 min.

MS stage	m/z	Fragment-ion	Chemical formula	Mass difference in mDa	RDB
MS1	181.0506	$[M-H]^-$	$C_9H_9O_4$	0.5	5.5
MS2	166.0271	$[M-H-CH_3]^-$	$C_8H_6O_4$	0.5	6.0
	137.0609	$[M-H-CO_2]^-$	$C_8H_9O_2$	0.7	4.5
	122.0375	$[M-H-CH_3-CO_2]^-$	$C_7H_6O_2$	0.7	5.0
MS3 (166.0271)	149.0245	$[M-H-CH_3-OH]^-$	$C_8H_5O_3$	0.7	6.5
	136.0167	$[M-H-CH_3-CH_2O]^-$	$C_7H_4O_3$	0.7	6.0

Table S8. MS^3 fragmentation of m/z 231.0661 detected in the Lignoboost Kraft lignin sample at retention time 4.94 min.

MS stage	m/z	Fragment-ion	Chemical formula	Mass difference in mDa	RDB
MS1	231.0661	$[M-H]^-$	$C_{13}H_{11}O_4$	0.3	8.5
MS2	216.0425	$[M-H-CH_3]^-$	$C_{12}H_8O_4$	0.3	9.0
MS3 (216.0425)	188.0477	$[M-H-CH_3-CO]^-$	$C_{11}H_8O_3$	0.4	8.0
	187.0400	$[M-H-CH_3-CHO]^-$	$C_{11}H_7O_3$	0.5	8.5

Table S9. MS^2 fragmentation of m/z 245.0817 detected in the Lignoboost Kraft lignin sample at retention time 4.62 min.

MS stage	m/z	Fragment-ion	Chemical formula	Mass difference in mDa	RDB
MS1	245.0817	$[M-H]^-$	$C_{14}H_{13}O_4$	0.3	8.5
MS2	230.0580	$[M-H-CH_3]^-$	$C_{13}H_{10}O_4$	0.1	9.0

Table S10. MS^3 fragmentation of m/z 259.0972 detected in the Lignoboost Kraft lignin sample at retention time 4.45 min.

MS stage	m/z	Fragment-ion	Chemical formula	Mass difference in mDa	RDB
MS1	259.0972	$[M-H]^-$	$C_{15}H_{15}O_4$	0.2	8.5
MS2	244.0735	$[M-H-CH_3]^-$	$C_{14}H_{12}O_4$	0.0	9.0
MS3 (244.0735)	229.0502	$[M-H-CH_3-CH_3]^-$	$C_{13}H_9O_4$	0.2	9.5
	212.0477	$[M-H-CH_3-CH_3-HO]^-$	$C_{13}H_8O_3$	0.4	10.0
	211.0399	$[M-H-CH_3-CH_3-H_2O]^-$	$C_{13}H_7O_3$	0.4	10.5
	201.0556	$[M-H-CH_3-CH_3-H_2O]^-$	$C_{12}H_9O_3$	0.4	8.5
	173.0608	$[M-H-CH_3-CH_3-H_2O-CO]^-$	$C_{11}H_9O_2$	0.5	7.5
	159.0452	$[M-H-CH_3-CH_3-H_2O-CO-CH_2]^-$	$C_{10}H_7O_2$	0.6	7.5

Table S11. MS^3 fragmentation of m/z 269.0818 detected in the Lignoboost Kraft lignin sample at retention time 3.19 min.

MS stage	m/z	Fragment-ion	Chemical formula	Mass difference in mDa	RDB
MS1	269.0818	$[M-H]^-$	$C_{16}H_{13}O_4$	0.4	10.5
MS2	254.0578	$[M-H-CH_3]^-$	$C_{15}H_{10}O_4$	-0.1	11.0
	239.0345	$[M-H-CH_3-CH_3]^-$	$C_{14}H_7O_4$	0.1	11.5
MS3 (254.0578)	226.0632	$[M-H-CH_3-CO]^-$	$C_{14}H_{10}O_3$	0.2	10.0
MS3 (239.0345)	211.0398	$[M-H-CH_3-CH_3-CO]^-$	$C_{13}H_7O_3$	0.3	10.5

Table S12. MS^3 fragmentation of m/z 273.0764 detected in the Lignoboost Kraft lignin sample at retention time 3.26 min.

MS stage	m/z	Fragment-ion	Chemical formula	Mass difference in mDa	RDB
MS1	273.0764	$[M\text{-}H]^-$	$C_{15}H_{13}O_5$	0.1	9.5
MS2	258.0528	$[M\text{-}H\text{-}CH_3]^-$	$C_{14}H_{10}O_5$	0.0	10.0
MS3 (258.0528)	243.0295	$[M\text{-}H\text{-}CH_3\text{-}CH_3]^-$	$C_{13}H_7O_5$	0.2	10.5
	241.0504	$[M\text{-}H\text{-}CH_3\text{-}HO]^-$	$C_{14}H_9O_4$	0.3	10.5
	240.0504	$[M\text{-}H\text{-}CH_3\text{-}H_2O]^-$	$C_{14}H_8O_4$	0.3	11.0
	230.0582	$[M\text{-}H\text{-}CH_3\text{-}CO]^-$	$C_{13}H_{10}O_4$	0.3	9.0
	229.0502	$[M\text{-}H\text{-}CH_3\text{-}CHO]^-$	$C_{13}H_9O_4$	0.2	9.5
	212.0477	$M\text{-}H\text{-}CH_3\text{-}CH_2O_2]^-$	$C_{13}H_8O_3$	0.4	10.0
	210.0320	$[M\text{-}H\text{-}CH_3\text{-}CH_4O_2]^-$	$C_{13}H_6O_3$	0.3	11.0

Table S13. MS^3 fragmentation of m/z 273.0764 detected in the Lignoboost Kraft lignin sample at retention time 3.53 min.

MS stage	m/z	Fragment-ion	Chemical formula	Mass difference in mDa	RDB
MS1	273.0764	$[M\text{-}H]^-$	$C_{15}H_{13}O_5$	0.1	9.5
MS2	258.0527	$[M\text{-}H\text{-}CH_3]^-$	$C_{14}H_{10}O_5$	0.1	10.0
MS3 (258.0528)	243.0293	$[M\text{-}H\text{-}CH_3\text{-}CH_3]^-$	$C_{13}H_7O_5$	0.0	10.5
	240.0424	$[M\text{-}H\text{-}CH_3\text{-}H_2O]^-$	$C_{14}H_8O_4$	0.1	11.0

Table S14. MS^3 fragmentation of m/z 273.1127 detected in the Lignoboost Kraft lignin sample at retention time 4.36 min.

MS stage	m/z	Fragment-ion	Chemical formula	Mass difference in mDa	RDB
MS1	273.1127	$[M-H]^-$	$C_{16}H_{17}O_4$	0.1	8.5
MS2	258.0894	$[M-H-CH_3]^-$	$C_{15}H_{14}O_4$	0.2	9.0
	243.0661	$[M-H-CH_3-CH_3]^-$	$C_{14}H_{11}O_4$	0.4	9.5
	229.0503	$[M-H-CH_3-CH_3-CH_2]^-$	$C_{13}H_9O_4$	0.2	9.5
MS3 (243.0661)	228.0424	$[M-H-CH_3-CH_3-CH_3]^-$	$C_{13}H_8O_4$	0.2	10.0
	225.0554	$[M-H-CH_3-CH_3-H_2O]^-$	$C_{14}H_9O_3$	0.3	10.5
	215.0711	$[M-H-CH_3-CH_3-CO]^-$	$C_{13}H_{11}O_3$	0.3	8.5
	121.0297	$[M-H-CH_3-CH_3-C_7H_6O_2]^-$	$C_7H_5O_2$	0.8	5.5
MS3 (229.0503)	211.0398	$[M-H-CH_3-CH_3-CH_2-H_2O]^-$	$C_{13}H_7O_3$	0.3	10.5
	201.0555	$[M-H-CH_3-CH_3-CH_2-CO]^-$	$C_{12}H_9O_3$	0.3	8.5
	187.0400	$[M-H-CH_3-CH_3-CH_2-C_2H_2O]^-$	$C_{11}H_7O_3$	0.5	8.5
	173.0607	$[M-H-CH_3-CH_3-CH_2-C_2O_2]^-$	$C_{11}H_9O_2$	0.5	7.5

Table S15. MS^3 fragmentation of m/z 273.1127 detected in the Lignoboost Kraft lignin sample at retention time 4.19 min.

MS stage	m/z	Fragment-ion	Chemical formula	Mass difference in mDa	RDB
MS1	273.1127	$[M-H]^-$	$C_{16}H_{17}O_4$	0.1	8.5
MS2	258.0894	$[M-H-CH_3]^-$	$C_{15}H_{14}O_4$	0.2	9.0
	255.1025	$[M-H-CH_3-H_2O]^-$	$C_{16}H_{15}O_3$	0.4	9.5
	243.0661	$[M-H-CH_3-CH_3]^-$	$C_{14}H_{11}O_4$	0.4	9.5
MS3 (255.1025)	240.0789	$[M-H-CH_3-H_2O-CH_3]^-$	$C_{15}H_{12}O_3$	0.2	10.0

Table S16. MS^3 fragmentation of m/z 287.0920 detected in the Lignoboost Kraft lignin sample at retention time 3.44 min. Identified as a fragment of m/z 317.1026.

MS stage	m/z	Fragment-ion	Chemical formula	Mass difference in mDa	RDB
MS1	287.0920	$[M-H]^-$	$C_{16}H_{15}O_5$	0.1	9.5
MS2	272.0684	$[M-H-CH_3]^-$	$C_{15}H_{12}O_5$	-0.1	10.0
MS3 (272.0684)	243.0661	$[M-H-CH_3-CH_3]^-$	$C_{14}H_9O_5$	0.0	10.5
	254.0580	$[M-H-CH_3-H_2O]^-$	$C_{15}H_{10}O_4$	0.1	11.0
	243.0660	$[M-H-CH_3-CO]^-$	$C_{14}H_{11}O_4$	0.2	9.5

Table S17. MS^3 fragmentation of m/z 299.0921 detected in the Lignoboost Kraft lignin sample at retention time 3.75 min.

MS stage	m/z	Fragment-ion	Chemical formula	Mass difference in mDa	RDB
MS1	299.0921	$[M-H]^-$	$C_{17}H_{15}O_5$	0.2	10.5
MS2	284.0686	$[M-H-CH_3]^-$	$C_{16}H_{12}O_5$	0.1	11.0
	269.0453	$[M-H-CH_3-CH_3]^-$	$C_{15}H_9O_5$	0.3	11.5
MS3 (269.0453)	251.0345	$[M-H-CH_3-CH_3-H_2O]^-$	$C_{15}H_7O_4$	0.1	12.5
	241.0502	$[M-H-CH_3-CH_3-CO]^-$	$C_{14}H_9O_4$	0.1	10.5
	225.0554	$[M-H-CH_3-CH_3-CO_2]^-$	$C_{14}H_9O_3$	0.2	10.5
	197.0606	$[M-H-CH_3-CH_3-CO_2-CO]^-$	$C_{13}H_9O_2$	0.3	9.5

Table S18. MS^3 fragmentation of m/z 301.0716 detected in the Lignoboost Kraft lignin sample at retention time 1.29 min.

MS stage	m/z	Fragment-ion	Chemical formula	Mass difference in mDa	RDB
MS1	301.0716	$[M-H]^-$	$C_{16}H_{13}O_6$	0.4	10.5
MS2	286.0476	$[M-H-CH_3]^-$	$C_{15}H_{10}O_6$	0.1	11.0
	253.0502	$[M-H-CH_4O_2]^-$	$C_{15}H_9O_4$	0.2	11.5
MS3 (286.0476)	271.0241	$[M-H-CH_3-CH_3]^-$	$C_{14}H_7O_6$	-0.2	11.5
	269.0450	$[M-H-CH_3-OH]^-$	$C_{15}H_9O_5$	0.0	11.5
	268.0372	$[M-H-CH_3-H_2O]^-$	$C_{15}H_8O_5$	0.0	12.0
	258.0529	$[M-H-CH_3-CO]^-$	$C_{14}H_{10}O_5$	0.7	10.0
	257.0450	$[M-H-CH_3-CHO]^-$	$C_{14}H_9O_5$	0.0	10.5
	256.0373	$[M-H-CH_3-CH_2O]^-$	$C_{14}H_8O_5$	0.2	11.0
	240.0424	$[M-H-CH_3-CH_2O_2]^-$	$C_{14}H_8O_4$	0.1	11.0
	239.0373	$[M-H-CH_3-CH_3O_2]^-$	$C_{14}H_7O_4$	0.1	11.5
	229.0503	$[M-H-CH_3-C_2HO_2]^-$	$C_{13}H_9O_4$	0.2	9.5
	212.0476	$[M-H-CH_3-C_2H_2O_3]^-$	$C_{13}H_8O_3$	0.3	10.0
MS3 (253.0502)	238.0267	$[M-H-CH_4O_2-CH_3]^-$	$C_{14}H_6O_4$	0.1	12.0

Table S19. MS^3 fragmentation of m/z 301.0713 detected in the Lignoboost Kraft lignin sample at retention time 4.29 min.

MS stage	m/z	Fragment-ion	Chemical formula	Mass difference in mDa	RDB
MS1	301.0713	$[M-H]^-$	$C_{16}H_{13}O_6$	0.1	10.5
MS2	286.0477	$[M-H-CH_3]^-$	$C_{15}H_{10}O_6$	0.1	11.0
	253.0503	$[M-H-CH_4O_2]^-$	$C_{15}H_9O_4$	0.2	11.5
MS3 (253.0502)	238.0269	$[M-H-CH_4O_2-CH_3]^-$	$C_{14}H_6O_4$	0.3	12.0

Table S20. MS^3 fragmentation of m/z 301.1079 detected in the Lignoboost Kraft lignin sample at retention time 3.07 min. Identified as a fragment of m/z 331.1182.

MS stage	m/z	Fragment-ion	Chemical formula	Mass difference in mDa	RDB
MS1	301.1079	$[M-H]^-$	$C_{17}H_{17}O_5$	0.3	9.5
MS2	286.0840	$[M-H-CH_3]^-$	$C_{16}H_{14}O_5$	-0.2	10.0
	150.0322	$[M-H-C_9H_{11}O_2]^-$	$C_8H_6O_3$	0.6	6.0
	136.0167	$[M-H-C_{10}H_{13}O_2]^-$	$C_7H_4O_3$	0.6	6.0
	108.0219	$[M-H-C_{11}H_{13}O_3]^-$	$C_6H_4O_2$	0.8	5.0
MS3 (286.0840)	271.0605	$[M-H-CH_3-CH_3]^-$	$C_{15}H_{11}O_5$	-0.1	10.5
	230.0580	$[M-H-CH_3-C_3H_4O]^-$	$C_{13}H_{10}O_4$	0.0	9.0

Table S21. MS^3 fragmentation of m/z 303.0871 detected in the Lignoboost Kraft lignin sample at retention time 4.11 min.

MS stage	m/z	Fragment-ion	Chemical formula	Mass difference in mDa	RDB
MS1	303.0871	$[M-H]^-$	$C_{16}H_{15}O_6$	0.3	9.5
MS2	288.0635	$[M-H-CH_3]^-$	$C_{15}H_{12}O_6$	0.1	10.0
	285.0816	$[M-H-H_2O]^-$	$C_{16}H_{13}O_5$	0.2	10.5
	275.0923	$[M-H-CO]^-$	$C_{15}H_{15}O_5$	0.3	8.5
	255.0299	$[M-H-CH_3-CH_3-H_2O]^-$	$C_{14}H_7O_5$	0.5	11.5
	244.0739	$[M-H-C_2H_3O_2]^-$	$C_{14}H_{12}O_4$	0.4	9.0
	227.0711	$[M-H-C_2H_4O_3]^-$	$C_{14}H_{11}O_3$	0.3	9.5
	212.0477	$[M-H-C_3H_7O_3]^-$	$C_{13}H_8O_3$	0.4	10.0
MS3 (288.0635)	273.0401	$[M-H-CH_3-CH_3]^-$	$C_{14}H_9O_6$	0.2	10.5
	257.0452	$[M-H-CH_3-CH_3O]^-$	$C_{14}H_9O_5$	0.2	10.5
	230.0582	$[M-H-CH_3-C_2H_2O_2]^-$	$C_{13}H_{10}O_4$	0.2	9.0
	136.0167	$[M-H-CH_3-C_8H_8O_3]^-$	$C_7H_4O_3$	0.7	6.0
	108.0219	$[M-H-CH_3-C_9H_8O_4]^-$	$C_6H_4O_2$	0.8	5.0
MS3 (285.0816)	270.0531	$[M-H-H_2O-CH_3]^-$	$C_{15}H_{10}O_5$	0.3	11.0
	257.0816	$[M-H-H_2O-CO]^-$	$C_{15}H_{13}O_4$	0.2	9.5
MS3 (257.0816)	242.0578	$[M-H-CH_2O_2-CH_3]^-$	$C_{14}H_{10}O_4$	-0.1	10.0
	227.0345	$[M-H-CH_2O_2CH_3-CH_3]^-$	$C_{13}H_7O_4$	0.1	10.5

Table S22. MS^3 fragmentation of m/z 313.1075 detected in the Lignoboost Kraft lignin sample at retention time 3.65 min.

MS stage	m/z	Fragment-ion	Chemical formula	Mass difference in mDa	RDB
MS1	313.1075	$[M-H]^-$	$C_{18}H_{17}O_5$	-0.1	10.5
MS2	298.0839	$[M-H-CH_3]^-$	$C_{17}H_{14}O_5$	-0.2	11.0
	283.0606	$[M-H-CH_3-CH_3]^-$	$C_{16}H_{11}O_5$	0.0	11.5
MS3 (283.0606)	265.0501	$[M-H-CH_3-CH_3-H_2O]^-$	$C_{16}H_9O_4$	0.0	12.5
	255.0658	$[M-H-CH_3-CH_3-CO]^-$	$C_{15}H_{11}O_4$	0.1	10.5
	241.0502	$[M-H-CH_3-CH_3-C_2H_2O]^-$	$C_{14}H_9O_4$	0.1	10.5
	240.0424	$[M-H-CH_3-CH_3-C_2H_3O]^-$	$C_{14}H_8O_4$	0.1	11.0
	239.0710	$[M-H-CH_3-CH_3-CO_2]^-$	$C_{15}H_{11}O_3$	0.2	10.5
	237.0554	$[M-H-CH_3-CH_3-CH_2O_2]^-$	$C_{15}H_9O_3$	0.2	11.5
	224.0476	$[M-H-CH_3-CH_3-C_3H_6O_2]^-$	$C_{14}H_8O_3$	0.3	11.0
	211.0762	$[M-H-CH_3-CH_3-C_3H_3O_3]^-$	$C_{14}H_{11}O_2$	0.3	9.5
MS3 (298.0839)	283.0606	$[M-H-CH_3-CH_3]^-$	$C_{16}H_{11}O_5$	0.0	11.5

Table S23. MS^3 fragmentation of m/z 313.1075 detected in the Lignoboost Kraft lignin sample at retention time 5.46 min.

MS stage	m/z	Fragment-ion	Chemical formula	Mass difference in mDa	RDB
MS1	313.1075	$[M-H]^-$	$C_{18}H_{17}O_5$	-0.1	10.5
MS2	298.0839	$[M-H-CH_3]^-$	$C_{17}H_{14}O_5$	-0.2	11.0
MS3 (298.0839)	283.0606	$[M-H-CH_3-CH_3]^-$	$C_{16}H_{11}O_5$	0.0	11.5
	281.0813	$[M-H-CH_3-OH]^-$	$C_{17}H_{13}O_4$	-0.1	11.5
	270.0891	$[M-H-CH_3-CO]^-$	$C_{16}H_{14}O_4$	-0.1	10.0
	269.0814	$[M-H-CH_3-CHO]^-$	$C_{16}H_{13}O_4$	0.0	10.5
	268.0735	$[M-H-CH_3-CH_2O]^-$	$C_{16}H_{12}O_4$	0.0	11.0
	267.0658	$[M-H-CH_3-CH_3O]^-$	$C_{16}H_{11}O_4$	0.2	11.5
	254.0579	$[M-H-CH_3-C_2H_4O]^-$	$C_{15}H_{10}O_4$	0.0	11.0
	253.0501	$[M-H-CH_3-C_2H_5O]^-$	$C_{15}H_9O_4$	0.1	11.5
	251.0709	$[M-H-CH_3-CH_3O_2]^-$	$C_{16}H_{11}O_3$	0.0	11.5
	240.0424	$[M-H-CH_3-C_3H_6O]^-$	$C_{14}H_8O_4$	0.1	11.0
	225.0554	$[M-H-CH_3-C_3H_5O_2]^-$	$C_{14}H_9O_3$	0.2	10.5
	189.0556	$[M-H-CH_3-C_6H_5O_2]^-$	$C_{11}H_9O_3$	0.4	7.5
	176.0478	$[M-H-CH_3-C_7H_6O_2]^-$	$C_{10}H_8O_3$	0.5	7.0

Table S24. MS^3 fragmentation of m/z 315.0869 detected in the Lignoboost Kraft lignin sample at retention time 4.01 min.

MS stage	m/z	Fragment-ion	Chemical formula	Mass difference in mDa	RDB
MS1	315.0869	$[M-H]^-$	$C_{17}H_{15}O_6$	0.0	10.5
MS2	300.0633	$[M-H-CH_3]^-$	$C_{16}H_{12}O_6$	-0.1	11.0
	267.0660	$[M-H-CH_4O_2]^-$	$C_{16}H_{11}O_4$	0.3	11.5
	191.0349	$[M-H-C_7H_8O_2]^-$	$C_{10}H_7O_4$	0.5	7.5
MS3 (300.0633)	285.0399	$[M-H-CH_3-CH_3]^-$	$C_{15}H_9O_6$	-0.1	11.5
	283.0606	$[M-H-CH_3-HO]^-$	$C_{16}H_{11}O_5$	0.0	11.5
	282.0529	$[M-H-CH_3-H_2O]^-$	$C_{16}H_{10}O_5$	0.0	12.0
	281.0451	$[M-H-CH_3-H_3O]^-$	$C_{16}H_9O_5$	0.1	12.5
	271.0607	$[M-H-CH_3-CHO]^-$	$C_{15}H_{11}O_5$	0.1	10.5
	257.0451	$[M-H-CH_3-C_2H_3O]^-$	$C_{14}H_9O_5$	0.1	10.5
	254.0580	$[M-H-CH_3-CH_2O_2]^-$	$C_{15}H_{10}O_4$	0.1	11.0
	253.0503	$[M-H-CH_3-CH_3O_2]^-$	$C_{15}H_9O_4$	0.2	11.5
	243.0658	$[M-H-CH_3-C_2HO_2]^-$	$C_{14}H_{11}O_4$	0.1	9.5
	226.0632	$[M-H-CH_3-C_2H_2O_3]^-$	$C_{14}H_{10}O_3$	0.2	10.0
MS3 (267.0660)	252.0424	$[M-H-CH_4O_2-CH_3]^-$	$C_{15}H_8O_4$	0.2	12.0

Table S25. MS^3 fragmentation of m/z 315.1233 detected in the Lignoboost Kraft lignin sample at retention time 3.11 min.

MS stage	m/z	Fragment-ion	Chemical formula	Mass difference in mDa	RDB
MS1	315.1233	$[M-H]^-$	$C_{18}H_{19}O_5$	0.0	9.5
MS2	300.0997	$[M-H-CH_3]^-$	$C_{17}H_{16}O_5$	0.0	10.0
	285.0764	$[M-H-CH_3-CH_3]^-$	$C_{16}H_{13}O_5$	0.5	10.5
	178.0634	$[M-H-C_8H_9O_2]^-$	$C_{10}H_{10}O_3$	0.4	6.0
MS3 (300.0997)	285.0764	$[M-H-CH_3-CH_3]^-$	$C_{16}H_{13}O_5$	0.5	10.5
	164.0478	$[M-H-CH_3-C_8H_8O_2]^-$	$C_9H_8O_3$	0.5	6.0
MS3 (285.0764)	270.0527	$[M-H-CH_3-CH_3-CH_3]^-$	$C_{15}H_{10}O_5$	0.2	12.0
	242.0581	$[M-H-CH_3-CH_3-CH_3-CO]^-$	$C_{14}H_{10}O_4$	0.2	10.0
	163.0400	$[M-H-CH_3-CH_3-C_7H_6O_2]^-$	$C_9H_7O_3$	0.5	6.5

Table S26. MS^3 fragmentation of m/z 315.1233 detected in the Lignoboost Kraft lignin sample at retention time 5.99 min.

MS stage	m/z	Fragment-ion	Chemical formula	Mass difference in mDa	RDB
MS1	315.1233	$[M-H]^-$	$C_{18}H_{19}O_5$	0.0	9.5
MS2	300.0997	$[M-H-CH_3]^-$	$C_{17}H_{16}O_5$	0.0	10.0
MS3 (300.0997)	283.0790	$[M-H-CH_3-OH]^-$	$C_{17}H_{15}O_4$	0.0	10.5
	282.0893	$[M-H-CH_3-H_2O]^-$	$C_{17}H_{14}O_4$	0.1	11.0
	270.0892	$[M-H-CH_3-CH_2O]^-$	$C_{16}H_{14}O_4$	0.0	10.0
	269.0814	$[M-H-CH_3-CH_3O]^-$	$C_{16}H_{13}O_4$	0.0	10.5
	256.0737	$[M-H-CH_3-C_2H_4O]^-$	$C_{15}H_{12}O_4$	0.1	10.0
	255.0658	$[M-H-CH_3-C_2H_5O]^-$	$C_{15}H_{11}O_4$	0.1	10.5
	251.0709	$[M-H-CH_3-CH_5O_3]^-$	$C_{16}H_{11}O_3$	0.1	11.5
	243.0658	$[M-H-CH_3-C_3H_5O]^-$	$C_{14}H_{11}O_4$	0.1	9.5
	241.0502	$[M-H-CH_3-C_3H_7O]^-$	$C_{14}H_9O_4$	0.1	10.5
	240.0424	$[M-H-CH_3-C_3H_8O]^-$	$C_{14}H_8O_4$	0.1	11.0
	191.0712	$[M-H-CH_3-C_6H_5O_2]^-$	$C_{11}H_{11}O_3$	0.4	6.5
	178.0634	$[M-H-CH_3-C_7H_6O_2]^-$	$C_{10}H_{10}O_3$	0.4	6.0
	148.0530	$[M-H-CH_3-C_8H_8O_3]^-$	$C_9H_8O_2$	0.6	6.0

Table S27. MS^2 fragmentation of m/z 325.1075 detected in the Lignoboost Kraft lignin sample at retention time 4.33 min.

MS stage	m/z	Fragment-ion	Chemical formula	Mass difference in mDa	RDB
MS1	325.1075	$[M-H]^-$	$C_{19}H_{17}O_5$	-0.1	11.5
MS2	310.0841	$[M-H-CH_3]^-$	$C_{18}H_{14}O_5$	0.0	12.0

Table S28. MS^3 fragmentation of m/z 327.1232 detected in the Lignoboost Kraft lignin sample at retention time 3.53 min.

MS stage	m/z	Fragment-ion	Chemical formula	Mass difference in mDa	RDB
MS1	327.1232	$[M-H]^-$	$C_{19}H_{19}O_5$	-0.1	10.5
MS2	312.0996	$[M-H-CH_3]^-$	$C_{18}H_{16}O_5$	-0.2	11.0
MS3 (312.0996)	297.0760	$[M-H-CH_3-CH_3]^-$	$C_{17}H_{13}O_5$	-0.3	11.5

Table S29. MS^3 fragmentation of m/z 329.1025 detected in the Lignoboost Kraft lignin sample at retention time 4.39 min.

MS stage	m/z	Fragment-ion	Chemical formula	Mass difference in mDa	RDB
MS1	329.1025	$[M-H]^-$	$C_{18}H_{17}O_6$	0.0	10.5
MS2	314.0789	$[M-H-CH_3]^-$	$C_{17}H_{14}O_6$	-0.1	11.0
	299.0557	$[M-H-CH_3-CH_3]^-$	$C_{16}H_{11}O_6$	0.2	11.5
	285.1129	$[M-H-CH_3-CO_2]^-$	$C_{17}H_{17}O_4$	0.2	9.5
	270.0895	$[M-H-C_2H_3O_2]^-$	$C_{16}H_{14}O_4$	0.3	10.0
MS3 (314.0789)	299.0557	$[M-H-CH_3-CH_3]^-$	$C_{16}H_{11}O_6$	0.2	11.5
	284.0688	$[M-H-CH_3-CH_2O]^-$	$C_{16}H_{12}O_5$	0.4	11.0
	256.0739	$[M-H-CH_3-C_2H_2O_2]^-$	$C_{15}H_{12}O_4$	0.3	10.0
	192.0427	$[M-H-CH_3-C_7H_6O_2]^-$	$C_{10}H_8O_4$	0.5	7.0
MS3 (299.0557)	281.0448	$[M-H-CH_3-CH_3-H_2O]^-$	$C_{16}H_9O_5$	-0.2	12.5
	271.0606	$[M-H-CH_3-CH_3-CO]^-$	$C_{15}H_{11}O_5$	0.0	10.5
	251.0343	$[M-H-CH_3-CH_3CH_4O_2]^-$	$C_{15}H_7O_4$	-0.1	12.5
	241.0501	$[M-H-CH_3-CH_3-C_2H_2O_2]^-$	$C_{14}H_9O_4$	0.5	10.5

Table S30. MS^3 fragmentation of m/z 329.1389 detected in the Lignoboost Kraft lignin sample at retention time 4.76 min.

MS stage	m/z	Fragment-ion	Chemical formula	Mass difference in mDa	RDB
MS1	329.1389	$[M-H]^-$	$C_{19}H_{21}O_5$	-0.3	8.5
MS2	314.1153	$[M-H-CH_3]^-$	$C_{18}H_{18}O_5$	-0.4	9.0
	313.1086	$[M-H-CH_4]^-$	$C_{18}H_{17}O_5$	1.0	9.5
	299.0923	$[M-H-CH_3-CH_3]^-$	$C_{17}H_{15}O_5$	0.3	10.5
	285.1130	$[M-H-CH_3-CHO]^-$	$C_{17}H_{17}O_4$	0.8	9.5
MS3 (314.1153)	283.0972	$[M-H-CH_3-CH_3O]^-$	$C_{17}H_{15}O_4$	0.2	10.5
	281.0815	$[M-H-CH_3CH_5O]^-$	$C_{17}H_{13}O_4$	0.7	11.5
MS3 (299.0923)	269.0815	$[M-H-CH_3-CH_3-CH_2O]^-$	$C_{16}H_{13}O_4$	0.7	10.5
MS3 (285.1130)	270.0894	$[M-H-CH_3-CHO-CH_3]^-$	$C_{16}H_{14}O_4$	0.7	10.0

Table S31. MS^3 fragmentation of m/z 337.1076 detected in the Lignoboost Kraft lignin sample at retention time 5.00 min. Identified as a fragment of m/z 367.1182.

MS stage	m/z	Fragment-ion	Chemical formula	Mass difference in mDa	RDB
MS1	337.1076	$[M-H]^-$	$C_{20}H_{17}O_5$	0.5	12.5
MS2	322.0840	$[M-H-CH_3]^-$	$C_{19}H_{14}O_5$	0.4	13.0
	293.0816	$[M-H-CH_3-CHO]^-$	$C_{18}H_{13}O_4$	0.8	12.5
MS3 (322.0840)	307.0605	$[M-H-CH_3-CH_3]^-$	$C_{19}H_{11}O_5$	0.5	13.5
	305.0816	$[M-H-CH_3-OH]^-$	$C_{19}H_{13}O_4$	0.7	13.5
MS3 (293.0816)	278.0580	$[M-H-CH_3-CHO-CH_3]^-$	$C_{17}H_{10}O_4$	0.7	13.0
	265.0865	$[M-H-CH_3-CHO-CO]^-$	$C_{17}H_{13}O_3$	0.1	11.5

Table S32. MS^3 fragmentation of m/z 343.1181 detected in the Lignoboost Kraft lignin sample at retention time 4.21 min.

MS stage	m/z	Fragment-ion	Chemical formula	Mass difference in mDa	RDB
MS1	343.1181	$[M-H]^-$	$C_{19}H_{19}O_6$	-0.1	10.5
MS2	328.0946	$[M-H-CH_3]^-$	$C_{18}H_{16}O_6$	-0.1	11.0
	299.1284	$[M-H-CO_2]^-$	$C_{18}H_{19}O_4$	0.1	9.5
	148.0531	$[M-H-C_{10}H_{11}O_4]^-$	$C_9H_8O_2$	0.7	6.0
MS3 (328.0946)	313.0709	$[M-H-CH_3-CH_3]^-$	$C_{17}H_{13}O_6$	-0.4	11.5
	297.0762	$[M-H-CH_3-CH_3O]^-$	$C_{17}H_{13}O_5$	0.7	13.5
	284.1051	$[M-H-CH_3-CO_2]^-$	$C_{17}H_{16}O_4$	0.2	10.0
	269.0813	$[M-H-CH_3-C_2H_3O_2]^-$	$C_{16}H_{13}O_4$	-0.1	10.5
	204.0424	$[M-H-CH_3-C_7H_8O_2]^-$	$C_{11}H_8O_4$	0.2	8.0
	180.0426	$[M-H-CH_3-C_9H_8O_2]^-$	$C_9H_8O_4$	0.3	6.0
	166.0270	$[M-H-CH_3-C_{10}H_{10}O_2]^-$	$C_8H_6O_4$	0.4	6.0
	162.0685	$[M-H-CH_3-C_8H_6O_4]^-$	$C_{10}H_{10}O_2$	0.5	6.0
	147.0451	$[M-H-CH_3-C_9H_9O_4]^-$	$C_9H_7O_2$	0.5	6.5
	135.0452	$[M-H-CH_3-C_{10}H_9O_4]^-$	$C_8H_7O_2$	0.6	5.5
	122.0375	$[M-H-CH_3-C_{11}H_{10}O_4]^-$	$C_7H_6O_2$	0.7	5.0

Table S33. MS^3 fragmentation of m/z 343.1181 detected in the Lignoboost Kraft lignin sample at retention time 4.91 min.

MS stage	m/z	Fragment-ion	Chemical formula	Mass difference in mDa	RDB
MS1	343.1181	$[M-H]^-$	$C_{19}H_{19}O_6$	-0.1	10.5
MS2	328.0948	$[M-H-CH_3]^-$	$C_{18}H_{16}O_6$	0.1	11.0
	313.1078	$[M-H-CH_2O]^-$	$C_{18}H_{17}O_5$	0.2	10.5
	311.0922	$[M-H-CH_3-CHO]^-$	$C_{18}H_{15}O_5$	0.2	11.5
	299.1285	$[M-H-CO_2]^-$	$C_{18}H_{19}O_4$	0.2	9.5
	175.0765	$[M-H-C_8H_8O_4]^-$	$C_{11}H_{11}O_2$	0.6	6.5
	160.0530	$[M-H-C_9H_{11}O_4]^-$	$C_{10}H_8O_2$	0.6	7.0
	147.0453	$[M-H-C_{10}H_{12}O_4]^-$	$C_9H_7O_2$	0.7	6.5
MS3 (328.0948)	284.1050	$[M-H-CH_3-CO_2]^-$	$C_{17}H_{16}O_4$	0.2	10.0
MS3 (313.1078)	298.0840	$[M-H-CH_2O-CH_3]^-$	$C_{17}H_{14}O_5$	-0.1	11.0
	283.0606	$[M-H-CH_2O-CH_3-CH_3]^-$	$C_{16}H_{11}O_5$	0.0	11.5
	269.0812	$[M-H-CH_2O-CH_3-CHO]^-$	$C_{16}H_{13}O_4$	-0.2	10.5
MS3 (299.1285)	243.0658	$[M-H-CO_2-C_4H_8]^-$	$C_{14}H_{11}O_4$	0.1	9.5
	148.0531	$[M-H-CO_2-C_9H_{11}O_2]^-$	$C_9H_8O_2$	0.6	6.0

Table S34. MS^3 fragmentation of m/z 345.1338 detected in the Lignoboost Kraft lignin sample at retention time 4.60 min.

MS stage	m/z	Fragment-ion	Chemical formula	Mass difference in mDa	RDB
MS1	345.1338	$[M-H]^-$	$C_{19}H_{21}O_6$	0.0	9.5
MS2	330.1101	$[M-H-CH_3]^-$	$C_{18}H_{18}O_6$	-0.3	10.0
	327.1233	$[M-H-H_2O]^-$	$C_{19}H_{19}O_5$	0.1	10.5
MS3 (330.1101)	315.0870	$[M-H-CH_3-CH_3]^-$	$C_{17}H_{15}O_6$	0.2	10.5
	312.0999	$[M-H-CH_3-H_2O]^-$	$C_{18}H_{16}O_5$	0.1	11.0
	301.1078	$[M-H-CH_3-CHO]^-$	$C_{17}H_{17}O_5$	0.2	9.5
	300.0999	$[M-H-CH_3-CH_2O]^-$	$C_{17}H_{16}O_5$	0.2	10.0
	299.0922	$[M-H-CH_3-CH_3O]^-$	$C_{17}H_{15}O_5$	0.2	10.5
	297.0765	$[M-H-CH_3-CH_5O]^-$	$C_{17}H_{13}O_5$	0.2	11.5
	285.0765	$[M-H-CH_3-C_2H_5O]^-$	$C_{16}H_{13}O_5$	0.2	10.5
	272.0688	$[M-H-CH_3-C_3H_6O]^-$	$C_{15}H_{12}O_5$	0.4	10.0
	207.0661	$[M-H-CH_3-C_7H_7O_2]^-$	$C_{11}H_{11}O_4$	0.4	6.5
	193.0505	$[M-H-CH_3-C_8H_9O_2]^-$	$C_{10}H_9O_4$	0.5	6.5
	175.0401	$[M-H-CH_3-C_8H_{11}O_3]^-$	$C_{10}H_7O_3$	0.6	7.5
	165.0557	$[M-H-CH_3-C_9H_9O_3]^-$	$C_9H_9O_3$	0.6	5.5
	150.0323	$[M-H-CH_3-C_{10}H_{12}O_3]^-$	$C_8H_6O_3$	0.6	6.0
	136.0167	$[M-H-CH_3-C_{11}H_{14}O_3]^-$	$C_7H_4O_3$	0.7	6.0
	122.0375	$[M-H-CH_3-C_{11}H_{12}O_4]^-$	$C_7H_6O_2$	0.7	5.0
	108.0219	$[M-H-CH_3-C_{12}H_{14}O_4]^-$	$C_6H_4O_2$	0.8	5.0

Table S35. MS^2 fragmentation of m/z 349.1075 detected in the Lignoboost Kraft lignin sample at retention time 3.94 min.

MS stage	m/z	Fragment-ion	Chemical formula	Mass difference in mDa	RDB
MS1	349.1075	$[M-H]^-$	$C_{21}H_{17}O_5$	-0.1	13.5
MS2	334.0839	$[M-H-CH_3]^-$	$C_{20}H_{14}O_5$	-0.2	14.0

Table S36. MS^3 fragmentation of m/z 351.1232 detected in the Lignoboost Kraft lignin sample at retention time 4.26 min. Identified as a fragment of m/z 381.1339.

MS stage	m/z	Fragment-ion	Chemical formula	Mass difference in mDa	RDB
MS1	351.1232	$[M-H]^-$	$C_{21}H_{19}O_5$	-0.1	12.5
MS2	336.0994	$[M-H-CH_3]^-$	$C_{20}H_{16}O_5$	-0.3	13.0
	335.0913	$[M-H-CH_4]^-$	$C_{20}H_{15}O_5$	-0.6	13.5
MS3 (336.0994)	321.0759	$[M-H-CH_3-CH_3]^-$	$C_{19}H_{13}O_5$	-0.4	13.5

Table S37. MS^3 fragmentation of m/z 355.1180 detected in the Lignoboost Kraft lignin sample at retention time 4.03 min. Identified as a fragment of m/z 401.1234.

MS stage	m/z	Fragment-ion	Chemical formula	Mass difference in mDa	RDB
MS1	355.1180	$[M-H]^-$	$C_{20}H_{19}O_6$	0.4	11.5
MS2	340.0943	$[M-H-CH_3]^-$	$C_{19}H_{16}O_6$	0.1	12.0
MS3 (340.0943)	325.0709	$[M-H-CH_3-CH_3]^-$	$C_{18}H_{13}O_6$	0.2	12.5
	322.0839	$[M-H-CH_3-H_2O]^-$	$C_{19}H_{14}O_5$	0.3	13.0
	311.0917	$[M-H-CH_3-CHO]^-$	$C_{18}H_{15}O_5$	0.3	11.5
	296.0917	$[M-H-CH_3-CO_2]^-$	$C_{18}H_{16}O_4$	-0.2	11.0
	295.0969	$[M-H-CH_3-CHO_2]^-$	$C_{18}H_{15}O_4$	-0.1	11.5
	281.0813	$[M-H-CH_3-C_2H_3O_2]^-$	$C_{17}H_{13}O_4$	-0.1	11.5
	255.0657	$[M-H-CH_3-C_4H_5O_2]^-$	$C_{15}H_{11}O_4$	0.0	10.5
	173.0607	$[M-H-CH_3-C_8H_7O_4]^-$	$C_{11}H_9O_2$	0.4	7.5

Table S38. MS^3 fragmentation of m/z 357.1337 detected in the Lignoboost Kraft lignin sample at retention time 3.46 min.

MS stage	m/z	Fragment-ion	Chemical formula	Mass difference in mDa	RDB
MS1	357.1337	$[M-H]^-$	$C_{20}H_{21}O_6$	-0.1	10.5
MS2	342.1102	$[M-H-CH_3]^-$	$C_{19}H_{18}O_6$	-0.2	11.0
	313.1439	$[M-H-CO_2]^-$	$C_{19}H_{21}O_4$	-0.1	9.5
	298.1206	$[M-H-CH_3-CO_2]^-$	$C_{18}H_{18}O_4$	0.1	10.0
	209.0817	$[M-H-C_9H_8O_2]^-$	$C_{11}H_{13}O_4$	0.4	5.5
	191.0713	$[M-H-C_9H_{10}O_3]^-$	$C_{11}H_{11}O_3$	0.5	6.5
	147.0452	$[M-H-C_{11}H_{14}O_4]^-$	$C_9H_7O_2$	0.6	6.5
MS3 (342.1102)	327.0869	$[M-H-CH_3-CH_3]^-$	$C_{18}H_{15}O_6$	0.0	11.5
	324.0999	$[M-H-CH_3-H_2O]^-$	$C_{19}H_{16}O_5$	0.1	12.0
	313.1076	$[M-H-CH_3-CHO]^-$	$C_{18}H_{17}O_5$	0.0	10.5
	286.0844	$[M-H-CH_3-C_3H_4O]^-$	$C_{16}H_{14}O_5$	0.3	10.0
	219.0660	$[M-H-CH_3-C_7H_7O_2]^-$	$C_{12}H_{11}O_4$	0.3	7.5
	205.0503	$[M-H-CH_3-C_8H_9O_2]^-$	$C_{11}H_9O_4$	0.2	7.5
	163.0400	$[M-H-CH_3-C_{10}H_{11}O_3]^-$	$C_9H_7O_3$	0.5	6.5
	122.0375	$[M-H-CH_3-C_{12}H_{12}O_4]^-$	$C_7H_6O_2$	0.7	5.0

Table S39. MS^3 fragmentation of m/z 357.1338 detected in the Lignoboost Kraft lignin sample at retention time 3.69 min.

MS stage	m/z	Fragment-ion	Chemical formula	Mass difference in mDa	RDB
MS1	357.1338	$[M-H]^-$	$C_{20}H_{21}O_6$	0.0	10.5
MS2	342.1102	$[M-H-CH_3]^-$	$C_{19}H_{18}O_6$	-0.2	11.0
	313.1439	$[M-H-CO_2]^-$	$C_{19}H_{21}O_4$	-0.1	9.5
	298.1206	$[M-H-CH_3-CO_2]^-$	$C_{18}H_{18}O_4$	0.1	10.0
	281.1206	$[M-H-C_2H_4O_3]^-$	$C_{18}H_{17}O_3$	0.2	10.5
	221.0817	$[M-H-C_8H_8O_2]^-$	$C_{12}H_{13}O_4$	0.3	6.5
	209.0817	$[M-H-C_9H_8O_2]^-$	$C_{11}H_{13}O_4$	0.4	5.5
	191.0713	$[M-H-C_9H_{10}O_3]^-$	$C_{11}H_{11}O_3$	0.5	6.5
	147.0452	$[M-H-C_{11}H_{14}O_4]^-$	$C_9H_7O_2$	0.6	6.5
MS3 (342.1102)	205.0503	$[M-H-CH_3-C_8H_9O_2]^-$	$C_{11}H_9O_4$	0.2	7.5
	122.0375	$[M-H-CH_3-C_{12}H_{12}O_4]^-$	$C_7H_6O_2$	0.7	5.0

Table S40. MS^3 fragmentation of m/z 361.1287 detected in the Lignoboost Kraft lignin sample at retention time 5.57 min.

MS stage	m/z	Fragment-ion	Chemical formula	Mass difference in mDa	RDB
MS1	361.1287	$[M-H]^-$	$C_{19}H_{21}O_7$	-0.1	9.5
MS2	317.1389	$[M-H-CO_2]^-$	$C_{18}H_{21}O_5$	0.1	8.5
	287.1286	$[M-H-C_2H_2O_3]^-$	$C_{17}H_{19}O_4$	0.3	8.5
	149.0609	$[M-H-C_{10}H_{12}O_5]^-$	$C_9H_9O_2$	0.7	5.5
MS3 (317.1389)	302.1153	$[M-H-CO_2-CH_3]^-$	$C_{17}H_{18}O_5$	-0.1	9.0
	287.1286	$[M-H-CO_2-CH_2O]^-$	$C_{17}H_{19}O_4$	0.3	8.5
	272.1049	$[M-H-CO_2-C_2H_5O]^-$	$C_{16}H_{16}O_4$	0.0	9.0
	255.1022	$[M-H-CO_2-C_2H_6O_2]^-$	$C_{16}H_{15}O_3$	0.1	9.5
	135.0453	$[M-H-CO_2-C_{10}H_{14}O_3]^-$	$C_8H_7O_2$	0.7	5.5

Table S41. MS^3 fragmentation of m/z 361.1650 detected in the Lignoboost Kraft lignin sample at retention time 5.05 min.

MS stage	m/z	Fragment-ion	Chemical formula	Mass difference in mDa	RDB
MS1	361.1650	$[M-H]^-$	$C_{20}H_{25}O_6$	-0.2	8.5
MS2	346.1417	$[M-H-CH_3]^-$	$C_{19}H_{22}O_6$	0.0	9.0
	343.1548	$[M-H-H_2O]^-$	$C_{20}H_{23}O_5$	0.2	9.5
	331.1548	$[M-H-CH_2O]^-$	$C_{19}H_{23}O_5$	0.2	8.5
	313.1442	$[M-H-CH_4O_2]^-$	$C_{19}H_{21}O_4$	0.2	9.5
	298.1208	$[M-H-C_2H_7O_2]^-$	$C_{18}H_{18}O_4$	0.3	10.0
	179.0714	$[M-H-C_{10}H_{14}O_3]^-$	$C_{10}H_{11}O_3$	0.6	5.5
	165.0558	$[M-H-C_9H_9O_3]^-$	$C_{11}H_{16}O_3$	0.7	5.5
	147.0454	$[M-H-C_{11}H_{18}O_4]^-$	$C_9H_7O_2$	0.8	6.5
	122.0376	$[M-H-C_{13}H_{19}O_4]^-$	$C_7H_6O_2$	0.9	5.0
MS3 (331.1548)	316.1311	$[M-H-CH_2O-CH_3]^-$	$C_{18}H_{20}O_5$	0.0	9.0
	180.0790	$[M-H-C_9H_{11}O_2]^-$	$C_{10}H_{12}O_3$	0.3	5.0
MS3 (313.1442)	298.1208	$[M-H-CH_4O_2-CH_3]^-$	$C_{18}H_{18}O_4$	0.3	10.0

Table S42. MS^3 fragmentation of m/z 419.1494 detected in the Lignoboost Kraft lignin sample at retention time 5.34 min.

MS stage	m/z	Fragment-ion	Chemical formula	Mass difference in mDa	RDB
MS1	419.1494	$[M-H]^-$	$C_{25}H_{23}O_6$	-0.1	14.5
MS2	404.1255	$[M-H-CH_3]^-$	$C_{24}H_{20}O_6$	-0.5	15.0
	389.1024	$[M-H-CH_3-CH_3]^-$	$C_{23}H_{17}O_6$	-0.1	15.5
MS3 (389.1024)	374.0786	$[M-H-CH_3-CH_3-CH_3]^-$	$C_{22}H_{14}O_6$	-0.4	16.0
	371.0916	$[M-H-CH_3-CH_3-H_2O]^-$	$C_{23}H_{15}O_5$	-0.4	16.5
	240.0422	$[M-H-CH_3-CH_3-C_9H_9O_2]^-$	$C_{14}H_8O_4$	0.0	11.0

Table S43. MS^3 fragmentation of m/z 491.1704 detected in the Lignoboost Kraft lignin sample at retention time 5.71 min.

MS stage	m/z	Fragment-ion	Chemical formula	Mass difference in mDa	RDB
MS1	491.1704	$[M-H]^-$	$C_{28}H_{27}O_8$	-0.2	15.5
MS2	476.1469	$[M-H-CH_3]^-$	$C_{27}H_{24}O_8$	-0.2	16.0
	447.1804	$[M-H-CO_2]^-$	$C_{27}H_{17}O_6$	-0.2	14.5
MS3 (476.1469)	461.1236	$[M-H-CH_3-CH_3]^-$	$C_{26}H_{21}O_8$	0.0	16.5
	432.1573	$[M-H-CH_3-CO_2]^-$	$C_{26}H_{24}O_6$	0.1	15.0
	417.1339	$[M-H-CH_3-C_2H_3O_2]^-$	$C_{25}H_{21}O_6$	0.1	15.5
MS3 (447.1804)	310.1203	$[M-H-CO_2-C_8H_9O_2]^-$	$C_{19}H_{18}O_4$	-0.2	11.0
	296.1047	$[M-H-CO_2-C_9H_{11}O_2]^-$	$C_{18}H_{16}O_4$	-0.2	11.0
	270.0891	$[M-H-CO_2-C_{11}H_{13}O_2]^-$	$C_{16}H_{14}O_4$	-0.1	10.0

Table S44. MS^3 fragmentation of m/z 509.2176 detected in the Lignoboost Kraft lignin sample at retention time 6.36 min.

MS stage	m/z	Fragment-ion	Chemical formula	Mass difference in mDa	RDB
MS1	509.2176	$[M-H]^-$	$C_{29}H_{33}O_8$	0.1	13.5
MS2	494.1940	$[M-H-CH_3]^-$	$C_{28}H_{30}O_8$	-0.1	14.0
MS3 (494.1940)	479.1708	$[M-H-CH_3-CH_3]^-$	$C_{27}H_{27}O_8$	0.3	14.5
	463.1758	$[M-H-CH_3-CH_3O]^-$	$C_{27}H_{27}O_7$	0.2	14.5
	449.1603	$[M-H-CH_3-C_2H_5O]^-$	$C_{26}H_{25}O_7$	0.3	14.5
	327.1235	$[M-H-CH_3-C_9H_{11}O_3]^-$	$C_{19}H_{19}O_5$	0.2	10.5
	313.1078	$[M-H-CH_3-C_{10}H_{13}O_3]^-$	$C_{18}H_{17}O_5$	0.2	10.5
	295.0974	$[M-H-CH_3-C_{10}H_{15}O_4]^-$	$C_{18}H_{15}O_4$	0.3	11.5
	283.0973	$[M-H-CH_3-C_{11}H_{15}O_4]^-$	$C_{17}H_{15}O_4$	0.3	10.5
	281.0817	$[M-H-CH_3-C_{11}H_{17}O_4]^-$	$C_{17}H_{13}O_4$	0.3	11.5
	269.0817	$[M-H-CH_3-C_{12}H_{17}O_4]^-$	$C_{16}H_{13}O_4$	0.4	10.5
	255.0661	$[M-H-CH_3-C_{13}H_{19}O_4]^-$	$C_{15}H_{11}O_4$	0.4	10.5

Table S45. MS^2 fragmentation of m/z 163.0399 detected in the Lignosulphonate lignin sample at retention time 2.35 min.

MS stage	m/z	Fragment-ion	Chemical formula	Mass difference in mDa	RDB
MS1	163.0399	$[M-H]^-$	$C_9H_7O_3$	0.4	6.5
MS2	93.0348	$[M-H-C_3H_2O_2]^-$	C_6H_5O	0.8	4.5

Table S46. MS^3 fragmentation of m/z 195.0659 detected in the Lignosulphonate lignin sample at retention time 1.82 min.

MS stage	m/z	Fragment-ion	Chemical formula	Mass difference in mDa	RDB
MS1	195.0659	$[M-H]^-$	$C_{10}H_{11}O_4$	0.1	5.5
MS2	180.0428	$[M-H-CH_3]^-$	$C_8H_8O_4$	0.5	6.0
	177.0558	$[M-H-H_2O]^-$	$C_{10}H_9O_3$	0.7	6.5
MS3 (180.0428)	137.0245	$[M-H-CH_3-CH_3-CO]^-$	$C_7H_5O_3$	0.6	5.5
	136.0245	$[M-H-CH_3-CH_3-CHO]^-$	$C_7H_4O_3$	0.6	6.0
MS3 (177.0558)	162.0323	$[M-H-H_2O-CH_3]^-$	$C_9H_6O_3$	0.6	7.0
	149.0608	$[M-H-H_2O-CO]^-$	$C_9H_9O_2$	0.6	5.5
	134.0374	$[M-H-H_2O-CH_3-CO]^-$	$C_8H_6O_2$	0.7	6.0

Table S47. MS^3 fragmentation of m/z 287.0917 detected in the Lignosulphonate lignin sample at retention time 3.44 min.

MS stage	m/z	Fragment-ion	Chemical formula	Mass difference in mDa	RDB
MS1	287.0917	$[M-H]^-$	$C_{16}H_{15}O_5$	-0.3	9.5
MS2	272.0685	$[M-H-CH_3]^-$	$C_{15}H_{12}O_5$	0.0	10.0
	136.0167	$[M-H-C_9H_{11}O_2]^-$	$C_7H_4O_3$	0.6	6.0
	108.0219	$[M-H-C_{10}H_{11}O_3]^-$	$C_6H_4O_2$	0.8	5.0
MS3 (272.0685)	150.0323	$[M-H-CH_3-C_7H_6O_2]^-$	$C_8H_6O_3$	0.6	6.0
	136.0167	$[M-H-CH_3-C_8H_8O_2]^-$	$C_7H_4O_3$	0.6	6.0
	108.0219	$[M-H-CH_3-C_9H_8O_3]^-$	$C_6H_4O_2$	0.8	5.0

Table S48. MS^3 fragmentation of m/z 343.1538 detected in the Lignosulphonate lignin sample at retention time 3.44 min. Identified as fragment of m/z 389.1593.

MS stage	m/z	Fragment-ion	Chemical formula	Mass difference in mDa	RDB
MS1	343.1538	$[M-H]^-$	$C_{20}H_{23}O_5$	-0.7	9.5
MS2	328.1309	$[M-H-CH_3]^-$	$C_{19}H_{20}O_5$	-0.2	10.0
MS3 (328.1309)	313.1075	$[M-H-CH_3-CH_3]^-$	$C_{18}H_{17}O_5$	-0.2	10.5
	299.1282	$[M-H-CH_3-CHO]^-$	$C_{18}H_{19}O_4$	-0.1	9.5
	240.0788	$[M-H-CH_3-C_4H_8O_2]^-$	$C_{15}H_{12}O_3$	0.1	10.0
	203.0711	$[M-H-CH_3-C_7H_9O_2]^-$	$C_{12}H_{11}O_3$	0.3	7.5
	191.0712	$[M-H-CH_3-C_8H_9O_2]^-$	$C_{11}H_{11}O_3$	0.4	6.5
	177.0556	$[M-H-CH_3-C_9H_{11}O_2]^-$	$C_{10}H_9O_3$	0.5	6.5
	163.0400	$[M-H-CH_3-C_{10}H_{13}O_2]^-$	$C_9H_7O_3$	0.5	6.5
	135.0452	$[M-H-CH_3-C_{11}H_{13}O_3]^-$	$C_8H_7O_2$	0.6	5.5
	122.0375	$[M-H-CH_3-C_{12}H_{14}O_3]^-$	$C_7H_6O_2$	0.7	5.0

Table S49. MS^3 fragmentation of m/z 371.1122 detected in the Lignosulphonate lignin sample at retention time 3.96 min.

MS stage	m/z	Fragment-ion	Chemical formula	Mass difference in mDa	RDB
MS1	371.1122	$[M-H]^-$	$C_{20}H_{19}O_7$	-0.9	11.5
MS2	356.0893	$[M-H-CH_3]^-$	$C_{19}H_{16}O_7$	0.0	12.0
	341.1025	$[M-H-CH_2O]^-$	$C_{19}H_{17}O_6$	0.0	11.5
	327.1233	$[M-H-CO_2]^-$	$C_{19}H_{19}O_5$	0.0	10.5
	312.0998	$[M-H-CH_3-CO_2]^-$	$C_{18}H_{16}O_5$	0.0	11.0
	205.0504	$[M-H-C_9H_{10}O_3]^-$	$C_{11}H_9O_4$	0.4	7.5
	190.0271	$[M-H-C_{10}H_{13}O_3]^-$	$C_{10}H_6O_4$	0.5	8.0
MS3 (356.0893)	341.0661	$[M-H-CH_3-CH_3]^-$	$C_{18}H_{13}O_7$	-0.1	12.5
	339.0868	$[M-H-CH_3-OH]^-$	$C_{19}H_{15}O_6$	-0.1	12.5
	338.0789	$[M-H-CH_3-H_2O]^-$	$C_{19}H_{14}O_6$	-0.1	13.0
	311.0919	$[M-H-CH_3-CHO_2]^-$	$C_{18}H_{15}O_5$	0.0	11.5
	136.0166	$[M-H-CH_3-C_{12}H_{12}O_4]^-$	$C_7H_4O_3$	0.6	6.0
MS3 (327.1233)	175.0401	$[M-H-CO_2-C_9H_{12}O_2]^-$	$C_{10}H_7O_3$	0.5	7.5
MS3 (341.1025)	326.0789	$[M-H-CH_2O-CH_3]^-$	$C_{18}H_{14}O_6$	-0.2	12.0
	299.0919	$[M-H-CH_2O-C_2H_2O]^-$	$C_{17}H_{15}O_5$	0.0	10.5
	298.0842	$[M-H-CH_2O-C_2H_3O]^-$	$C_{17}H_{14}O_5$	0.1	11.0

Table S50. MS^3 fragmentation of m/z 373.1277 detected in the Lignosulphonate lignin sample at retention time 4.46 min. Identified as fragment of m/z 403.1388.

MS stage	m/z	Fragment-ion	Chemical formula	Mass difference in mDa	RDB
MS1	373.1277	$[M-H]^-$	$C_{20}H_{21}O_7$	-1.1	10.5
MS2	358.1051	$[M-H-CH_3]^-$	$C_{19}H_{18}O_7$	-0.2	11.0
	355.1180	$[M-H-H_2O]^-$	$C_{19}H_{19}O_6$	-0.2	11.5
	340.0946	$[M-H-CH_3+H_2O]^-$	$C_{19}H_{16}O_6$	-0.2	12.0
	327.1230	$[M-H-CH_2O_2]^-$	$C_{19}H_{19}O_5$	-0.2	10.5
	311.1283	$[M-H-CH_2O_3]^-$	$C_{19}H_{19}O_4$	0.0	10.5
	249.0763	$[M-H-C_7H_8O_2]^-$	$C_{13}H_{13}O_5$	0.0	7.5
	235.0607	$[M-H-C_8H_{10}H_2]^-$	$C_{12}H_{11}O_5$	0.1	7.5
	223.0608	$[M-H-C_9H_{10}O_2]^-$	$C_{11}H_{11}O_5$	0.3	6.5
	221.0452	$[M-H-C_9H_{12}O_2]^-$	$C_{11}H_9O_5$	0.2	7.5
	136.00530	$[M-H-CH_3-C_{12}H_{12}O_4]^-$	$C_8H_8O_2$	0.6	5.0
MS3 (355.1180)	337.1077	$[M-H-H_2O-H_2O]^-$	$C_{20}H_{17}O_5$	0.1	12.5
	204.0426	$[M-H-H_2O-C_9H_{11}O_2]^-$	$C_{11}H_8O_4$	0.4	8.0
MS3 (327.1230)	312.0992	$[M-H-CH_2O_2-CH_3]^-$	$C_{18}H_{16}O_5$	-0.2	11.0
	147.0452	$[M-H-CH_2O_2-C_{10}H_{12}O_3]^-$	$C_9H_7O_2$	0.5	6.5

Table S51. MS^2 fragmentation of m/z 403.1384 detected in the Lignosulphonate lignin sample at retention time 4.46 min.

MS stage	m/z	Fragment-ion	Chemical formula	Mass difference in mDa	RDB
MS1	403.1384	$[M-H]^-$	$C_{21}H_{23}O_8$	-0.9	10.5
MS2	373.1277	$[M-H-CH_2O]^-$	$C_{20}H_{21}O_7$	-1.1	10.5

Table S52. MS^3 fragmentation of m/z 357.1330 detected in the Lignosulphonate lignin sample at retention time 4.46 min. Identified as fragment of m/z 403.1388.

MS stage	m/z	Fragment-ion	Chemical formula	Mass difference in mDa	RDB
MS1	357.1330	$[M-H]^-$	$C_{20}H_{21}O_6$	-0.8	10.5
MS2	342.1101	$[M-H-CH_3]^-$	$C_{19}H_{18}O_6$	-0.2	11.0
	339.1232	$[M-H-H_2O]^-$	$C_{20}H_{19}O_5$	0.0	11.5
	313.1438	$[M-H-CO_2]^-$	$C_{19}H_{21}O_4$	-0.2	9.5
	298.1205	$[M-H-CH_3-CO_2]^-$	$C_{18}H_{18}O_4$	0.0	10.0
	281.1178	$[M-H-C_2H_4O_3]^-$	$C_{18}H_{17}O_3$	0.1	10.5
	211.0816	$[M-H-C_8H_8O_2]^-$	$C_{12}H_{13}O_4$	0.3	6.5
	209.0817	$[M-H-C_9H_8O_2]^-$	$C_{11}H_{13}O_4$	0.3	5.5
	191.0712	$[M-H-C_9H_{10}O_3]^-$	$C_{11}H_{11}O_3$	0.4	6.5
	161.0608	$[M-H- C_{10}H_{12}O_4]^-$	$C_{10}H_9O_2$	0.6	6.5
	147.0452	$[M-H-C_{11}H_{14}O_4]^-$	$C_9H_7O_2$	0.6	6.5
	121.0297	$[M-H-CH_3-C_{11}H_{13}O_3]^-$	$C_7H_5O_2$	0.8	5.5
MS3 (342.1101)	324.0994	$[M-H-CH_3-H_2O]^-$	$C_{19}H_{16}O_5$	-0.4	12.0
	313.1077	$[M-H-CH_3-CHO]^-$	$C_{18}H_{17}O_5$	-0.2	11.0
	298.1208	$[M-H-CH_3-CO_2]^-$	$C_{18}H_{18}O_4$	0.3	10.0
	205.0504	$[M-H-CH_3-C_9H_9O_2]^-$	$C_{11}H_9O_4$	0.3	7.5
	122.0375	$[M-H-CH_3-C_{12}H_{12}O_4]^-$	$C_7H_6O_2$	0.7	5.0
MS3 (313.1438)	283.0971	$[M-H-CO_2-C_2H_6]^-$	$C_{17}H_{15}O_4$	0.1	10.5

Table S53. MS^2 fragmentation of m/z 135.0450 detected in the depolymerised Kraft lignin sample at retention time 2.51 min.

MS stage	m/z	Fragment-ion	Chemical formula	Mass difference in mDa	RDB
MS1	135.0450	$[M-H]^-$	$C_8H_7O_2$	0.5	5.5
MS2	93.0349	$[M-H-C_2H_2O]^-$	C_6H_5O	0.8	4.5

Table S54. MS^2 fragmentation of m/z 137.0243 detected in the depolymerised Kraft lignin sample at retention time 4.05 min.

MS stage	m/z	Fragment-ion	Chemical formula	Mass difference in mDa	RDB
MS1	137.0243	$[M-H]^-$	$C_7H_5O_3$	0.4	5.5
MS2	109.0297	$[M-H-CO]^-$	$C_6H_5O_2$	0.7	4.5
	93.0349	$[M-H-CO_2]^-$	C_6H_5O	0.9	4.5

Table S55. MS^3 fragmentation of m/z 151.0399 detected in the depolymerised Kraft lignin sample at retention time 1.25 min.

MS stage	m/z	Fragment-ion	Chemical formula	Mass difference in mDa	RDB
MS1	151.0399	$[M-H]^-$	$C_8H_7O_3$	0.4	5.5
MS2	136.0166	$[M-H-CH_3]^-$	$C_7H_4O_3$	0.6	6.0
MS3 (136.0166)	108.0219	$[M-H-CH_3-CO]^-$	$C_6H_4O_2$	0.8	5.0
	92.0271	$[M-H-CH_3-CO_2]^-$	C_6H_4O	0.9	5.0

Table S56. MS^2 fragmentation of m/z 165.0554 detected in the depolymerised Kraft lignin sample at retention time 2.23 min.

MS stage	m/z	Fragment-ion	Chemical formula	Mass difference in mDa	RDB
MS1	165.0554	$[M-H]^-$	$C_9H_9O_3$	0.3	5.5
MS2	150.0323	$[M-H-CH_3]^-$	$C_8H_6O_3$	0.7	6.0

Table S57. MS^3 fragmentation of m/z 181.0503 detected in the depolymerised Kraft lignin sample at retention time 1.63 min.

MS stage	m/z	Fragment-ion	Chemical formula	Mass difference in mDa	RDB
MS1	181.0503	$[M-H]^-$	$C_9H_9O_4$	0.2	5.5
MS2	166.0270	$[M-H-CH_3]^-$	$C_8H_6O_4$	0.4	6.0
MS3 (166.0270)	151.0037	$[M-H-CH_3-CH_3]^-$	$C_7H_3O_4$	0.6	6.5

Table S58. MS^3 fragmentation of m/z 181.0503 detected in the depolymerised Kraft lignin sample at retention time 3.68 min.

MS stage	m/z	Fragment-ion	Chemical formula	Mass difference in mDa	RDB
MS1	181.0503	$[M-H]^-$	$C_9H_9O_4$	0.2	5.5
MS2	166.0271	$[M-H-CH_3]^-$	$C_8H_6O_4$	0.5	6.0
MS3 (166.0270)	151.0037	$[M-H-CH_3-CH_3]^-$	$C_7H_3O_4$	0.6	6.5
	138.0323	$[M-H-CH_3-CO]^-$	$C_7H_6O_3$	0.6	5.0

Table S59. MS^3 fragmentation of m/z 271.0604 detected in the depolymerised Kraft lignin sample at retention time 4.53 min.

MS stage	m/z	Fragment-ion	Chemical formula	Mass difference in mDa	RDB
MS1	271.0604	$[M-H]^-$	$C_{15}H_{11}O_5$	-0.3	10.5
MS2	256.0372	$[M-H-CH_3]^-$	$C_{14}H_8O_5$	0.0	11.0
MS3 (256.0372)	239.0347	$[M-H-CH_3-OH]^-$	$C_{14}H_7O_4$	0.3	11.5
	238.0270	$[M-H-CH_3-H_2O]^-$	$C_{14}H_6O_4$	0.4	12.0
	228.0425	$[M-H-CH_3-CO]^-$	$C_{13}H_8O_4$	0.3	10.0
	210.0321	$[M-H-CH_3-CH_2O_2]^-$	$C_{13}H_6O_3$	0.5	11.0

Table S60. MS^3 fragmentation of m/z 273.0759 detected in the depolymerised Kraft lignin sample at retention time 3.28 min.

MS stage	m/z	Fragment-ion	Chemical formula	Mass difference in mDa	RDB
MS1	273.0759	$[M-H]^-$	$C_{15}H_{13}O_5$	0.2	9.5
MS2	258.0532	$[M-H-CH_3]^-$	$C_{14}H_{10}O_5$	0.1	10.0
MS3 (258.0532)	243.0294	$[M-H-CH_3-CH_3]^-$	$C_{13}H_7O_5$	0.1	10.5
	241.0503	$[M-H-CH_3-OH]^-$	$C_{14}H_9O_4$	0.2	10.5
	240.0425	$[M-H-CH_3-H_2O]^-$	$C_{14}H_8O_4$	0.3	11.0
	239.0347	$[M-H-CH_3-H_3O]^-$	$C_{14}H_7O_4$	0.3	11.5
	230.0582	$[M-H-CH_3-CO]^-$	$C_{13}H_{10}O_4$	0.3	9.0
	229.0504	$[M-H-CH_3-CHO]^-$	$C_{13}H_9O_4$	0.3	9.5
	212.0477	$[M-H-CH_3-CH_2O_2]^-$	$C_{13}H_8O_3$	0.4	10.0
	211.0399	$[M-H-CH_3-CH_3O_2]^-$	$C_{13}H_7O_3$	0.4	10.5

Table S61. MS^3 fragmentation of m/z 287.0915 detected in the depolymerised Kraft lignin sample at retention time 2.94 min.

MS stage	m/z	Fragment-ion	Chemical formula	Mass difference in mDa	RDB
MS1	287.0915	$[M-H]^-$	$C_{16}H_{15}O_5$	-0.5	9.5
MS2	272.0687	$[M-H-CH_3]^-$	$C_{15}H_{12}O_5$	0.2	10.0
	136.0168	$[M-H-C_9H_{11}O_2]^-$	$C_7H_4O_3$	0.7	6.0
	123.0454	$[M-H-C_9H_8O_3]^-$	$C_7H_7O_2$	0.8	4.5
MS3 (272.0687)	257.0451	$[M-H-CH_3-CH_3]^-$	$C_{14}H_9O_5$	0.1	10.5
	254.0580	$[M-H-CH_3-H_2O]^-$	$C_{15}H_{10}O_4$	0.1	11.0
	243.0658	$[M-H-CH_3-CHO]^-$	$C_{14}H_{11}O_4$	0.1	9.5
	242.0580	$[M-H-CH_3-CH_2O]^-$	$C_{14}H_{10}O_4$	0.1	10.0
	151.0402	$[M-H-CH_3-C_7H_5O_2]^-$	$C_8H_7O_3$	0.6	5.5
	150.0323	$[M-H-CH_3-C_7H_6O_2]^-$	$C_8H_6O_3$	0.6	6.0
	137.0245	$[M-H-CH_3-C_8H_7O_2]^-$	$C_7H_5O_3$	0.7	5.5
	108.0219	$[M-H-CH_3-C_9H_8O_3]^-$	$C_6H_4O_2$	0.8	5.0

Table S62. MS^3 fragmentation of m/z 301.0706 detected in the depolymerised Kraft lignin sample at retention time 3.93 min.

MS stage	m/z	Fragment-ion	Chemical formula	Mass difference in mDa	RDB
MS1	301.0706	$[M-H]^-$	$C_{16}H_{13}O_6$	-0.6	10.5
MS2	286.0479	$[M-H-CH_3]^-$	$C_{15}H_{10}O_6$	0.1	11.0
MS3 (286.0479)	258.0530	$[M-H-CH_3-CO]^-$	$C_{14}H_{10}O_5$	0.2	10.0
	136.0167	$[M-H-CH_3-C_7H_6O_2]^-$	$C_7H_4O_3$	0.7	6.0

Table S63. MS^3 fragmentation of m/z 301.0707 detected in the depolymerised Kraft lignin sample at retention time 4.25 min.

MS stage	m/z	Fragment-ion	Chemical formula	Mass difference in mDa	RDB
MS1	301.0707	$[M-H]^-$	$C_{16}H_{13}O_6$	-0.5	10.5
MS2	286.0481	$[M-H-CH_3]^-$	$C_{15}H_{10}O_6$	0.4	11.0
	253.0508	$[M-H-CH_4O_2]^-$	$C_{15}H_9O_4$	0.8	11.5
MS3 (286.0481)	271.0243	$[M-H-CH_3-CH_3]^-$	$C_{14}H_7O_6$	0.1	11.5
	269.0451	$[M-H-CH_3-OH]^-$	$C_{15}H_9O_5$	0.1	11.5
	268.0373	$[M-H-CH_3-H_2O]^-$	$C_{15}H_8O_5$	0.2	12.0
	267.0452	$[M-H-CH_3-H_3O]^-$	$C_{15}H_7O_5$	0.3	12.5
	258.0530	$[M-H-CH_3-CO]^-$	$C_{14}H_{10}O_5$	0.2	10.0
	257.0452	$[M-H-CH_3-CHO]^-$	$C_{14}H_9O_5$	0.2	10.5
	256.0376	$[M-H-CH_3-CH_2O]^-$	$C_{14}H_8O_5$	-0.4	10.0
	240.0424	$[M-H-CH_3-CH_2O_2]^-$	$C_{14}H_8O_4$	0.2	11.0
	239.0346	$[M-H-CH_3-CH_3O_2]^-$	$C_{14}H_7O_4$	0.2	11.5
	229.0504	$[M-H-CH_3-C_2HO_2]^-$	$C_{13}H_9O_4$	0.3	9.5
	212.0477	$[M-H-CH_3-C_2H_2O_3]^-$	$C_{13}H_8O_3$	0.4	10.0
MS3 (253.0508)	238.0271	$[M-H-CH_4O_2-CH_3]^-$	$C_{14}H_6O_4$	0.5	12.0

Table S64. MS^3 fragmentation of m/z 301.1071 detected in the depolymerised Kraft lignin sample at retention time 2.99 min.

MS stage	m/z	Fragment-ion	Chemical formula	Mass difference in mDa	RDB
MS1	301.1071	$[M-H]^-$	$C_{17}H_{17}O_5$	-0.5	9.5
MS2	286.0482	$[M-H-CH_3]^-$	$C_{16}H_{14}O_5$	0.1	10.0
MS3 (286.0482)	271.0606	$[M-H-CH_3-CH_3]^-$	$C_{15}H_{11}O_5$	0.0	10.5
	270.0529	$[M-H-CH_3-CH_4]^-$	$C_{15}H_{10}O_5$	0.1	11.0
	269.0815	$[M-H-CH_3-HO]^-$	$C_{16}H_{13}O_4$	0.2	10.5
	268.0737	$[M-H-CH_3-H_2O]^-$	$C_{16}H_{12}O_4$	0.2	11.0
	267.0659	$[M-H-CH_3-H_3O]^-$	$C_{16}H_{11}O_4$	0.2	11.5
	257.0815	$[M-H-CH_3-CHO]^-$	$C_{15}H_{13}O_4$	0.1	9.5
	240.0788	$[M-H-CH_3-CH_2O_2]^-$	$C_{15}H_{12}O_3$	0.1	10.0
	239.0710	$[M-H-CH_3-CH_3O_2]^-$	$C_{15}H_{11}O_3$	0.2	10.5
	230.0581	$[M-H-CH_3-C_3H_4O]^-$	$C_{13}H_{10}O_4$	0.2	9.0
	177.0556	$[M-H-CH_3-C_6H_5O_2]^-$	$C_{10}H_9O_3$	0.5	6.5
	162.0323	$[M-H-CH_3-C_7H_8O_2]^-$	$C_9H_6O_3$	0.6	7.0
	150.0323	$[M-H-CH_3-C_8H_8O_2]^-$	$C_8H_6O_3$	0.6	6.0

Table S65. MS^3 fragmentation of m/z 313.1436 detected in the depolymerised Kraft lignin sample at retention time 5.18 min.

MS stage	m/z	Fragment-ion	Chemical formula	Mass difference in mDa	RDB
MS1	313.1436	$[M-H]^-$	$C_{19}H_{21}O_4$	-0.4	9.5
MS2	295.1337	$[M-H-H_2O]^-$	$C_{19}H_{19}O_3$	0.3	10.5
	265.1231	$[M-H-CH_4O_2]^-$	$C_{18}H_{17}O_2$	0.3	10.5
	235.1126	$[M-H-C_2H_6O_3]^-$	$C_{17}H_{15}O$	0.3	10.5
MS3 (295.1337)	277.1213	$[M-H-H_2O-H_2O]^-$	$C_{19}H_{17}O_2$	0.3	11.5
	224.0838	$[M-H-H_2O-C_4H_7O]^-$	$C_{15}H_{12}O_2$	0.1	10.0
MS3 (265.1231)	247.1124	$[M-H-CH_3-H_2O]^-$	$C_{18}H_{15}O$	0.1	11.5
	235.1126	$[M-H-CH_4O_2-CH_2O]^-$	$C_{17}H_{15}O$	0.3	10.5

Table S66. MS^3 fragmentation of m/z 313.1436 detected in the depolymerised Kraft lignin sample at retention time 5.75 min.

MS stage	m/z	Fragment-ion	Chemical formula	Mass difference in mDa	RDB
MS1	313.1436	$[M-H]^-$	$C_{19}H_{21}O_4$	-0.4	9.5
MS2	295.1337	$[M-H-H_2O]^-$	$C_{19}H_{19}O_3$	0.3	10.5
	269.1180	$[M-H-C_2H_4O]^-$	$C_{17}H_{17}O_3$	0.2	9.5
	211.0763	$[M-H-C_5H_{10}O_2]^-$	$C_{14}H_{11}O_2$	0.4	9.5
	199.0764	$[M-H-C_6H_{10}O_2]^-$	$C_{13}H_{11}O_2$	0.5	8.5
MS3 (265.1231)	254.0944	$[M-H-H_2O-C_3H_5]^-$	$C_{16}H_{14}O_3$	0.1	10.0
	251.1074	$[M-H-H_2O-C_2H_4O]^-$	$C_{17}H_{15}O_2$	0.2	10.5
	239.1075	$[M-H-H_2O-C_3H_4O]^-$	$C_{16}H_{15}O_2$	0.3	9.5
	225.0917	$[M-H-H_2O-C_4H_6O]^-$	$C_{15}H_{13}O_2$	0.1	9.5

Table S67. MS^3 fragmentation of m/z 315.0861 detected in the depolymerised Kraft lignin sample at retention time 4.00 min.

MS stage	m/z	Fragment-ion	Chemical formula	Mass difference in mDa	RDB
MS1	315.0861	$[M-H]^-$	$C_{17}H_{15}O_6$	-0.7	10.5
MS2	300.0706	$[M-H-CH_3]^-$	$C_{16}H_{12}O_6$	0.3	11.0
	267.0663	$[M-H-CH_4O_2]^-$	$C_{16}H_{11}O_4$	0.5	11.5
MS3 (300.0706)	285.0399	$[M-H-CH_3-CH_3]^-$	$C_{15}H_9O_6$	0.0	11.5
	283.0606	$[M-H-CH_3-OH]^-$	$C_{16}H_{11}O_5$	0.0	11.5
	282.0529	$[M-H-CH_3-H_2O]^-$	$C_{16}H_{10}O_5$	0.1	12.0
	281.0451	$[M-H-CH_3-H_3O]^-$	$C_{16}H_9O_5$	0.1	12.5
	272.0685	$[M-H-CH_3-CO]^-$	$C_{15}H_{12}O_5$	0.0	10.0
	271.0607	$[M-H-CH_3-CHO]^-$	$C_{15}H_{11}O_5$	0.1	10.5
	270.0530	$[M-H-CH_3-CH_2O]^-$	$C_{15}H_{11}O_5$	0.1	10.5
	258.0528	$[M-H-CH_3-C_2H_2O]^-$	$C_{14}H_{10}O_5$	0.0	10.0
	257.0451	$[M-H-CH_3-C_2H_3O]^-$	$C_{14}H_9O_5$	0.1	10.5
	254.0580	$[M-H-CH_3-CH_2O_2]^-$	$C_{15}H_{10}O_4$	0.1	11.0
	253.0503	$[M-H-CH_3-CH_3O_2]^-$	$C_{15}H_9O_4$	0.2	11.5
	243.0658	$[M-H-CH_3-C_2HO_2]^-$	$C_{14}H_{11}O_4$	0.1	9.5
	229.0503	$[M-H-CH_3-C_3H_3O_2]^-$	$C_{13}H_9O_4$	0.3	9.5
	226.0632	$[M-H-CH_3-C_2H_2O_3]^-$	$C_{14}H_{10}O_3$	0.2	10.0
MS3 (267.0663)	252.0425	$[M-H-CH_4O_2-CH_3]^-$	$C_{15}H_8O_4$	0.3	12.0

Table S68. MS^3 fragmentation of m/z 315.1227 detected in the depolymerised Kraft lignin sample at retention time 3.13 min.

MS stage	m/z	Fragment-ion	Chemical formula	Mass difference in mDa	RDB
MS1	315.1227	$[M-H]^-$	$C_{18}H_{19}O_5$	-0.5	9.5
MS2	300.0999	$[M-H-CH_3]^-$	$C_{17}H_{16}O_5$	0.1	10.0
	178.0636	$[M-H-C_8H_9O_2]^-$	$C_{10}H_{10}O_3$	0.6	6.0
MS3 (300.0999)	285.0765	$[M-H-CH_3-CH_3]^-$	$C_{16}H_{13}O_5$	0.2	10.5
	257.0816	$[M-H-CH_3-C_2H_3O]^-$	$C_{15}H_{13}O_4$	0.3	9.5
	164.0479	$[M-H-CH_3-C_8H_8O_2]^-$	$C_9H_8O_3$	0.5	6.0

Table S69. MS^2fragmentation of m/z 317.1022 detected in the depolymerised Kraft lignin sample at retention time 4.50 min.

MS stage	m/z	Fragment-ion	Chemical formula	Mass difference in mDa	RDB
MS1	317.1022	$[M-H]^-$	$C_{17}H_{17}O_6$	-0.3	9.5
MS2	302.0794	$[M-H-CH_3]^-$	$C_{16}H_{14}O_6$	0.3	10.0
	299.0923	$[M-H-H_2O]^-$	$C_{17}H_{15}O_5$	0.3	10.5
	287.0924	$[M-H-CH_2O]^-$	$C_{16}H_{15}O_5$	0.5	9.5
	273.1132	$[M-H-CO_2]^-$	$C_{16}H_{17}O_4$	0.5	8.5
	193.0506	$[M-H-C_7H_8O_2]^-$	$C_{10}H_9O_4$	0.6	6.5

Table S70. MS^2 fragmentation of m/z 329.1017 detected in the depolymerised Kraft lignin sample at retention time 3.80 min.

MS stage	m/z	Fragment-ion	Chemical formula	Mass difference in mDa	RDB
MS1	329.1017	$[M-H]^-$	$C_{18}H_{17}O_6$	-0.8	10.5
MS2	314.0791	$[M-H-CH_3]^-$	$C_{17}H_{14}O_6$	0.1	11.0
	205.0506	$[M-H-C_7H_8O_2]^-$	$C_{11}H_9O_4$	0.5	7.5

Table S71. MS^2 fragmentation of m/z 329.1016 detected in the depolymerised Kraft lignin sample at retention time 3.87 min.

MS stage	m/z	Fragment-ion	Chemical formula	Mass difference in mDa	RDB
MS1	329.1016	$[M-H]^-$	$C_{18}H_{17}O_6$	-0.9	10.5
MS2	314.0790	$[M-H-CH_3]^-$	$C_{17}H_{14}O_6$	0.1	11.0

Table S72. MS^2 fragmentation of m/z 329.1019 detected in the depolymerised Kraft lignin sample at retention time 4.16 min.

MS stage	m/z	Fragment-ion	Chemical formula	Mass difference in mDa	RDB
MS1	329.1019	$[M-H]^-$	$C_{18}H_{17}O_6$	-0.6	10.5
MS2	314.0790	$[M-H-CH_3]^-$	$C_{17}H_{14}O_6$	0.1	11.0

Table S73. MS^3 fragmentation of m/z 329.1382 detected in the depolymerised Kraft lignin sample at retention time 6.18 min.

MS stage	m/z	Fragment-ion	Chemical formula	Mass difference in mDa	RDB
MS1	329.1382	$[M-H]^-$	$C_{19}H_{21}O_5$	-0.7	9.5
MS2	311.1285	$[M-H-H_2O]^-$	$C_{19}H_{19}O_4$	0.2	10.5
MS3 (311.1285)	293.1180	$[M-H-H_2O-H_2O]^-$	$C_{19}H_{17}O_3$	0.2	11.5
	267.1023	$[M-H-H_2O-C_2H_4O]^-$	$C_{17}H_{15}O_3$	0.1	10.5
	253.0868	$[M-H-H_2O-C_3H_6O]^-$	$C_{16}H_{13}O_3$	0.3	10.5
	223.0761	$[M-H-H_2O-C_4H_8O_2]^-$	$C_{15}H_{11}O_2$	0.2	10.5
	211.0761	$[M-H-H_2O-C_5H_8O_2]^-$	$C_{14}H_{11}O_2$	0.2	9.5
	199.0763	$[M-H-H_2O-C_6H_8O_2]^-$	$C_{13}H_{11}O_2$	0.4	8.5

Table S74. MS^3 fragmentation of m/z 331.1174 detected in the depolymerised Kraft lignin sample at retention time 4.69 min.

MS stage	m/z	Fragment-ion	Chemical formula	Mass difference in mDa	RDB
MS1	331.1174	$[M-H]^-$	$C_{18}H_{19}O_6$	-0.7	9.5
MS2	316.0948	$[M-H-CH_3]^-$	$C_{17}H_{16}O_6$	0.1	10.0
	285.0766	$[M-H-C_2H_6O]^-$	$C_{16}H_{13}O_5$	0.3	10.5
	165.0558	$[M-H-C_9H_{10}O_3]^-$	$C_9H_9O_3$	0.7	5.5
MS3 (316.0948)	271.0609	$[M-H-CH_3-C_2H_5O]^-$	$C_{15}H_{11}O_5$	0.3	10.5
MS3 (285.0766)	270.0531	$[M-H-C_2H_6O-CH_3]^-$	$C_{15}H_{10}O_5$	0.3	11.0
	241.0504	$[M-H-C_2H_6O-C_2H_4O]^-$	$C_{14}H_9O_4$	0.4	10.5

Table S75. MS^3 fragmentation of m/z 345.1333 detected in the depolymerised Kraft lignin sample at retention time 4.61 min. Identified as fragment of 419.1700.

MS stage	m/z	Fragment-ion	Chemical formula	Mass difference in mDa	RDB
MS1	345.1333	$[M-H]^-$	$C_{19}H_{21}O_6$	-0.5	9.5
MS2	330.1106	$[M-H-CH_3]^-$	$C_{18}H_{18}O_6$	0.3	10.0
	327.1239	$[M-H-H_2O]^-$	$C_{19}H_{19}O_5$	0.2	10.5
	312.1002	$[M-H-CH_5O]^-$	$C_{18}H_{16}O_5$	0.4	11.0
MS3 (330.1106)	315.0868	$[M-H-CH_3-CH_3]^-$	$C_{17}H_{15}O_6$	-0.1	10.5
	312.1002	$[M-H-CH_3-H_2O]^-$	$C_{18}H_{16}O_5$	0.4	11.0
	301.1076	$[M-H-CH_3-CHO]^-$	$C_{17}H_{17}O_5$	0.3	11.0
	300.1003	$[M-H-CH_3-CH_2O]^-$	$C_{17}H_{16}O_5$	-0.4	9.0
	299.0920	$[M-H-CH_3-CH_3O]^-$	$C_{17}H_{15}O_5$	0.1	10.5
	285.0763	$[M-H-CH_3-C_2H_5O]^-$	$C_{16}H_{13}O_5$	0.0	10.5
	207.0660	$[M-H-CH_3-C_7H_7O_2]^-$	$C_{11}H_{11}O_4$	0.3	6.5
	193.0504	$[M-H-CH_3-C_8H_9O_2]^-$	$C_{10}H_9O_4$	0.4	6.5
	165.0557	$[M-H-CH_3-C_9H_9O_3]^-$	$C_9H_9O_3$	0.5	5.5
	150.0325	$[M-H-CH_3-C_{10}H_{12}O_3]^-$	$C_8H_6O_3$	0.8	6.0
	136.0167	$[M-H-CH_3-C_{11}H_{14}O_3]^-$	$C_7H_4O_3$	0.7	6.0
	122.0375	$[M-H-CH_3-C_{11}H_{12}O_4]^-$	$C_8H_6O_3$	0.7	5.0
	108.0220	$[M-H-CH_3-C_{12}H_{14}O_4]^-$	$C_6H_4O_2$	0.8	5.0

Table S76. MS^3 fragmentation of m/z 345.1333 detected in the depolymerised Kraft lignin sample at retention time 6.86 min.

MS stage	m/z	Fragment-ion	Chemical formula	Mass difference in mDa	RDB
MS1	345.1333	$[M-H]^-$	$C_{19}H_{21}O_6$	-0.5	9.5
MS2	327.1232	$[M-H-H_2O]^-$	$C_{19}H_{19}O_5$	0.0	10.5
	271.0871	$[M-H-C_3H_7O_2]^-$	$C_{16}H_{15}O_4$	0.1	9.5
	241.0867	$[M-H-C_4H_8O_3]^-$	$C_{15}H_{13}O_3$	0.2	9.5
	199.0764	$[M-H-C_6H_{10}O_4]^-$	$C_{13}H_{15}O_2$	0.5	8.5
MS3 (327.1232)	267.1023	$[M-H-H_2O-C_2H_4O_2]^-$	$C_{17}H_{15}O_3$	0.1	10.5
	211.0762	$[M-H-H_2O-C_5H_8O_3]^-$	$C_{14}H_{11}O_2$	0.3	9.5

Table S77. MS^3 fragmentation of m/z 347.1123 detected in the depolymerised Kraft lignin sample at retention time 5.29 min.

MS stage	m/z	Fragment-ion	Chemical formula	Mass difference in mDa	RDB
MS1	347.1123	$[M-H]^-$	$C_{18}H_{19}O_7$	-0.8	9.5
MS2	329.1025	$[M-H-H_2O]^-$	$C_{18}H_{17}O_6$	-0.3	10.5
	287.0920	$[M-H-C_2H_4O_2]^-$	$C_{16}H_{15}O_5$	0.1	9.5
MS3 (329.1025)	314.0791	$[M-H-H_2O-CH_3]^-$	$C_{17}H_{14}O_6$	0.2	11.0
	286.0843	$[M-H-H_2O-C_2H_3O]^-$	$C_{16}H_{14}O_5$	0.2	10.0
	285.0765	$[M-H-H_2O-C_2H_4O]^-$	$C_{16}H_{13}O_5$	0.2	10.5
	271.0609	$[M-H-H_2O-C_3H_6O]^-$	$C_{15}H_{11}O_5$	0.2	10.5

Table S78. MS^3 fragmentation of m/z 359.1491 detected in the depolymerised Kraft lignin sample at retention time 4.47 min.

MS stage	m/z	Fragment-ion	Chemical formula	Mass difference in mDa	RDB
MS1	359.1491	$[M-H]^-$	$C_{20}H_{23}O_6$	-0.4	9.5
MS2	344.1259	$[M-H-CH_3]^-$	$C_{19}H_{20}O_6$	-0.1	10.0
	341.1392	$[M-H-H_2O]^-$	$C_{20}H_{21}O_5$	0.3	10.5
MS3 (344.1259)	329.1026	$[M-H-CH_3-CH_3]^-$	$C_{18}H_{17}O_6$	0.1	10.5
	315.1234	$[M-H-CH_3-CHO]^-$	$C_{18}H_{19}O_5$	0.2	9.5
	314.1155	$[M-H-CH_3-CH_2O]^-$	$C_{18}H_{18}O_5$	0.1	10.0
	313.1078	$[M-H-CH_3-CH_3O]^-$	$C_{18}H_{17}O_5$	0.2	10.5
	288.1000	$[M-H-CH_3-C_3H_4O]^-$	$C_{16}H_{16}O_5$	0.3	9.0
	163.0401	$[M-H-CH_3-C_{10}H_{13}O_3]^-$	$C_9H_7O_3$	0.6	6.5
	150.0323	$[M-H-CH_3-C_{11}H_{14}O_3]^-$	$C_8H_6O_3$	0.6	6.0

Table S79. Reproducibility of the retention time of vanillin.

Injection	Retention time in min
1	1.28
2	1.27
3	1.26
4	1.27
5	1.27
6	1.24
7	1.27
8	1.26
9	1.27
10	1.26
11	1.25
12	1.27
13	1.25
14	1.27
15	1.26
Average	1.26
Absolute standard deviation	0.01

Table S80. Peak list creation workflow using MZmine 2.

1. Raw data methods/Peak detection

1.1 Mass detection

Settings:

- Polarity: negative
- Mass detector: centroid
- Spectrum type: any
- Noise level: 1.0E4

1.2 FTMS shoulder peaks filter

Settings:

- Resolution: 60,000
- Peak model function: Lorentzian extended

1.3 Chromatogram builder

Settings:

- MS level: 1
- Polarity: negative
- Spectrum type: any
- Min time span: 0.03 min
- Min height: 5.0E4
- m/z tolerance: 0.005 Da or 5.0 ppm

2. Peak list methods

2.1 Peak detection/chromatogram deconvolution

Settings:

- Algorithm: local minimum search
- Chromatographic threshold: 30.0%
- Minimum relative height: 1.0%
- Minimum absolute height: 1.0E4
- Min ratio of peak top/edge 2
- m/z center calculation: median

2.2 Isotopes/isotopes peak grouper

Settings:

- m/z tolerance: 0.01 Da or 10.0 ppm
- Retention time tolerance: 0.05 min
- Maximum charge: 1
- Representative isotope: most intense

2.3 Identification/adduct search

Settings:

- Retention time tolerance: 0.05 min
- Adducts: [M+NH3], 17.0265 Da; [M+H3PO4], 97.9769 Da; [M+H2SO4], 97.9674; [M+CH2O2], 46.0056
- m/z tolerance: 0.01 Da or 5 ppm
- Max relative adduct peak height: 10.0%

2.4 Identification/formula prediction

Settings:

- Charge: 1
- Ionisation type: $[M-H]^-$
- m/z tolerance: 0.005 Da or 10.0 ppm
- Max best formulas per peak: 3
- Elements: C, O, H, S (for all min: 0, max: 100)
- Element count heuristics: H/C ratio: yes, multiple element counts: yes
- RDBE restrictions: RDBE range: -1 to 40, RDBE must be an integer: yes

Table S81. Suspect list of lignin-related phenolic compounds identified in literature including exact mass of neutral compound, exact mass of deprotonated compounds, exact mass of protonated compound, chemical formula of neutral compound, ring double bond equivalent (RDB), type pf lignin compound, compound label, reference and compound name.

Exact mass	$[M-H]^-$	$[M+H]^+$	Chemical formula (neutral compound)	RDB	Type of lignin compound	Compound label	Reference	Compound name
94.0414	93.0341	95.0487	C6 H6 O1	4	Monomer	M1		Phenol
108.0570	107.0497	109.0643	C7 H8 O1	4	Monomer	M2		o-Cresol
108.0570	107.0497	109.0643	C7 H8 O1	4	Monomer	M3		p-Cresol
110.0363	109.0290	111.0436	C6 H6 O2	4	Monomer	M4		Catechol
122.0363	121.0290	123.0436	C7 H6 O2	5	Monomer	M5		Benzoic acid
122.0363	121.0290	123.0436	C7 H6 O2	5	Monomer	M6		p-Hydroxy-benzaldehyde
122.0727	121.0654	123.0800	C8 H10 O1	4	Monomer	M7		2,4-Dimethylphenol
124.0519	123.0446	125.0592	C7 H8 O2	4	Monomer	M8		4-hydroxybenzyl alcohol
124.0519	123.0446	125.0592	C7 H8 O2	4	Monomer	M9		Guaiacol
136.0519	135.0446	137.0592	C8 H8 O2	5	Monomer	M10		p-Hydroxy-acetophenone
138.0312	137.0239	139.0385	C7 H6 O3	5	Monomer	M11		p-Hydroxy-benzoic acid
138.0676	137.0603	139.0749	C8 H10 O2	4	Monomer	M12		2-(4-hydroxyphenyl) ethanol
148.0519	147.0446	149.0592	C9 H8 O2	6	Monomer	M13		4-hydroxycinnamaldehyde
148.0519	147.0446	149.0592	C9 H8 O2	6	Monomer	M14		Cinnamic acid
150.0676	149.0603	151.0749	C9 H10 O2	5	Monomer	M15		p-coumaryl alcohol
152.0468	151.0395	153.0541	C8 H8 O3	5	Monomer	M16		Vanillin
152.0468	151.0395	153.0541	C8 H8 O3	5	Monomer	M17		4-methoxybenzoic acid
154.0261	153.0188	155.0334	C7 H6 O4	5	Monomer	M18		3,4-Dihydroxybenzoic acid
154.0261	153.0188	155.0334	C7 H6 O4	5	Monomer	M19		3,5-Dihydroxybenzoic acid
154.0625	153.0552	155.0698	C8 H10 O3	4	Monomer	M20		Syringol
154.0625	153.0552	155.0698	C8 H10 O3	4	Monomer	M21		Vanillyl alcohol
164.0468	163.0395	165.0541	C9 H8 O3	6	Monomer	M22		p-Coumaric acid
164.0832	163.0759	165.0905	C10 H12 O2	5	Monomer	M23		Eugenol

164.0832	163.0759	165.0905	C10 H12 O2	5	Monomer	M24		Iso-eugenol
166.0625	165.0552	167.0698	C9 H10 O3	5	Monomer	M25		Veratraldehyde
166.0625	165.0552	167.0698	C9 H10 O3	5	Monomer	M26		Acetovanillone
168.0418	167.0345	169.0491	C8 H8 O4	5	Monomer	M27		Vanillic acid
168.0418	167.0345	169.0491	C8 H8 O4	5	Monomer	M28		3,4-Dihydroxyphenylacetic acid
178.0625	177.0552	179.0698	C10 H10 O3	6	Monomer	M29		Coniferyl aldehyde
178.0625	177.0552	179.0698	C10 H10 O3	6	Monomer	M30		3-methoxycinnamic acid
178.0625	177.0552	179.0698	C10 H10 O3	6	Monomer	M31		4-methoxycinnamic acid
180.0418	179.0345	181.0491	C9 H8 O4	6	Monomer	M32		3,4-dihydroxycinnamic acid
180.0781	179.0708	181.0854	C10 H12 O3	5	Monomer	M33		Coniferyl alcohol
182.0574	181.0501	183.0647	C9 H10 O4	5	Monomer	M34		Syringaldehyde
182.0574	181.0501	183.0647	C9 H10 O4	5	Monomer	M35		3,4-Dihydroxyhydrocinnamic acid
194.0574	193.0501	195.0647	C10 H10 O4	6	Monomer	M36		Ferulic acid
196.0731	195.0658	197.0804	C10 H12 O4	5	Monomer	M37		Acetosyringone
198.0523	197.0450	199.0596	C9 H10 O5	5	Monomer	M38		Syringic acid
208.0731	207.0658	209.0804	C11 H12 O4	6	Monomer	M39		3,5-Dimethoxycinnamic acid
208.0731	207.0658	209.0804	C11 H12 O4	6	Monomer	M40		Sinapaldehyde
208.0731	207.0658	209.0804	C11 H12 O4	6	Monomer	M41		3,4-Dimethoxycinnamic acid
210.0887	209.0814	211.0960	C11 H14 O4	5	Monomer	M42		Sinapyl alcohol
224.0680	223.0607	225.0753	C11 H12 O5	6	Monomer	M43		3,5-dimethoxy-4-hydroxycinnamic acid
244.0737	243.0664	245.0810	C14 H12 O4	9	Dimer	D35	Prothmann et al.	
246.0892	245.0819	247.0965	C14 H18 O4	8	Dimer	D36	Prothmann et al.	
258.0892	257.0819	259.0965	C15 H14 O4	9	Dimer	D37	Prothmann et al.	
260.1044	259.0971	261.1117	C15 H16 O4	8	Dimer	D38	Prothmann et al.	
270.0889	269.0816	271.0962	C16 H14 O4	10	Dimer	D39	Prothmann et al.	
272.1048	271.0975	273.1121	C16 H16 O4	9	Dimer	D40	Prothmann et al.	
274.0841	273.0768	275.0914	C15 H14 O5	9	Dimer	D41	Prothmann et al.	

274.0845	273.0772	275.0918	C15 H14 O5	9	Dimer	D30	Jarrell et al.	
274.1201	273.1128	275.1274	C16 H18 O4	8	Dimer	D42	Prothmann et al.	
288.0999	287.0926	289.1072	C16 H16 O5	9	Dimer	D43	Prothmann et al.	
298.1200	297.1127	299.1273	C18 H18 O4	10	Dimer	D1	Kiyota et al.	
298.1200	297.1127	299.1273	C18 H18 O5	10	Dimer	D2	Kiyota et al.	
300.0992	299.0919	301.1065	C17 H16 O5	10	Dimer	D3	Banoub et al.	
302.0790	301.0717	303.0863	C16 H14 O6	10	Dimer	D44	Prothmann et al.	
304.0950	303.0877	305.1023	C16 H16 O6	9	Dimer	D29	Jarrell et al.	
312.0992	311.0919	313.1065	C18 H16 O5	11	Dimer	D4	Banoub et al.	
314.1152	313.1079	315.1225	C18 H18 O5	10	Dimer	D45	Prothmann et al.	
316.1309	315.1236	317.1382	C18 H20 O5	9	Dimer	D46	Prothmann et al.	
322.1204	321.1131	323.1277	C20 H18 O4	12	Dimer	D47	Prothmann et al.	
326.1149	325.1076	327.1222	C19 H18 O5	11	Dimer	D5	Banoub et al.	
328.1306	327.1233	329.1379	C19 H20 O5	10	Dimer	D6	Kiyota et al.	
330.0741	329.0668	331.0814	C17 H14 O7	11	Dimer	D34	Jarrell et al.	
330.1098	329.1025	331.1171	C18 H18 O6	10	Dimer	D7	Banoub et al.	
332.1261	331.1188	333.1334	C18 H20 O6	9	Dimer	D31	Jarrell et al.	
336.0995	335.0922	337.1068	C20 H16 O5	13	Dimer	D48	Prothmann et al.	
336.1263	335.1190	337.1336	C20 H20 O6	11	Dimer	D32	Jarrell et al.	
340.0942	339.0869	341.1015	C19 H16 O6	12	Dimer	D8	Banoub et al.	
340.1305	339.1232	341.1378	C20 H20 O5	11	Dimer	D9	Banoub et al.	
342.1098	341.1025	343.1171	C19 H18 O6	11	Dimer	D10	Banoub et al.	
344.1255	343.1182	345.1328	C19 H20 O6	10	Dimer	D11	Banoub et al.	
350.0786	349.0713	351.0859	C20 H14 O6	14	Dimer	D12	Banoub et al.	
350.1150	349.1077	351.1223	C21 H18 O5	13	Dimer	D49	Prothmann et al.	
352.0942	351.0869	353.1015	C20 H16 O6	13	Dimer	D13	Banoub et al.	
352.1308	351.1235	353.1381	C21 H20 O5	12	Dimer	D50	Prothmann et al.	
354.1464	353.1391	355.1537	C21 H22 O5	11	Dimer	D51	Prothmann et al.	
358.1411	357.1338	359.1484	C20 H22 O6	10	Dimer	D14	Kiyota et al.	

358.1411	357.1338	359.1484	C20 H22 O6	10	Dimer	D15	Kiyota et al.	
362.1725	361.1652	363.1798	C20 H26 O6	8	Dimer	D52	Prothmann et al.	
363.1075	362.1002	364.1148	C18 H19 O8	9.5	Dimer	D16	Banoub et al.	
368.1255	367.1182	369.1328	C21 H20 O6	12	Dimer	D17	Banoub et al.	
372.1212	371.1139	373.1285	C20 H20 O7	9	Dimer	D33	Jarrell et al.	
376.1517	375.1444	377.1590	C20 H24 O7	9	Dimer	D18	Kiyota et al.	
378.1460	377.1387	379.1533	C23 H22 O5	13	Dimer	D54	Prothmann et al.	
380.1466	379.1393	381.1539	C19 H24 O8	7	Dimer	D58	Albishi et al.	
384.1204	383.1131	385.1277	C21 H20 O7	12	Dimer	D19	Banoub et al.	
386.1361	385.1288	387.1434	C21 H22 O7	11	Dimer	D20	Banoub et al.	
388.1517	387.1444	389.1590	C21 H24 O7	10	Dimer	D21	Kiyota et al.	
388.1517	387.1444	389.1590	C21 H24 O7	10	Dimer	D22	Kiyota et al.	
390.1321	389.1248	391.1394	C20 H22 O8	10	Dimer	D23	Huis et al.	
394.1405	393.1332	395.1478	C23 H22 O6	13	Dimer	D55	Prothmann et al.	
396.1205	395.1132	397.1278	C22 H20 O7	13	Dimer	D53	Prothmann et al.	
396.1567	395.1494	397.1640	C23 H24 O6	12	Dimer	D56	Prothmann et al.	
402.1309	401.1236	403.1382	C21 H22 O8	11	Dimer	D24	Banoub et al.	
406.1623	405.1550	407.1696	C21 H26 O8	9	Dimer	D25	Kiyota et al.	
418.1623	417.1550	419.1696	C22 H26 O8	10	Dimer	D26	Kiyota et al.	
420.1568	419.1495	421.1641	C25 H24 O6	14	Trimer	TR35	Prothmann et al.	
422.1362	421.1289	423.1435	C24 H22 O7	14	Dimer	D57	Prothmann et al.	
432.1204	431.1131	433.1277	C25 H20 O7	16	Trimer	TR1	Banoub et al.	
436.1728	435.1655	437.1801	C22 H28 O9	9	Dimer	D27	Kiyota et al.	
452.1829	451.1756	453.1902	C26 H28 O7	13	Trimer	TR36	Prothmann et al.	
454.1986	453.1913	455.2059	C26 H30 O7	12	Trimer	TR2	Banoub et al.	
464.1829	463.1756	465.1902	C27 H28 O7	14	Trimer	TR37	Prothmann et al.	
480.1784	479.1711	481.1857	C27 H28 O8	14	Trimer	TR38	Prothmann et al.	
483.2014	482.1941	484.2087	C27 H31 O8	12.5	Trimer	TR3	Banoub et al.	
484.2083	483.2010	485.2156	C27 H32 O8	12	Trimer	TR39	Prothmann et al.	

490.1622	489.1549	491.1695	C28 H26 O8	16	Trimer	TR4	Banoub et al.	
492.1779	491.1706	493.1852	C28 H28 O8	15	Trimer	TR5	Banoub et al.	
494.1936	493.1863	495.2009	C28 H30 O8	14	Trimer	TR6	Kiyota et al.	
508.1728	507.1655	509.1801	C28 H28 O9	15	Trimer	TR7	Banoub et al.	
510.2239	509.2166	511.2312	C29 H34 O8	13	Trimer	TR40	Prothmann et al.	
522.1885	521.1812	523.1958	C29 H30 O9	15	Trimer	TR8	Banoub et al.	
536.2041	535.1968	537.2114	C30 H32 O9	15	Trimer	TR9	Kiyota et al.	
537.2119	536.2046	538.2192	C30 H33 O9	14.5	Trimer	TR10	Banoub et al.	
550.1834	549.1761	551.1907	C30 H30 O10	16	Trimer	TR11	Banoub et al.	
552.1866	551.1793	553.1939	C26 H32 O13	11	Dimer	D28	Huis et al.	
552.2009	551.1936	553.2082	C30 H32 O10	15	Trimer	TR12	Huis et al.	
554.2147	553.2074	555.2220	C30 H34 O10	14	Trimer	TR13	Kiyota et al.	
556.2319	555.2246	557.2392	C30 H36 O10	13	Trimer	TR14	Huis et al.	
558.2475	557.2402	559.2548	C30 H38 O10	12	Trimer	TR15	Huis et al.	
568.1938	567.1865	569.2011	C30 H32 O11	15	Trimer	TR16	Banoub et al.	
570.2120	569.2047	571.2193	C30 H34 O11	14	Trimer	TR17	Huis et al.	
572.2253	571.2180	573.2326	C30 H36 O11	13	Trimer	TR18	Kiyota et al.	
578.1783	577.1710	579.1856	C31 H30 O11	17	Trimer	TR19	Banoub et al.	
580.1940	579.1867	581.2013	C31 H32 O11	16	Trimer	TR20	Banoub et al.	
582.2107	581.2034	583.2180	C31 H34 O11	15	Trimer	TR21	Morreel et al.	
584.2253	583.2180	585.2326	C31 H36 O11	14	Trimer	TR22	Kiyota et al.	
584.2253	583.2180	585.2326	C31 H36 O11	14	Trimer	TR23	Kiyota et al.	
586.2421	585.2348	587.2494	C31 H38 O11	13	Trimer	TR24	Morreel et al.	
602.2358	601.2285	603.2431	C31 H38 O12	13	Trimer	TR25	Kiyota et al.	
606.2249	605.2176	607.2322	C37 H34 O8	21	Tetramer	TE1	Banoub et al.	
614.2367	613.2294	615.2440	C32 H38 O12	14	Trimer	TR26	Morreel et al.	
614.2371	613.2298	615.2444	C32 H38 O12	14	Trimer	TR27	Morreel et al.	
614.2372	613.2299	615.2445	C32 H38 O12	14	Trimer	TR28	Morreel et al.	
628.2303	627.2230	629.2376	C36 H36 O10	19	Tetramer	TE13	Prothmann et al.	

628.2526	627.2453	629.2599	C33 H40 O12	14	Trimer	TR29	Morreel et al.	
630.2320	629.2247	631.2393	C32 H38 O13	14	Trimer	TR30	Morreel et al.	
632.2464	631.2391	633.2537	C32 H40 O13	13	Trimer	TR31	Kiyota et al.	
632.2612	631.2539	633.2685	C36 H40 O10	17	Tetramer	TE14	Prothmann et al.	
636.1991	635.1918	637.2064	C37 H32 O10	22	Tetramer	TE2	Banoub et al.	
644.2473	643.2400	645.2546	C33 H40 O13	14	Trimer	TR32	Morreel et al.	
654.2460	653.2387	655.2533	C38 H38 O10	20	Tetramer	TE3	Banoub et al.	
660.2418	659.2345	661.2491	C33 H40 O14	14	Trimer	TR33	Morreel et al.	
662.2570	661.2497	663.2643	C33 H42 O14	13	Trimer	TR34	Kiyota et al.	
684.2202	683.2129	685.2275	C38 H36 O12	21	Tetramer	TE4	Banoub et al.	
748.2745	747.2672	749.2818	C40 H44 O14	19	Tetramer	TE5	Huis et al.	
751.2597	750.2524	752.2670	C39 H43 O15	17	Tetramer	TE15	Albishi et al.	
756.2413	755.2340	757.2486	C41 H40 O14	22	Tetramer	TE6	Banoub et al.	
778.2841	777.2768	779.2914	C41 H46 O15	19	Tetramer	TE7	Morreel et al.	
779.2546	778.2473	780.2619	C40 H43 O16	18	Tetramer	TE16	Albishi et al.	
780.2999	779.2926	781.3072	C41 H48 O15	18	Tetramer	TE8	Morreel et al.	
788.2828	787.2755	789.2901	C46 H44 O12	25	Pentamer	P1	Banoub et al.	
810.3101	809.3028	811.3174	C42 H50 O16	18	Tetramer	TE9	Morreel et al.	
810.3102	809.3029	811.3175	C42 H50 O16	18	Tetramer	TE10	Morreel et al.	
826.3060	825.2987	827.3133	C42 H50 O17	18	Tetramer	TE11	Morreel et al.	
830.2570	829.2497	831.2643	C47 H42 O14	27	Pentamer	P2	Banoub et al.	
840.3219	839.3146	841.3292	C43 H52 O17	18	Tetramer	TE12	Morreel et al.	
860.3039	859.2966	861.3112	C49 H48 O14	26	Pentamer	P3	Banoub et al.	
876.2988	875.2915	877.3061	C49 H48 O15	26	Pentamer	P4	Banoub et al.	
890.3145	889.3072	891.3218	C50 H50 O15	26	Pentamer	P5	Banoub et al.	
906.2883	905.2810	907.2956	C53 H46 O14	31	Hexamer	HEX1	Banoub et al.	
950.3145	949.3072	951.3218	C55 H50 O15	31	Hexamer	HEX2	Banoub et al.	
956.3098	955.3025	957.3171	C50 H52 O19	24	Pentamer	P6	Albishi et al.	
1038.3305	1037.3232	1039.3378	C58 H54 O18	31	Hexamer	HEX4	Albishi et al.	

1054.3254	1053.3181	1055.3327	C58 H54 O19	31	Hexamer	HEX5	Albishi et al.	
1082.3720	1081.3647	1083.3793	C64 H58 O16	36	Heptamer	HEP1	Banoub et al.	
1098.3669	1097.3596	1099.3742	C64 H58 O17	36	Heptamer	HEP2	Banoub et al.	
1200.4045	1199.3972	1201.4118	C61 H68 O25	27	Hexamer	HEX6	Albishi et al.	
1216.3995	1215.3922	1217.4068	C61 H68 O26	27	Hexamer	HEX7	Albishi et al.	
1232.4657	1231.4584	1233.4730	C63 H76 O25	26	Hexamer	HEX3	Morreel et al.	

Table S82. Variables used for the four KMD-PCA-QDA classification models including the number of C-atoms (#C), number of H-atoms (#H), the number of O-atoms (#O) and the calculated KMDs for phenol (C_6H_5O), methoxy/primary alcohol (CH_3O), carboxylic acid (CHO_2), aldehyde (CHO) and secondary alcohol (CH_2O).

Exact mass	#C	#H	#O	Kendrick mass defect				
				Phenol	Methoxy/primary alcohol	Carboxylic acid	Aldehyde	Secondary alcohol
94.0414	6	6	1	-0.006991	0.014355	-0.046301	-0.032516	-0.008293
108.0570	7	8	1	-0.017463	0.007064	-0.062631	-0.046791	-0.018959
108.0570	7	8	1	-0.017463	0.007064	-0.062631	-0.046791	-0.018959
110.0363	6	6	2	0.003961	0.028938	-0.042034	-0.025904	0.002437
122.0363	7	6	2	0.008352	0.036052	-0.042660	-0.024771	0.006662
122.0363	7	6	2	0.008352	0.036052	-0.042660	-0.024771	0.006662
122.0727	8	10	1	-0.028035	-0.000326	-0.079062	-0.061167	-0.029725
124.0519	7	8	2	-0.006511	0.021647	-0.058365	-0.040180	-0.008228
124.0519	7	8	2	-0.006511	0.021647	-0.058365	-0.040180	-0.008228
136.0519	8	8	2	-0.002120	0.028762	-0.058990	-0.039047	-0.004004
138.0312	7	6	3	0.019304	0.050635	-0.038393	-0.018160	0.017393
138.0676	8	10	2	-0.017083	0.014257	-0.074795	-0.054556	-0.018994
148.0519	9	8	2	0.002270	0.035876	-0.059616	-0.037913	0.000221
148.0519	9	8	2	0.002270	0.035876	-0.059616	-0.037913	0.000221
150.0676	9	10	2	-0.012692	0.021371	-0.075421	-0.053423	-0.014770
152.0468	8	8	3	0.008832	0.043345	-0.054724	-0.032436	0.006727
152.0468	8	8	3	0.008832	0.043345	-0.054724	-0.032436	0.006727
154.0261	7	6	4	0.030256	0.065218	-0.034127	-0.011549	0.028124
154.0261	7	6	4	0.030256	0.065218	-0.034127	-0.011549	0.028124
154.0625	8	10	3	-0.006130	0.028840	-0.070529	-0.047945	-0.008263
154.0625	8	10	3	-0.006130	0.028840	-0.070529	-0.047945	-0.008263
164.0468	9	8	3	0.013223	0.050459	-0.055349	-0.031302	0.010951
164.0832	10	12	2	-0.023164	0.014081	-0.091751	-0.067698	-0.025436
164.0832	10	12	2	-0.023164	0.014081	-0.091751	-0.067698	-0.025436
166.0625	9	10	3	-0.001740	0.035954	-0.071154	-0.046811	-0.004039
166.0625	9	10	3	-0.001740	0.035954	-0.071154	-0.046811	-0.004039
168.0418	8	8	4	0.019684	0.057828	-0.050557	-0.025924	0.017358
168.0418	8	8	4	0.019684	0.057828	-0.050557	-0.025924	0.017358
178.0625	10	10	3	0.002651	0.043069	-0.071780	-0.045678	0.000186
178.0625	10	10	3	0.002651	0.043069	-0.071780	-0.045678	0.000186
178.0625	10	10	3	0.002651	0.043069	-0.071780	-0.045678	0.000186
180.0418	9	8	4	0.024075	0.064942	-0.051183	-0.024791	0.021582
180.0781	10	12	3	-0.012212	0.028664	-0.087485	-0.061087	-0.014705
182.0574	9	10	4	0.009213	0.050537	-0.066888	-0.040200	0.006692
182.0574	9	10	4	0.009213	0.050537	-0.066888	-0.040200	0.006692
194.0574	10	10	4	0.013603	0.057652	-0.067513	-0.039067	0.010916

196.0731	10	12	4	-0.001359	0.043147	-0.083318	-0.054576	-0.004074
198.0523	9	10	5	0.020165	0.065120	-0.062621	-0.033589	0.017423
208.0731	11	12	4	0.003031	0.050261	-0.083943	-0.053443	0.000151
208.0731	11	12	4	0.003031	0.050261	-0.083943	-0.053443	0.000151
208.0731	11	12	4	0.003031	0.050261	-0.083943	-0.053443	0.000151
210.0887	11	14	4	-0.011831	0.035856	-0.099649	-0.068852	-0.014740
224.0680	11	12	5	0.013984	0.064844	-0.079677	-0.046831	0.010882
244.0737	14	12	4	0.015604	0.071005	-0.086420	-0.050641	0.012224
246.0892	14	18	4	0.000841	0.056700	-0.102025	-0.065951	-0.002566
258.0892	15	14	4	0.005232	0.063814	-0.102650	-0.064817	0.001658
260.1044	15	16	4	-0.009231	0.049809	-0.117955	-0.079827	-0.012832
270.0889	16	14	4	0.009922	0.071229	-0.102975	-0.063384	0.006183
272.1048	16	16	4	-0.005240	0.056524	-0.118980	-0.079093	-0.009007
274.0841	15	14	5	0.016184	0.078397	-0.098384	-0.058206	0.012389
274.0845	15	14	5	0.015784	0.077998	-0.098784	-0.058606	0.011989
274.1201	16	18	4	-0.019803	0.042419	-0.134385	-0.094203	-0.023598
288.0999	16	16	5	0.005512	0.070907	-0.114914	-0.072682	0.001523
298.1200	18	18	4	-0.010922	0.056748	-0.135536	-0.091835	-0.015049
298.1200	18	18	5	-0.010922	0.056748	-0.135536	-0.091835	-0.015049
300.0992	17	16	5	0.010603	0.078721	-0.114839	-0.070848	0.006448
302.0790	16	14	6	0.031527	0.100095	-0.094742	-0.050461	0.027345
304.0950	16	16	6	0.016265	0.085290	-0.110848	-0.066271	0.012054
312.0992	18	16	5	0.014993	0.085836	-0.115465	-0.069715	0.010672
314.1152	18	18	5	-0.000269	0.071031	-0.131570	-0.085524	-0.004618
316.1309	18	20	5	-0.015232	0.056526	-0.147375	-0.101034	-0.019608
322.1204	20	18	4	-0.002540	0.070577	-0.137187	-0.089968	-0.007000
326.1149	19	18	5	0.004421	0.078445	-0.131895	-0.084091	-0.000094
328.1306	19	20	5	-0.010541	0.063940	-0.147700	-0.099600	-0.015084
330.0741	17	14	7	0.046670	0.121592	-0.091301	-0.042917	0.042100
330.1098	18	18	6	0.010983	0.085914	-0.127003	-0.078613	0.006413
332.1261	18	20	6	-0.004579	0.070809	-0.143408	-0.094723	-0.009177
336.0995	20	16	5	0.023475	0.099765	-0.117015	-0.067747	0.018821
336.1263	20	20	6	-0.003316	0.072981	-0.143817	-0.094545	-0.007969
340.0942	19	16	6	0.030236	0.107433	-0.111924	-0.062070	0.025528
340.1305	20	20	5	-0.006050	0.071155	-0.148226	-0.098367	-0.010760
342.1098	19	18	6	0.015374	0.093028	-0.127629	-0.077480	0.010637
344.1255	19	20	6	0.000411	0.078523	-0.143434	-0.092989	-0.004353
350.0786	20	14	6	0.049489	0.128953	-0.096844	-0.045527	0.044643
350.1150	21	18	5	0.013103	0.092574	-0.133246	-0.081923	0.008255
352.0942	20	16	6	0.034627	0.114548	-0.112549	-0.060936	0.029752
352.1308	21	20	5	-0.001960	0.077969	-0.149151	-0.097533	-0.006835
354.1464	21	22	5	-0.016822	0.063564	-0.164856	-0.112942	-0.021725
358.1411	20	22	6	-0.010061	0.071233	-0.159764	-0.107265	-0.015019
358.1411	20	22	6	-0.010061	0.071233	-0.159764	-0.107265	-0.015019
362.1725	20	26	6	-0.039986	0.042223	-0.191374	-0.138284	-0.045000

363.1075	18	19	8	0.025357	0.107777	-0.126423	-0.073196	0.020329
368.1255	21	20	6	0.009193	0.092752	-0.144684	-0.090722	0.004096
372.1212	20	20	7	0.014955	0.099421	-0.140593	-0.086044	0.009803
376.1517	20	24	7	-0.014071	0.071311	-0.171303	-0.116164	-0.019279
378.1460	23	22	5	-0.007641	0.078193	-0.165707	-0.110275	-0.012876
380.1466	19	24	8	-0.007509	0.078779	-0.166411	-0.110686	-0.012772
384.1204	21	20	7	0.020145	0.107335	-0.140418	-0.084111	0.014827
386.1361	21	22	7	0.005182	0.092830	-0.156223	-0.099620	-0.000164
388.1517	21	24	7	-0.009680	0.078425	-0.171928	-0.115030	-0.015054
388.1517	21	24	7	-0.009680	0.078425	-0.171928	-0.115030	-0.015054
390.1321	20	22	8	0.010644	0.099199	-0.152431	-0.095243	0.005243
394.1405	23	22	6	0.003711	0.093176	-0.161040	-0.103264	-0.001746
396.1205	22	20	7	0.024436	0.114350	-0.141143	-0.083077	0.018951
396.1567	23	24	6	-0.011751	0.078171	-0.177345	-0.119274	-0.017236
402.1309	21	22	8	0.016235	0.107513	-0.151857	-0.092909	0.010667
406.1623	21	26	8	-0.013690	0.078503	-0.183467	-0.123928	-0.019314
418.1623	22	26	8	-0.009300	0.085618	-0.184092	-0.122795	-0.015089
420.1568	25	24	6	-0.003070	0.092300	-0.178696	-0.117106	-0.008887
422.1362	24	22	7	0.018254	0.114074	-0.158199	-0.096319	0.012410
432.1204	25	20	7	0.037707	0.135793	-0.142919	-0.079576	0.031725
436.1728	22	28	9	-0.013210	0.085796	-0.195531	-0.131593	-0.019249
452.1829	26	28	7	-0.017452	0.085188	-0.206465	-0.140181	-0.023712
454.1986	26	30	7	-0.032414	0.070683	-0.222270	-0.155690	-0.038703
464.1829	27	28	7	-0.013061	0.092302	-0.207090	-0.139047	-0.019488
480.1784	27	28	8	-0.002709	0.106285	-0.203424	-0.133036	-0.009357
483.2014	27	31	8	-0.024603	0.085078	-0.226581	-0.155750	-0.031292
484.2083	27	32	8	-0.031134	0.078775	-0.233534	-0.162555	-0.037838
490.1622	28	26	8	0.017144	0.128404	-0.187744	-0.115892	0.010358
492.1779	28	28	8	0.002182	0.113900	-0.203549	-0.131402	-0.004632
494.1936	28	30	8	-0.012781	0.099395	-0.219354	-0.146912	-0.019623
508.1728	28	28	9	0.013134	0.128482	-0.199283	-0.124791	0.006099
510.2239	29	34	8	-0.037215	0.078599	-0.250490	-0.175697	-0.044279
522.1885	29	30	9	0.002562	0.121092	-0.215713	-0.139167	-0.004667
536.2041	30	32	9	-0.007910	0.113802	-0.232044	-0.153443	-0.015333
537.2119	30	33	9	-0.015341	0.106599	-0.239896	-0.161148	-0.022778
550.1834	30	30	10	0.017905	0.142789	-0.212072	-0.131422	0.010288
552.1866	26	32	13	0.015438	0.140777	-0.215377	-0.134433	0.007793
552.2009	30	32	10	0.001143	0.126486	-0.229677	-0.148731	-0.006502
554.2147	30	34	10	-0.011920	0.113880	-0.243582	-0.162341	-0.019593
556.2319	30	36	10	-0.028382	0.097875	-0.260887	-0.179351	-0.036083
558.2475	30	38	10	-0.043244	0.083470	-0.276592	-0.194760	-0.050973
568.1938	30	32	11	0.014095	0.143067	-0.223411	-0.140121	0.006228
570.2120	30	34	11	-0.003367	0.126064	-0.241716	-0.158130	-0.011261
572.2253	30	36	11	-0.015930	0.113958	-0.255121	-0.171240	-0.023852
578.1783	31	30	11	0.033248	0.164487	-0.208431	-0.123677	0.025243

580.1940	31	32	11	0.018286	0.149982	-0.224236	-0.139187	0.010253
582.2107	31	34	11	0.002324	0.134478	-0.241041	-0.155696	-0.005737
584.2253	31	36	11	-0.011539	0.121072	-0.255746	-0.170106	-0.019628
584.2253	31	36	11	-0.011539	0.121072	-0.255746	-0.170106	-0.019628
586.2421	31	38	11	-0.027601	0.105468	-0.272651	-0.186715	-0.035718
602.2358	31	38	12	-0.015449	0.121250	-0.267185	-0.178904	-0.023787
606.2249	37	34	8	-0.003090	0.134515	-0.256493	-0.167628	-0.011483
614.2367	32	38	12	-0.011958	0.127465	-0.268710	-0.178671	-0.020462
614.2371	32	38	12	-0.012358	0.127065	-0.269110	-0.179071	-0.020862
614.2372	32	38	12	-0.012458	0.126965	-0.269210	-0.179171	-0.020962
628.2303	36	36	10	-0.000438	0.142161	-0.263039	-0.170949	-0.009136
628.2526	33	40	12	-0.022730	0.119875	-0.285341	-0.193247	-0.031428
630.2320	32	38	13	-0.001406	0.141648	-0.264844	-0.172460	-0.010131
632.2464	32	40	13	-0.015069	0.128443	-0.279349	-0.186669	-0.023822
632.2612	36	40	10	-0.029863	0.113651	-0.294150	-0.201468	-0.038617
636.1991	37	32	10	0.033677	0.178086	-0.232255	-0.138996	0.024869
644.2473	33	40	13	-0.011578	0.134658	-0.280874	-0.186435	-0.020497
654.2460	38	38	10	-0.006620	0.141886	-0.280095	-0.184191	-0.015677
660.2418	33	40	14	-0.000226	0.149640	-0.276208	-0.179424	-0.009367
662.2570	33	42	14	-0.014688	0.135635	-0.291513	-0.194434	-0.023857
684.2202	38	36	12	0.030148	0.185456	-0.255857	-0.155559	0.020675
748.2745	40	44	14	-0.000716	0.169133	-0.313495	-0.203808	-0.011075
751.2597	39	43	15	0.015177	0.185702	-0.298851	-0.188726	0.004775
756.2413	41	40	14	0.035399	0.207056	-0.280711	-0.169855	0.024929
778.2841	41	46	15	0.000664	0.177324	-0.324659	-0.210573	-0.010111
779.2546	40	43	16	0.030520	0.207400	-0.295210	-0.180981	0.019731
780.2999	41	48	15	-0.014398	0.162720	-0.340564	-0.226182	-0.025201
788.2828	46	44	12	0.005623	0.184552	-0.323880	-0.208328	-0.005291
810.3101	42	50	16	-0.013618	0.170312	-0.352328	-0.233547	-0.024836
810.3102	42	50	16	-0.013718	0.170212	-0.352428	-0.233647	-0.024936
826.3060	42	50	17	-0.003665	0.183895	-0.349062	-0.227936	-0.015105
830.2570	47	42	14	0.046781	0.235238	-0.300268	-0.178562	0.035286
840.3219	43	52	17	-0.014437	0.176305	-0.365692	-0.242512	-0.026071
860.3039	49	48	14	0.010875	0.206152	-0.348734	-0.222624	-0.001036
876.2988	49	48	15	0.021827	0.220735	-0.344467	-0.216013	0.009695
890.3145	50	50	15	0.011255	0.213344	-0.360898	-0.230389	-0.001071
906.2883	53	46	14	0.043300	0.249015	-0.335530	-0.202679	0.030752
950.3145	55	50	15	0.033208	0.248917	-0.364025	-0.224720	0.020051
956.3098	50	52	19	0.040102	0.257171	-0.359637	-0.219454	0.026862
1038.3305	58	54	18	0.049412	0.285099	-0.384611	-0.232405	0.035037
1054.3254	58	54	19	0.060365	0.299682	-0.380345	-0.225794	0.045768
1082.3720	64	58	16	0.024026	0.269710	-0.428407	-0.269744	0.009041
1098.3669	65	60	17	0.034979	0.284293	-0.424140	-0.263133	0.019772
1200.4045	61	68	25	0.034713	0.307189	-0.467058	-0.291093	0.018094
1216.3995	61	68	26	0.045565	0.321672	-0.462891	-0.284582	0.028725

1232.4657	63	76	25	-0.014756	0.264997	-0.529929	-0.349264	-0.031819

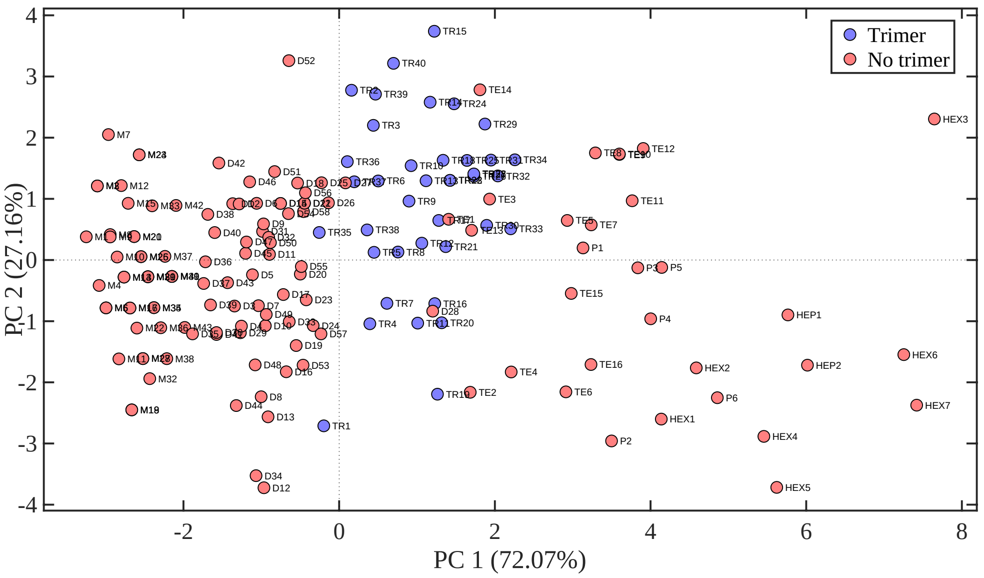

Figure S1. Scores plot of the KMD-PCA-QDA classification model for lignin trimers showing principal component (PC) 1 and 2.

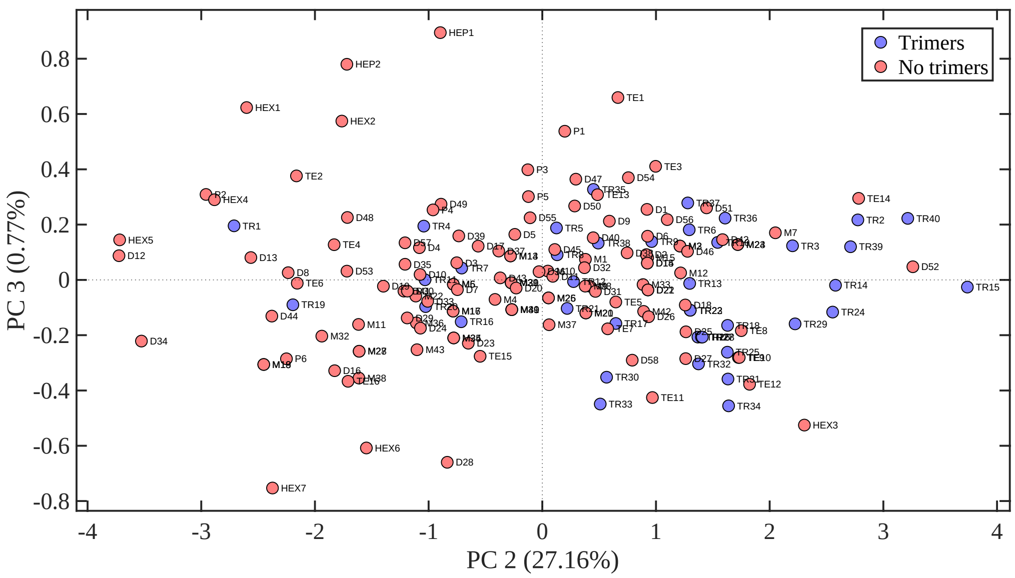

Figure S2. Scores plot of the KMD-PCA-QDA classification model for lignin trimers showing principal component (PC) 2 and 3.

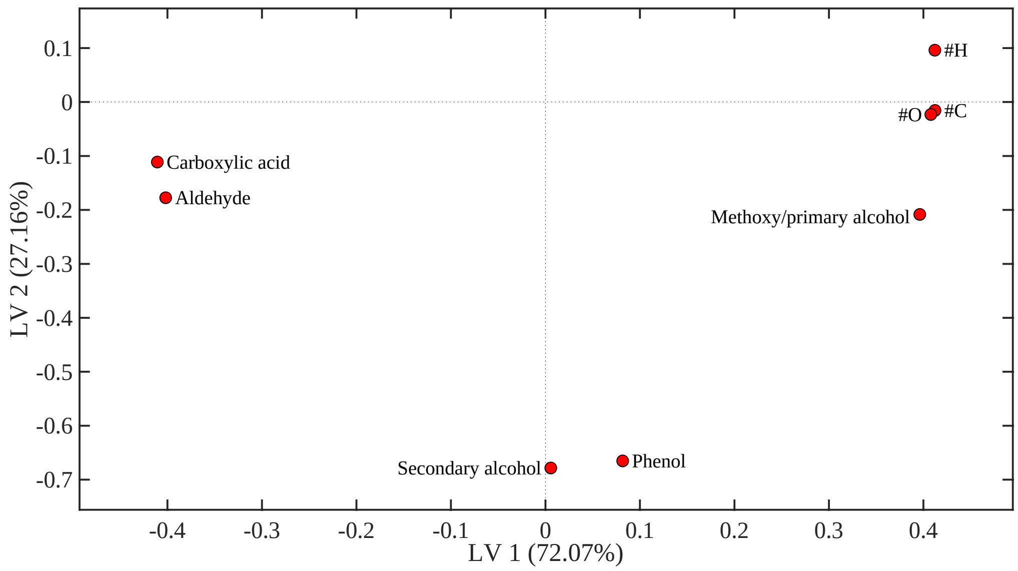

Figure S3. Loading plot of the KMD-PCA-QDA classification model for lignin trimers showing latent variable (LV) 1 and 2.

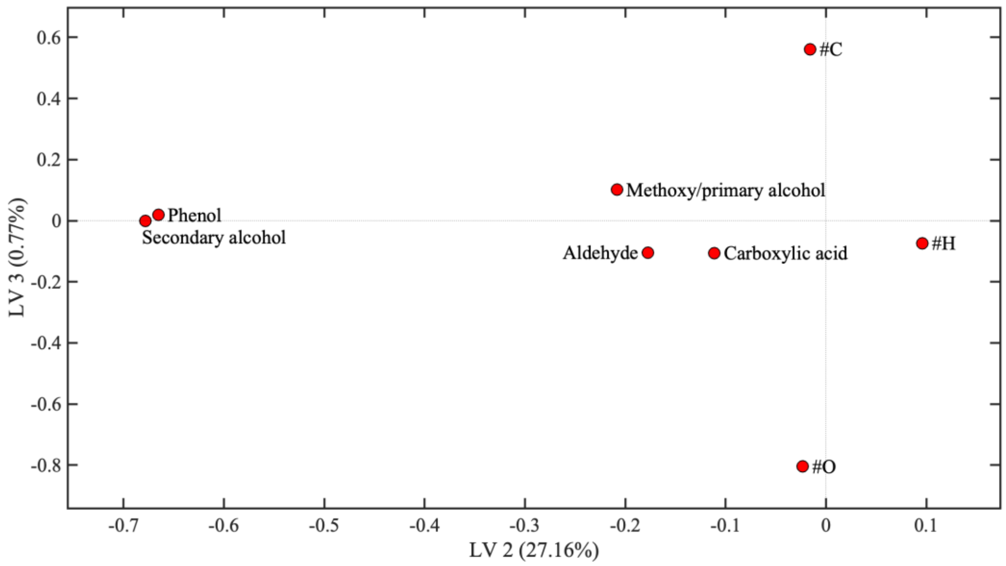

Figure S4. Loading plot of the KMD-PCA-QDA classification model for lignin trimers showing latent variable (LV) 2 and 3.

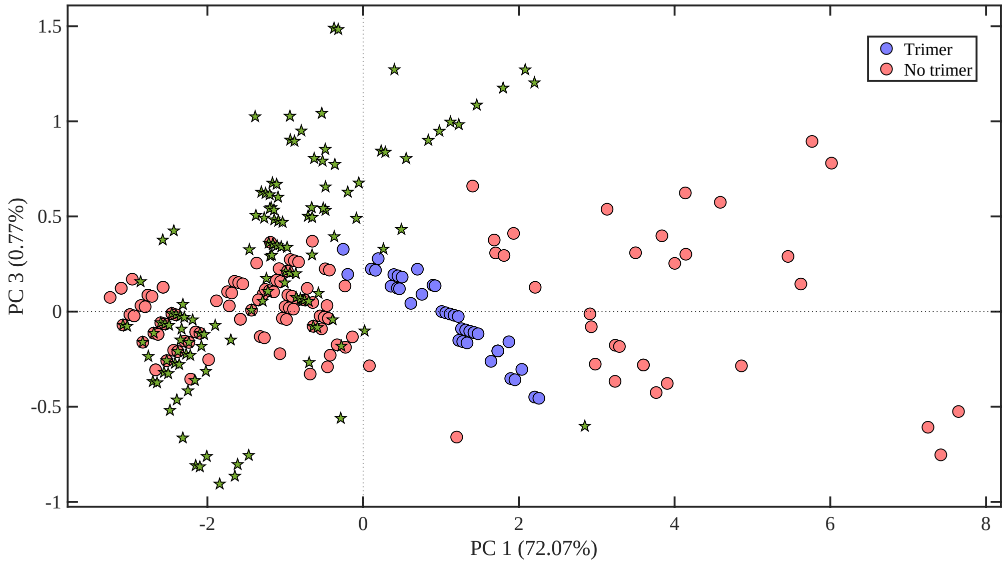

Figure S5. Scores plot of the KMD-PCA-QDA classification model for lignin trimers showing principal component (PC) 1 and 3 including the 133 m/z values from the Lignosulphonate lignin sample (green stars).

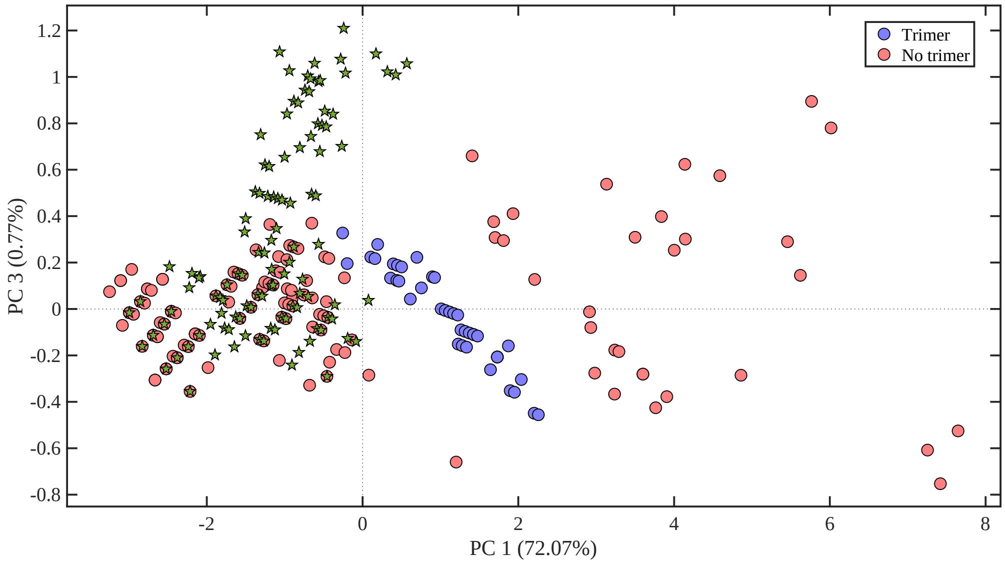

Figure S6. Scores plot of the KMD-PCA-QDA classification model for lignin trimers showing principal component (PC) 1 and 3 including the 112 m/z values from the depolymerised Kraft lignin sample (green stars).
